# The past, present, and future for constitutional ring chromosomes: A report of the international consortium for human ring chromosomes

**DOI:** 10.1016/j.xhgg.2022.100139

**Published:** 2022-09-10

**Authors:** Peining Li, Barbara Dupont, Qiping Hu, Marco Crimi, Yiping Shen, Igor Lebedev, Thomas Liehr

**Affiliations:** 1 Clinical Cytogenetics Laboratory, Department of Genetics, Yale School of Medicine, New Haven, CT, USA; 2 Cytogenetics Laboratory, Greenwood Genetic Center, Greenwood, SC, USA; 3 Department of Cell Biology and Genetics, Institute of Basic Medicine, Guangxi Medical University, Nanning, Guangxi, China; 4 Ring 14 International, Via Santa Maria Alla Porta 2, 20123 Milano, Italy; 5 Kaleidos SCS, Scientific Office, Via Moretti Andrea 20, 24121 Bergamo, Italy; 6 Division of Genetics and Genomics, Boston Children’s Hospital, Department of Neurology, Harvard Medical School, Boston, MA, USA; 7 Laboratory of Ontogenetics, Research Institute of Medical Genetics, Tomsk National Research Medical Center of the Russian Academy of Sciences, Tomsk 634050, Russia; 8 Jena University Hospital, Friedrich Schiller University, Institute of Human Genetics, 07747 Jena, Germany

**Keywords:** constitutional ring chromosomes, dynamic mosaicism, ring syndrome, chromosome microarray analysis, whole-genome sequencing, clinical heterogeneity, cytogenomic heterogeneity, cellular reprogramming, chromosome therapy, patient advocacy organizations

## Abstract

Human ring chromosomes (RCs) are rare diseases with an estimated newborn incidence of 1/50,000 and an annual occurrence of 2,800 patients globally. Over the past 60 years, banding cytogenetics, fluorescence *in situ* hybridization (FISH), chromosome microarray analysis (CMA), and whole-genome sequencing (WGS) has been used to detect an RC and further characterize its genomic alterations. Ring syndrome featuring sever growth retardation and variable intellectual disability has been considered as general clinical presentations for all RCs due to the cellular losses from the dynamic mosaicism of RC instability through mitosis. Cytogenomic heterogeneity ranging from simple complete RCs to complex rearranged RCs and variable RC intolerance with different relative frequencies have been observed. Clinical heterogeneity, including chromosome-specific deletion and duplication syndromes, gene-related organ and tissue defects, cancer predisposition to different types of tumors, and reproductive failure, has been reported in the literature. However, the patients with RCs reported in the literature accounted for less than 1% of its occurrence. Current diagnostic practice lacks laboratory standards for analyzing cellular behavior and genomic imbalances of RCs to evaluate the compound effects on patients. Under-representation of clinical cases and lack of comprehensive diagnostic analysis make it a challenge for evidence-based interpretation of clinico-cytogenomic correlations and recommendation of follow-up clinical management. Given recent advancements in genomic technologies and organized efforts by international collaborations and patient advocacy organizations, the prospective of standardized cytogenomic diagnosis and evidence-based clinical management for all patients with RCs could be achieved at an unprecedented global scale.

## Teaser

Significant advances in the genetic diagnosis and clinical management for constitutional ring chromosomes have been achieved in the past 60 years. Li et al. performed a systematic review on the progress of diagnostic technologies for analyzing ring chromosomes and the current understanding of clinical and cytogenomic heterogeneity of ring chromosomes. An international consortium for human ring chromosomes is working toward standardized comprehensive genetic diagnosis and evidence-based genetic counseling and clinical management for patients with this rare and unique chromosomal rearrangement.

## Historical perspective of human ring chromosomes

### Human ring chromosomes by solid staining

A ring chromosome (RC) results from breakage and fusion at the telomeric or distal regions of both chromosome arms; this circular chromosome replaces one normal chromosome and presents unique mitotic behavior in *Drosophila* and maize.^1^^,^^2^ In 1962, a human constitutional RC derived from sex chromosome X was first observed and followed by several patients showing clinical association with some characteristics of Turner syndrome.^3^, ^4^, ^5^ From 1962 to 1970, earlier studies by solid staining of metaphase chromosomes detected more than 30 patients with an RC involving chromosomes X, 1, 2, 3, 4, 5, 13, 16, and 18 and a chromosome of the C, D, and E groups.^6^, ^7^, ^8^, ^9^, ^10^, ^11^, ^12^ To characterize each RC, autoradiographic studies with the incorporation of tritiated thymidine into cultured blood leukocytes was performed to present chromosome specific replication patterns.^9^^,^^12^, ^13^, ^14^ Mitotic behavior of an RC for the frequency of dicentric ring, loss of ring, and sister chromatid exchange (SCE) was analyzed by solid staining and autoradiographic patterns.^15^ A patient with an RC 4 showed normal intelligence and short stature, indicating the possibility of mild phenotypes for RCs.^14^ Despite the technical difficulty and analytical limitation in the identification of each RC, these earlier studies made several important findings. Firstly, the association of RCs with congenital malformations to mild phenotype was observed, and the clinical features of RCs seemed to be related to those of distal deletions in the short and long arms of the involved chromosomes.^10^, ^11^, ^12^, ^14^ Secondly, self-perpetuating RCs were reported in different ethnic groups of patients in Europe, Australia, and North America, and variabilities in the size and cellular frequency of RCs were noted in blood leukocytes and skin fibroblasts.^10^^,^^11^ Thirdly, the first mother-to-daughter transmission of an RC 18 was observed, indicating possible fertility and segregation of RCs in families.^12^

### Banding and molecular cytogenetics for dynamic mosaicism and ring syndrome

In 1972, Q banding stained by quinacrine mustard was used to detect an RC 20 in a patient with intellectual disability, seizures, microcephaly, and behavior problems.^16^ Soon after this report, Q banding and G banding by Giemsa staining were introduced to analyze RCs and their variants in 60–250 metaphase cells.^17^^,^^18^ In 1973, a girl with an RC 1 showed severe short stature, microcephaly, and intellectual disability and died with acute myeloid leukemia and bronchopneumonia at the age of 9 years; her bone marrow metaphases showed an absence of RC 1 and probably the presence of an abnormal chromosome 1 and a small marker chromosome.^19^ This was the first patient with somatic chromosomal rearrangements derived from a constitutional RC associated with a specific type of cancer. In 1975, the prenatal diagnosis of RCs by amniocentesis was reported in a fetus with an RC 13 and another with an RC 17; these pregnancies were terminated after genetic counseling for the probable outcome from reported patients.^20^^,^^21^ Later, combined Q/G banding, silver staining for nucleolus organizing region (NOR), and SCE performed on an RC 15 observed decreased satellite association, multiple ring variants including decondensed and pulverized rings, and unchanged rate of spontaneous SCE.^22^

Detailed analysis of mosaic patterns in various RCs revealed the behavior of an RC through mitosis and the derivative RC variants including a dicentric ring, an interlocked ring, a small ring, and a loss of the entire RC.^23^^,^^24^ A mathematic model was proposed to estimate the survival rates to the next mitosis of various chromosomal abnormalities including centric and acentric RCs as well as dicentric and tricentric fragments.^25^ The consequence of the irregular but persistent generation of genetically different cells resulting from the behavioral peculiarities of RCs were described as “dynamic mosaicism.”^26^ This RC-induced mosaicism should be differentiated from the “true mosaicism” consisting of cells with a normal complement and an abnormal RC.

In 1981, the term ring syndrome was proposed based on a hypothesis that cells with further chromosomal anomalies from the original ring are less likely to survive.^27^ The abnormal phenotypes will usually be a mixture of three main effects from (1) the original distal or telomeric deletions that accompanied the ring formation, (2) the further aneuploidies produced by the ring mechanics, and (3) the massive cell death and the ensuing enormous waste of metabolism. The latter can be expected to be the same in all patients with an RC.^27^ On the analysis of 207 patients with a ring autosome, approximately 20% of them showed extreme growth failure, no major malformations, none or only a few unspecific minor anomalies, and/or otherwise almost normal appearance, which could be regarded as ring syndrome.^28^ Furthermore, severe growth failure was seen significantly more often among patients with a ring of large chromosomes than among patients with a smaller ring, larger RCs showed significantly more instability than smaller rings, and growth failure was present significantly more in patients with an unstable ring than with a stable ring.^28^ Ring syndrome probably described a baseline abnormality of complete RCs; however, variable phenotypes among patients with an RC of the same chromosome, and specific phenotypes related to deletions were noted.^18^^,^^29^ Banding cytogenetics enabled the characterization of RCs and their variants, but the low analytical resolution of G bands cannot reliably distinguish a complete ring from an incomplete one.

In 1988, fluorescence *in situ* hybridization (FISH) using X and Y centromeric probes was used to rapidly detect the origin of sRCs derived from chromosomes X and Y.^30^^,^^31^ Loss of telomeric sequences in an RC 20 was detected by FISH using probes specific for centromeric and telomeric sequences.^32^ An RC 15, r(15) (p12q26.3), involving a deletion of the insulin-like growth factor 1 receptor gene (*IGF1R*) at 15q26.3, was detected by FISH, which correlated with severe prenatal and postnatal growth deficiency and Silver-Russell syndrome-like features.^33^

FISH has also been effective in the identification of chromosomal origin of small RCs (SRCs) and supernumerary small ring or marker chromosomes (sSRCs/sSMCs) of pediatric and prenatal patients, which facilitated the interpretation of clinical outcome for genetic counseling.^34^^,^^35^ Mosaicism of sSRCs of chromosome 1 resulting in partial trisomy of different segments of chromosome 1 was reported in three of eight patients with an RC 1; a normal phenotype was noted in one patient of sSRC 1 composed primarily of the centromere and the heterochromatic regions of chromosome 1.^36^

### Genomic approaches for RCs

In 2003, array comparative genomic hybridization (aCGH) was performed in a patient with a satellited ectopic NOR on the distal 1p generated by the breakage event during the formation of an RC 21.^37^ Further improved high-density aCGH provided cytogenomic mapping in a patient of a complete RC 19 and a duplication in 2q and another patient of an RC 4 with a deletion of 4p for Wolf-Hirschhorn syndrome (OMIM: 194190).^38^^,^^39^ The application of high-resolution oligonucleotide aCGH precisely delineated segmental duplication and deletion in a patient with an RC 14. This facilitated the comparison of clinical features with patients carrying distal alterations of chromosome 14 and the proposal of an alternative chromosome rescue mechanism for RC formation.^40^ Genome-wide SNP array was also validated for analyzing genomic structure of RCs and sSMCs.^41^^,^^42^ Technical standards and practice guidelines for chromosome microarray analysis (CMA) by aCGH and SNP array on constitutional cytogenetic abnormalities have been developed and implemented.^43^^,^^44^

**Figure 1:**
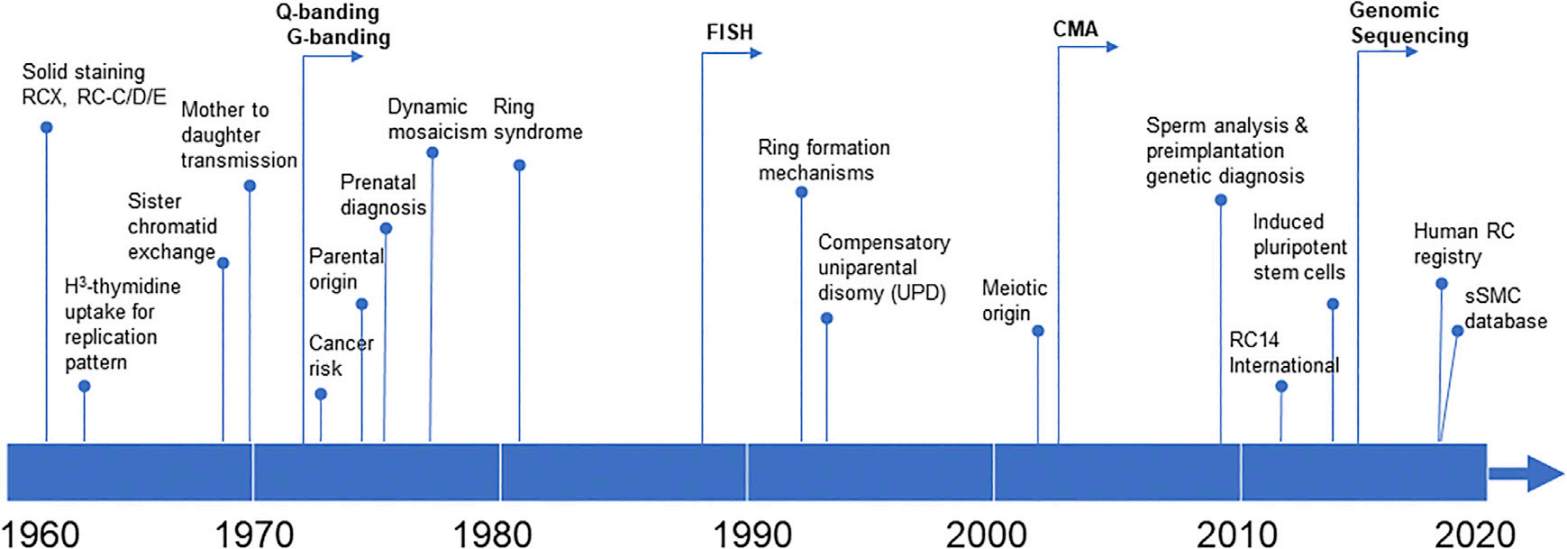
Major technology advancements and important clinical findings on the study of human RCs denoted along the top of a timeline for the past 60 years

In 2015, an acentric RC 14 arising from an interstitial excision was analyzed by whole-genome sequencing (WGS) to characterize the breakpoints and fusion sequence at the base-pair level. The breakpoints occurred at non-coding RNA genes of unknown function; the proximal breakpoint downstream of the *FOXG1* gene may have resulted in its dysregulation and contributed to the phenotype.^45^ WGS defined the breakpoints at distal long and short arms for two patients of RC 18 and one patient of RC 6.^46^, ^47^, ^48^ WGS in an RC 22 defined an unexpected chromothripsis event.^49^ WGS on a patient of RC 9 with distal deletions in both arms and an interstitial duplication in the long arm revealed RC formation mechanism by intra-strand repairing of subtelomeric double-strand breaks.^50^ Reprograming of patient-derived fibroblast cell lines with RCs to induced pluripotent stem cells (iPSCs) discovered cell-autonomous correction of RC for potential chromosome therapy.^51^^,^^52^ Recently, transcriptome analysis by RNA sequencing was performed to reveal differentially expressed genes in RC 20.^53^ A comprehensive analysis of human RCs should be performed combing cell-based G banding and FISH analyses for detecting ring variants and dynamic mosaicism with DNA-based CMA and WGS for identifying genomic alterations. The timeline for advancements of genetic technologies and major cytogenomic and clinical findings of RCs is shown in Figure 1.

## Current understanding of constitutional RCs

### Overview of clinical cases of RCs

Constitutional RCs belong to a rare type of intra-chromosome structural abnormality with an estimated newborn incidence of 1 in 50,000.^54^^,^^55^ Based on the current population of 7.7 billion and the birth rate of 1.8%, it is estimated that there is an annual newborn number of 2,800 patients with an RC globally. With an estimated detection rate of 0.06% in current cytogenetics laboratories, almost all laboratories have occasionally encountered a few cases with an RC but very rarely collected a case series of specific RCs.^56^ A systematic literature search of Chinese patients with an RC from 1979–2017 found 94 case reports and four original articles with a total of 113 patients, including 95 patients of autosomal rings and 18 patients of sex chromosome rings. It is estimated that these reported patients in the Chinese population accounted for approximately 1% of the occurrence for all RCs. The cytogenetic and clinical findings from these patients were used as a dataset for the development of an online registry for human RCs.^57^

**Figure 2:**
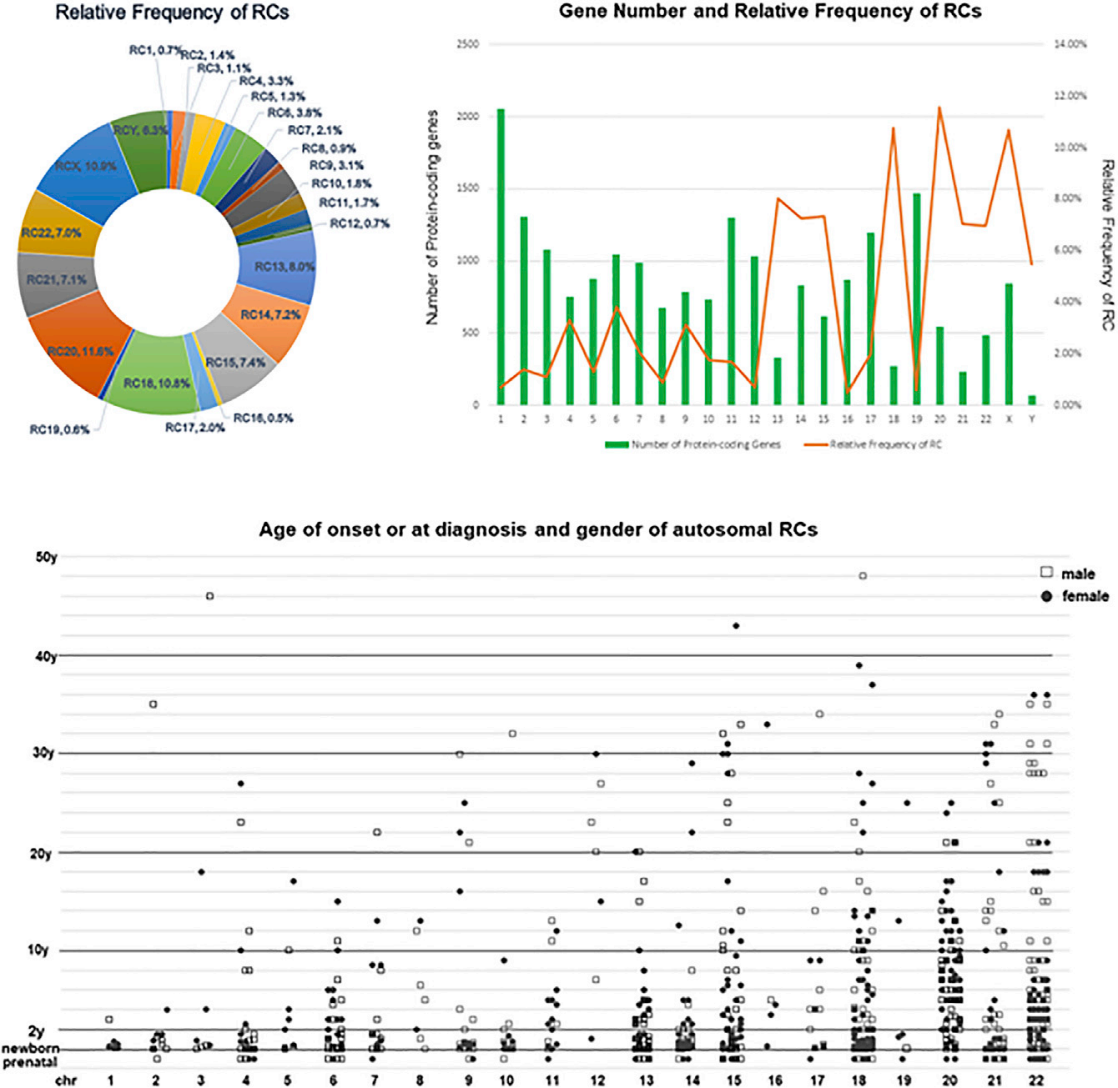
Relative frequency and case distribution of human RCs (1) Relative frequency of ring autosomes and sex chromosomes. (2) Correlation of relative frequency of RCs and number of protein-coding genes in each chromosome. (3) Distribution of cases with autosomal RCs by their age of onset or at diagnosis and genders.

A further search of PubMed since 1962 retrieved 854 patients of autosomal rings and 175 patients of sex chromosome rings from 878 publications (Table S1). A significant uneven occurrence of RCs was noted with relative frequencies of 10%–12% for RCs 18, 20, and X, 5%–9% for chromosomes of D/G groups (13, 14, 15, 21, 22) and Y, and less than 4% for the remaining other chromosomes. The least frequently seen RCs with a relative frequency less than 1% raised a question of “RC intolerance” for chromosomes 1, 8, 12, 16, and 19. The RC instability and intolerance could be chromosome specific or may act in a polygenic way. Accessible clinical and laboratory content was assessed in 727 autosomal RCs (Table S2). Approximately 9% (62 patients) of autosomal RCs were detected prenatally and 91% (665 patients) were postnatal patients. For pregnancies detected with an RC, following prenatal genetic counseling, 68% elected to terminate the pregnancy, 19% continued the pregnancy to term, and 13% of the pregnancies ended with stillbirth. For postnatal patients, parental studies performed on 325 families revealed approximately 88% of the RCs were *de novo*, 11% were from a maternal carrier, and less than 1% were from a paternal carrier. This estimation could be biased since 73% of familial patients with a maternal carrier were in RCs 15, 18, 20, and 21. The inheritability of RCs should be evaluated on individual chromosomes. CMA performed on 163 autosomal RCs showed that 8% had a complete RC, 71% had an incomplete RC with simple deletions at one or both ends of the chromosome, and 21% had complex genomic rearrangements from combined deletions/duplications to chromothripsis. Survival to adulthood with age ranging from 18 to 67 years was noted in 140 patients in the probands or carrier parents. Of the 35 deceased patients in the reports, 25 patients (71%) died neonatally or within the first year of life due to abnormalities of cardiac, respiratory, and renal systems, nine patients (26%) died during childhood and teenage years, and one patient (3%) died at the age of 27 years. A longitudinal study is needed to understand the impact of RCs on life expectation. Shown in Figure 2 are the relative frequencies of RCs, the correlation of relative frequency with the number of genes involved for each chromosome, the distribution of age of onset or age at diagnosis, and the genders for autosomal RCs. These 1,029 patients reported in PubMed accounted for approximated 0.6% of the occurrence for all RCs worldwide. Publication bias toward patients with an RC with more laboratory analyses and more severe clinical features should be noted. As for many rare genetic diseases, under-representation of reported cases in the literature and publication bias toward more severe cases are a challenge for accurate prospective counseling in the situation of prenatal or early-in-life diagnosis of an RC.

### Cytogenomic heterogeneity and ring formation mechanisms

Structurally, RCs are divided into two types: (1) complete RCs without loss of genetic material by telomere-to-telomere fusion, and (2) incomplete RCs with distal or interstitial deletions and/or duplications by one or multiple fusion events.^58^, ^59^, ^60^, ^61^ Mechanisms for RC formation were firstly explored in two small case series of RC 21. RC formation mechanisms included breakage and fusion events in distal regions with deletions of 21q and in an intermediate isochromosome and an SCE in the initial ring for double-sized dicentric RC 21; the latter two could associate with a Down syndrome phenotype.^62^^,^^63^ A small case series of 14 patients with various RCs showed that 12 RCs (12/14, 86%) involved distal breaks and loss of chromosomal material and only two RCs (2/14, 14%) were considered as complete rings.^64^ Duplications in addition to terminal deletions were noted in a proportion of RCs, suggesting that inverted duplication at the joined ends for the formation of a mirror-dicentric RC.^65^

Molecular mechanisms for RC formation have been defined from different RCs by genomic analysis. Telomeric and subtelomeric fusions for complete rings were confirmed in an RC 17 and an RC 4.^66^^,^^67^ Intra-chromosome repair for distal breaks forming incomplete rings with distal deletions and interstitial copy-number alterations were characterized in an RC 18 and an RC 6 by WGS.^46^, ^47^, ^48^ The formation of an RC 9 with distal deletions and an interstitial duplication probably used an alternative end jointing mechanism involving inverted repeats induced interstrand fold back followed by microhomology-mediated DNA synthesis and ligation.^50^ Rare incidence of chromothripsis involving multiple segments in an RC 22 was characterized by WGS.^49^ The impact of NOR association in nuclei on RC formation has been observed in patients of RC 15 and RC 21 as well as in families with offspring of an RC from parental carriers of a Robertsonian translocation.^22^^,^^37^,^68^^,^^69^ These results indicated multiple DNA-repairing mechanisms in forming RCs; the repairing could be a one-step event as seen in most patients with an RC and two-step events involving an initial RC going through a breakage-fusion-bridge cycle in mitosis for a secondary RC.^57^ The observation of a small RC 3 and a supernumerary acrocentric fragment of distal portions of 3p and 3q suggested a novel mechanism for the origin of sSMC.^70^ It is hypothesized that some sSMCs and sSRCs are the residual chromosomal markers on the rescue of trisomy or large RCs. sSRCs as the rescue resultant of RCs were seen more frequently in chromosomes X and Y and large autosomes.^36^ Loss of the RC followed by monosomy compensatory for a mosaic pattern consisting of RC and normal karyotype has been observed in patients of RC 21 and RC 8; this mechanism resulted in uniparental isodisomy of the normal chromosome.^71^^,^^72^ Molecular analysis should be introduced to differentiate cells with isodisomy due to monosomy compensatory in dynamic mosaicism from cells with heterodisomy of true mosaicism. The risk of uniparental disomy (UPD)-related disorders should be taken into consideration.^73^

Mitotic disturbance of dynamic mosaicism resulting from RC instability have been well documented, but chromosomal and genomic factors that contribute to the variability of this instability remain elusive.^60^ Reduced cell viability of 10%–20% in fibroblasts with RCs 4 and 15 and decreased cloning efficiency of 50% in fibroblasts with RC 15 were noted in *in vitro* cell culture.^74^ Cytogenetic detection of RCs was performed on cultured cells from amniocentesis chorionic villi, and peripheral blood lymphocytes, but rarely on multiple tissues. Prenatal analysis in a patient noted complete karyotype discrepancy with normal male karyotype in cultured chorionic villi, RC 18 and monosomy 18 mosaicism in amniocytes, and RC 18 only in umbilical blood lymphocytes.^75^ A patient was reported with tissue-specific mosaicism consisting of a stable RC 13 in lymphocytes and an unstable variant RC 13 in skin fibroblasts.^76^ These observations indicated the limitation on interpreting RC findings from the analysis of single tissue. There is no consensus from the experts for a standardized procedure to evaluate the RC instability and cellular viability in current diagnostic analysis.

### Clinical heterogeneity on disability, inheritance, and cancer predisposition

Ring syndrome with severe growth failure and variable intellectual disability for complete ring autosomes has been recognized.^27^^,^^28^ The patients reported in the literature showed clinical heterogeneity with compound effects from mitotic behavior and specific genomic imbalances in the RCs.^77^ Categorization of genomic copy-number variants based on their clinical impact on disability, penetrance, inheritance, and reproduction has been proposed from accumulated data of large case series.^78^ Obvious clinical correlations with distal deletion syndromes have been reported for different RCs. For example, recurrent patients showed RC 4 with Wolf-Hirschhorn syndrome, RC 5 with Cri-du-Chat syndrome (OMIM: 123450), RC 17 with Miller-Dieker syndrome (OMIM: 247200), and RC 22 with Phelan-McDermid syndrome (OMIM: 606232).^39^^,^^79^, ^80^, ^81^, ^82^ These syndromic manifestations could be so distinct to conceal the presentation of ring syndrome.

Defects in different systems such as cardiac and muscle defects, ocular and dental anomalies, and various brain and neurologic defects like autism, bipolar disorder, epilepsy, or seizures, and other tissue-specific effects have been reported in different RCs.^77^ Autism has been reported in RCs 13, 14, 17, 18, and 22.^83^, ^84^, ^85^, ^86^, ^87^, ^88^ Epilepsy and seizures have an extremely high penetrance for RC 14 syndrome (OMIM: 616606) and RC 20 but also are seen in other autosomal RCs such as 17, 18, and 21; this is a medically actionable condition, but drug resistance in some patients has been described and alternative treatments suggested.^89^, ^90^, ^91^, ^92^, ^93^, ^94^, ^95^, ^96^ Cytogenomic mapping of patients with an RC 9 showed genotype-phenotype correlations of short-arm deletions of the *DOCK8* gene (OMIM: 611432) with developmental/intellectual disabilities and *DMRT* genes with sex reversal in XY females (OMIM: 158170), respectively, as well as long-arm deletion of the *EHMT1* gene with Kleefstra syndrome 1 (OMIM: 610253).^50^ Long-term follow up on monozygotic twins with an RC 13 mosaicism in one of them showed discordant phenotypes and a clear comparison between the twins.^97^ A 35-year follow up in a patient with an RC 2 updated a terminal deletion and revealed that the patient with ring syndrome features of severe growth failure and moderate intellectual disability could survive to adulthood without any new phenotypic data.^98^

Although familial patients of RCs have been reported, an earlier review estimated that inherited RCs were reported in 5.6% of patients but in reality should be no more than 1% considering publication bias.^69^ RC-associated infertility such as Turner syndrome in RC X and azoospermia in RC Y and other autosomes are well documented.^99^, ^100^, ^101^ RC 21 in likely healthy persons showed azoospermia in males and infertility in females.^102^ Possible maternal gonadal mosaicism for recurrence of RC 4 was reported.^103^ Tracking DNA polymorphic markers for two RCs 18 in a patient revealed that complex pairing and recombination event in meiosis resulted in the formation of RCs.^104^ Interchromosomal effects for increased risk of aneuploidies were suggested by examining sperm chromosomes in two male carriers of RC Y.^105^^,^^106^ Analysis of sperm chromosomes from patients carrying an RC 21 showed preferential meiosis of normal spermatogonia and thus significantly reduced the RC in mature sperm cells.^107^ Preimplantation genetic diagnosis of embryos from a maternal carrier of an RC 22 showed accumulated postzygotic errors of chromosome 22.^107^ RCs could be used to study the influence of chromosome size, morphology, and gene density on the distribution and segregation of bivalent chromosomes in meiosis.^108^

In additional to congenital anomalies, risks for specific cancers have been reported for RCs. RC 7 was seen in association with skin lesions and malignant melanoma.^109^^,^^110^ Patients of RC 11 associated with Wilms tumor, RC 13 with retinoblastoma, RC 17 with neurofibromatosis, RC 21 with acute myeloid leukemia, and RC 22 with neurofibromatosis, meningiomas, and vestibular schwannoma have been reported.^85^^,^^111^^,^^112^^,^^113^, ^114^, ^115^, ^116^, ^117^, ^118^ Dynamic mosaicism and dysfunction of harbored tumor suppressor genes in these constitutional RCs mediated the predisposition to cancer.^119^ Cancer surveillance should be considered for patients carrying these RCs. Changes in skin pigmentation and café au lait spots likely relating to dynamic mosaicism have been reported in several patients of different RCs.^38^^,^^120^ Constitutional RCs with cancer predisposition and related tumor-suppressor genes are listed in Table 1. Besides, RCs as acquired chromosomal abnormalities in human neoplasia such as giant RCs in liposarcoma have also been seen, which are reviewed elsewhere.^121^^,^^122^

**Table 1:** Cancer predisposition and related tumor-suppressor genes of constitutional RCs

RCs	Tumor-suppressor genes	Cancers	References
5	–	myelodysplastic syndrome	Nozawa et al.^81^
7	–	hyperpigmentation, melanoma	DeLozier-Blanchet et al.,^109^ Mehraein et al.^109^, ^110^
11	*WT1*	Wilms tumor	Carella et al.^111^
13	*RB1*	retinoblastoma	Morrissette et al.^112^
17	*TP53*, *NF1*	neurofibromatosis type 1	Havlovicova et al.^85^
21	*RUNX1*	acute myeloid leukemia	Burillo-Sanz et al.,^113^ Vormittag-Nocito et al.^114^
22	*NF2*	neurofibromatosis type II, meningiomas, schwannoma,	Tommerup et al.,^115^ Petrella et al.,^116^ Denayer et al.^117^

### Organized effort for cytogenomic diagnosis and clinical management

The observed cytogenomic and clinical heterogeneity of RCs demonstrated the necessity of an organized effort on a large case series for accurate clinic-cytogenomic correlation and evidence-based genetic counseling and clinical management. Ring 14 International (R14I) is a patient advocacy organization (PAO) founded in 2012 as a non-profit organization to help affected people and their caregivers and to promote and support scientific research projects. Recently, R14I managed an *ad hoc* task force to publish the first report on recommended guidelines for diagnosis and clinical management of Ring 14 syndrome.^89^ According to those guidelines, children with neuro-psychological alterations and drug-resistant epilepsy need to have CMA as the first diagnostic step, and all subjects for whom a 14q terminal deletion is identified should also have a standard karyotype to assess for the presence of a ring. Another PAO is the British Ring 20 research. This organization presents real-life stories from patients and supports patient-led approaches to assess the role of ketogenic dietary therapy in reducing seizure frequency and preserve cognition for affected patients.^91^^,^^123^ UNIQUE (https://rarechromo.org/), an overarching family support group for all chromosomal aberrations, is an internationally active group that is in contact with many families of RC carriers. Table 2 lists web resources and PAOs providing RC-related information and patient pilots.

**Table 2:** Web resources for constitutional ring chromosomes

Organization	Link
All ring chromosomes	
A Human Ring Chromosome Registry	http://yybio.tech/hrc/
Small supernumerary marker chromosomes	http://cs-tl.de/DB/CA/sSMC/0-Start.html
UNIQUE	https://rarechromo.org/here e.g. patient information sheets for RC 9, RC 12, RC 14, R 18, RC 22
Chromosome Disorder Outreach (CDO)	https://chromodisorder.org/
Orphanet	https://www.orpha.net provides basic information on several ring chromosomes
NORD	https://rarediseases.org provides basic information on several ring chromosomes
Ring chromosome 14	
Ring 14 International	http://www.ring14.org/eng/
Ring14 Clinical Database	http://www.ring14.org/questionario/crm/
Biobank collection of Ring14 biosamples	Telethon Network of Genetic Biobanks (http://www.ring14.org/eng/341/biobanking-project/)
Ring chromosome 18	
Chromosome 18 Registry & Research Society	http://www.chromosome18.org
Facebook group for Ring 18 in Italian	www.facebook.com/groups/325784750908122
Ring chromosome 20	
Ring 20 Research and Support UK	https://ring20researchsupport.co.uk/
Ring chromosome 22	
Chromosome 22 Central	http://www.c22c.org
Facebook group for Ring 22	https://www.facebook.com/pages/Ring-Chromosome-22/118205524927128
Yahoo group for Ring 22	https://groups.yahoo.com/neo/groups/ring22/info

### Lessons from iPSCs of RCs

Cell lines from patients with RCs have been preserved in cell repositories for research purposes.^124^^,^^125^ Reprograming these patient-derived cell lines to iPSCs offers unprecedented opportunities of *in vitro* cellular models for studies of human development, regenerative medicine, drug screening, and cell therapy.^126^ The first attempts to generate iPSCs from fibroblasts with an RC 17 for Miller-Dieker syndrome and an RC 13 discovered the unexpected disappearance of RCs through a monosomy compensatory UPD mechanism. This cell-autonomous correction involved first the loss of the RC and then the duplication of the normal chromosome within five to ten cell culture passages; the correction ratio varied from different iPSC clones. Remarkably, no RCs 17 and 13 were found in metaphase iPSCs, suggesting that such cells may be terminal and non-dividing in the pluripotent state.^51^ This cell-autonomous correction was proposed as a potentially attractive therapeutic approach for large-scale chromosomal aberrations, named as “chromosome therapy.”^52^ Genetic editing methods for circulation of genes and chromosome using CRISP-Cas9 have been developed.^127^ This genetic editing strategy could also be used to reduce trisomy to disomy by induced ring loss and to correct pathogenic copy-number variation (CNVs) or large aberration by compensatory UPD. Limitations in this chromosome therapy concept include validity and efficacy of the technical procedures, the risk of exposing recessive disease or imprinting disorders, and ethical considerations.^128^^,^^129^

Further studies showed that stable iPSC lines with RCs can be generated, at least for some chromosomes and for some time or passages.^125^^,^^130^, ^131^, ^132^ Marked variability in the mitotic stability of RCs in iPSCs, including instability between isogenic lines, was found.^133^ RCs 8 and 17 appear to be less able to be maintained in the pluripotent state, while RCs 21 and 22 were found most stable in iPSCs. These observations suggest that the smaller the RC size, the more stable it is in iPSCs. In iPSC lines obtained from fibroblasts of the foreskin of a 26-year-old man, two lines had a normal karyotype 46,XY, and another two lines had RC 22 in mosaic state with different types of mosaicism.^134^ Detection of RCs in iPSCs with presumably normal karyotype may be a consequence of low-level mosaicism, which preexisted in the initial tissues but was previously undetected by conventional cytogenetic methods. However, the appearance of RC 22 in two iPSC lines from one person may also be a consequence of the structural peculiarities of the chromosome predisposing to a ring formation.

Since iPSCs simulate early stages of embryo development and are somewhat similar to embryonic stem cells (ESCs), the study of chromosomal instability in iPSCs can help elucidate the origins of RC mosaicism. Chromosome mosaicism is a relatively common finding in *in vitro* fertilization-derived human embryos. Trisomy rescue and monosomy compensatory and resultant UPD have been documented in the prenatal findings of fetoplacental discrepancy and confined placental mosaicism. Self-correction of chromosomal abnormalities in human preimplantation embryos and ESCs has been explained by increased death and decreased division of aneuploid cells or allocation of the aneuploidy in the trophectoderm.^135^^,^^136^ In a small case series, intrauterine transfer of mosaic aneuploid blastocysts developed into healthy euploid newborns.^137^ However, if compensatory UPD is truly a cell-autonomous process, cases with RCs will show self-corrected cells with normal disomic patterns for the involved chromosome. Nevertheless, *in vivo* examples of RC rescue via monosomy appearance followed by chromosome duplication, resulting in compensatory UPD, were reported in patients with an RC 21 and an RC 8.^71^^,^^72^ Of the 95 Chinese patients with an autosome ring, only nine patients were noted with normal cells, and there was no further study to determine a true mosaicism or a compensatory UPD.^57^ Clinical cytogenetic results did not observe a large-scale *in vivo* self-correction. Cellular reprogramming for iPSC may be a necessary step to trigger compensatory UPD. Further study to understand the mechanisms of RC loss and compensatory UPD is needed for practical chromosome therapy.

### sSRCs)

Carriers of sSRCs constitute a subgroup of patients with sSMCs; published patients with sSMC are already summarized in an online database (Table 2). The database included 809 patients of sSRCs: 65 of them are reported in patients with multiple sSMCs; 45 are in connection with a McClintock mechanism for the frequent decrease and occasional increase in size of the rings or for their loss through cell cycles;^2^ 162 are derived from an X chromosome and 79 from a Y chromosome (i.e., they are seen in mosaic Turner syndrome cases as mos 45,X/46,X,+r); and the remaining 458 sSRCs are derived from each possible human chromosome. All of the patients with sSRC but one are derived from a single chromosome; the excepted one unpublished patient listed in the database as 11-Uc-1 has an r(11)t(11; 20) (:11p11.1→11q12.1:20q13.1?2→q13.32:). Like in larger RCs also in sSRCs, there is increasing evidence that in a certain subset of patients, these sSMCs might not consist of simple continuous stretches of pericentric DNA. Instead, some seem to be derived from chromothripsis events and thus lead to so-called discontinuous sSRCs like was recently reported for sSRCs 10 and 19.^138^^,^^139^

Interestingly, telomeres can be present or absent in sSMCs and sSRCs.^61^ Similar stability in mitosis was noted for centric minute or ring-shaped sSMCs. However, at least in cell culture, these centric minute or ring-shaped sSMCs are far less stable than inverted duplication-shaped sSMCs.^140^ Overall, similarities and differences of sSRCs and RCs are not well established and studied in the literature. It is obvious that sSRCs are more likely to lead to small copy-number gains rather than copy-number loss (apart from rare cases with McClintock mechanism formation) and are more likely to go together with mosaic trisomy rather than mosaic monosomy.^107^ Still, similarities of sSRCs and RCs concerning stability in long-term culture have been reported.^51^^,^^140^ Similar mechanisms of formation have been reported as well.^61^

## Future directions

Over the past 60 years, the advance of genetic and genomic technologies has enabled a comprehensive analysis of human RCs. More recent case reports have shown a high percentage of RCs with simple or complex genomic rearrangements and imbalances.^57^^,^^50^ However, the under-reporting of near-normal or mild phenotypes from complete and stable RCs and insufficient analysis on mitotic behavior and genomic alterations of RCs could introduce bias and skew the clinical and cytogenomic heterogeneity for RCs.^102^^,^^67^ Current understanding from reported patients of RCs strongly recommends a comprehensive cytogenomic analysis on the genomic structures and dynamic mosaicism. This cytogenomic analysis could be further complicated by the fact that many cell types and tissues are affected, which are often difficult to access. Better practice in diagnostic and research analyses on RC cases could contribute to a better understanding of the mechanisms governing RC formation and mitotic behavior, more accurate clinic-cytogenomic correlations, and evidence-based clinical treatment and management for patients.

In 2021, we launched an international consortium for human RCs (ICHRC) with the goals of (1) developing laboratory standards and guidelines for analyzing RCs, (2) reanalyzing, reviewing, and registering RC cases into the Human Ring Chromosome Registry as an online database, and (3) performing further genomic characterization and functional analysis of RC structure and behavior. There is a dedicated working group for each RC comprised of clinical and molecular cytogeneticists, clinical geneticists, and genetic researchers. Systematic evidence reviews are performed by each working group following the evidence-based practice guideline proposed by the American College of Medical Genetics and Genomics (ACMG) (https://www.acmgfoundation.org/PDFLibrary/ACMG_Protocol_Manual_for_EB_Guidelines_with_2020_Link%20(1).pdf). Working together with the ACMG laboratory quality assurance committee, laboratory standards and guidelines for human RCs will include technical details of karyotyping, FISH, CMA, and WGS for patients with an RC, diagnostic definitions of complete versus incomplete and stable versus unstable RCs, clinical interpretation for associated phenotypes, and recommendations for follow-up parental studies and clinical management. A program on acquired RCs in various tumors is also considered.

**Figure 3:**
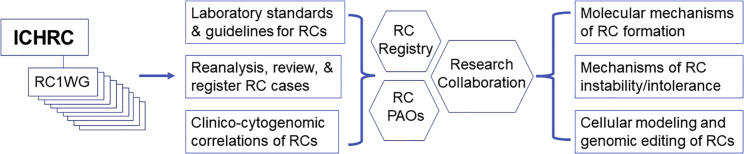
A workflow for organized efforts by ICHRC to provide comprehensive cytogenomic diagnosis, resources for evidence-based interpretation and clinical management, and opportunities for collaborative research

Criteria for reanalyzing, reviewing, and registering patients with an RC into the registry include completed diagnostic cytogenomic analysis, and detailed clinical records. Approximately 80% of patients of RCs reported in the literature lacked the genomic analysis for copy-number alterations in the RCs. The human RC registry plans to register the 20% of RCs with clear cytogenomic and clinical characterization. The ICHRC will provide technical support to reanalyze published RC cases by CMA and WGS. The working groups are aimed to review, curate, and register over 1,000 patients in a period of 5 years for full representation of all autosomal and sex chromosome RCs. As shown in Figure 3, these organized efforts by ICHRC and related resources will enable (1) future evidence-based counseling and clinical management for patients with an RC, (2) the characterization of genomic structure of various RCs for underlying molecular mechanisms of RC formation, (3) the definition of genetic and genomic factors affecting RC instability and intolerance, and (4) the provision of a patient-derived resource for collaborative genetic research.

## Supplemental information

Document S1. Tables S1 and S2

Document S2. Article plus supplemental information
