# Supplementary material for: The past, present, and future for constitutional ring chromosomes: A report of the international consortium for human ring chromosomes
Source: HGG Adv. 2022 Sep 10;3(4):100139. doi: 10.1016/j.xhgg.2022.100139 (PMC9519620; doi:10.1016/j.xhgg.2022.100139)
Supplement: Document S1. Tables S1 and S2 [file mmc1.pdf]

HGGA, Volume *3*

Supplemental information

The past, present, and future for constitutional ring chromosomes: A report of the international consortium for human ring chromosomes

Peining Li, Barbara Dupont, Qiping Hu, Marco Crimi, Yiping Shen, Igor Lebedev, and Thomas Liehr

Supplemental Information

The Past, Present, and Future for Constitutional Ring Chromosomes: A Report of the International Consortium for Human Ring Chromosomes (ICHRC)

Peining Li, Barbara Dupont, Qiping Hu, Marco Crimi, Yiping Shen, Igor Lebedev, Thomas Liehr

Supplementary Tables

Table S1. List of Case Reports of Ring Chromosomes in PubMed 1962-2022

Table S2: Relative Frequency of Constitutional Ring Chromosomes (RCs) and Cytogenomic Findings in PubMed 1962-2022

Table S1. List of Case Reports of Ring Chromosomes in PubMed 1962-2022

Case Count	Case Report	Age Onset or Diagnosis	Gender	Inheritance	Nature History	Karyotype	PMID	Title	Authors	Citation	Publication Year
1	RC1-1	3 m	F	na		46,XX,r(1)[71]/46,XX[7]	14215563	RING-1 CHROMOSOME AND MICROCEPHALIC DWARFISM	GORDON RR, COOKE P.	Lancet. 1964 Dec 5;2(7371):1212-3. doi: 10.1016/s0140-6736(64)91045-	1964
1	RC1-2	8 m	F	na		46,XX,r(1)	6054759	Ring 1 chromosome and dwarfism--a possible syndrome	Wolf CB, Peterson JA, LoGrippo GA, Weiss L.	J Pediatr. 1967 Nov;71(5):719-22. doi: 10.1016/s0022-3476(67)80211-	1967
1	RC1-3	6 m	F	na	d. 10 y	BL: 46,XX,r(1)<br>BM: 47,XX,der(1),+mar	4125054	Ring-1 chromosome, microcephalic dwarfism, and acute myeloid leukemia	Bobrow M, Emerson PM, Spriggs AI, Ellis HL.	Am J Dis Child. 1973 Aug;126(2):257-60. doi:	1973
1	RC1-4	2 m	F	de novo		BL: 46,XX,r(1)[80]/46,XX[10]/r-var[10]<br>SF: 46,XX,r(1)[28]/46,XX[2]	679524	Apparently non-deleted ring-1 chromosome and extreme growth failure in a mentally retarded girl	Kjessler B, Gustavson KH, Wigertz A.	Clin Genet. 1978 Jul;14(1):8-15. doi: 10.1111/j.1399-	1978
1	RC1-5	nb	F	de novo		46,XX,r(1)	6502659	Ring chromosome 1 associated with radial ray defect	Gardner RJ, Grindley RM, Chewings WE, Clarkson JE.	J Med Genet. 1984 Oct;21(5):400. doi: 10.1136/jmg.21.5.400.	1984
1	RC1-6	nb	F	nd		BL: 46,XX,r(1)(p36.3q44)/r-var[2/80]<br>SF: 45,XX,psu dic der(16;1)t(16;1;20)(p11.2;q41p12;p11.2),der(20)t(16;1;20)[45]/46,XX,r(1)(p36.3q44)[5]	10826627	Ring chromosome 1 in a newborn	Cutenese C, Mullett M, Hummel M, Wenger SL.	Clin Dysmorphol. 2000 Apr;9(2):131-3. doi: 10.1097/00019605-200009020-00012.	2000
1	RC1-7	3 y	M	de novo		46,XY,r(1)(p36.3q44)[10]/45,XY-1[5]	27099748	A patient with constitutional ring 1 chromosome characterized by SNP array CGH	Saliganan S, Lee J, Wei S.	Clin Case Rep. 2016 Mar 21;4(4):442-8. doi:	2016
**7**	**7**										
1	RC2-1		F			46,XX/46,XX,r(2)(p25q37)	315192	46,XX/46,XX,r (2)(p25q37) mosaicism: clinical and cytogenetic studies	Sutherland GR, Carter RF.	Ann Genet. 1978 Sep;21(3):164-7.	1978
1	RC2-2 case 1	1.25 y	M	de novo		46,XY,r(2)	457140	Three cases of ring chromosome 2, one derived from a paternal 2/6 translocation	Maraschio P, Danesino C, Garau A, Saputo V, Vigi V, Volpato S.	Hum Genet. 1979 Apr 27;48(2):157-67. doi: 10.1007/BF00286899.	1979
1	RC2-2 case 2	10 m	F	r(2;6) der t(2;6)pat		Proband: 46,XX,r(2;6)<br>Pat: 46,XY,t(2;6)(p15;q15)	457140				
1	RC2-2 case 3	6 m	M	de novo	d. 10.5 m	46,XY,r(2)	457140				
1	RC2-3	1.5 y	F	de novo		46,XX,r(2)[311]/46,XX[64]/r-var[4]	7468662	Ring chromosome 2 in a child with growth failure and few congenital abnormalities	Vigfusson NV, Kapstafer KJ, Lloyd MA.	Am J Med Genet. 1980;7(3):383-9. doi: 10.1002/ajmg.1320070321.	1980
1	RC2-4	1.5 y	F	de novo	> 35 y	46,XX,r(2)(p25q37)196]/r-var[29]	6977305	The cytogenetic and clinical implications of a ring chromosome 2	Cote GB, Katsantoni A, Deligeorgis D.	Ann Genet. 1981;24(4):231-5.	1981
0	RC2-4 (follow-up)						25997743	35-Year Follow-Up of a Case of Ring Chromosome 2: Array-CGH Analysis and Literature Review of the Ring Syndrome	Sarri C, Douzgou S, Kontos H, Anagnostopoulou K, Tümer Z, Grigoriadou M, Petersen MB, Kokotas H, Merou K, Pandelia E, Giouroukou E,	Cytogenet Genome Res. 2015;145(1):6-13. doi: 10.1159/000382046. Epub 2015 May 14.	2015
1	RC2-5	4 y	F	de novo		46,XX,r(2)(p25q37)	7076252	Ring chromosome 2: clinical, chromosomal, and biochemical aspects	Jansen M, Beemer FA, van der Heiden C, Van Hemel JO, Van den Brande JL.	Hum Genet. 1982;60(1):91-5. doi: 10.1007/BF00281274.	1982
1	RC2-6	nb	M			46,XY,r(2)(p25q37.3)[0.65]/45,XY,-2[0.27]/46,XY[0.02]	10406663	Ring 2 chromosome: ten-year follow-up report	Lacassie Y, Arriaza MI, Vargas A, La Motta I.	Am J Med Genet. 1999 Jul 16;85(2):117-22. doi: 10.1002/(sici)1096-	1999
1	RC2-7	nb	F	de novo		46,XX,r(2)(p25q37)[79]/45,XX,-2[4]/r-var[17]	11546833	A case of ring chromosome 2 with growth retardation, mild dysmorphism, and microdeletion of 2p detected using FISH	Dee SL, Clark AT, Willatt LR, Yates JR.	J Med Genet. 2001 Sep;38(9):E32. doi: 10.1136/jmg.38.9.e32.	2001
1	RC2-8		F			46,XX,r(2)(p25.3q37.3).ish r(2)(2pter+,2qtel+)	15844781	"Ring syndrome" involving chromosome 2 confirmed by FISH analysis using chromosome-specific subtelomeric probes	Kosho T, Matsushima K, Sahashi T, Mitsui N, Fukushima Y, Sobajima H, Ohashi H.	Genet Couns. 2005;16(1):65-70.	2005
1	RC2-9	nb	M			46,XY,r(2)(p25q37)[13]/45,XY,[illegible]2[4]/47,XY,r(2)(p25q37)x2[2]/46,XY,dic r(2;2)(p25q37;p25q37)[1]	15580637	A patient with a ring chromosome 2 and microdeletion of 2q detected using FISH: Further support for "ring chromosome 2 syndrome"	Alkuraya FS, Kimonis VE, Holt L, Murata-Collins JL.	Am J Med Genet A. 2005 Feb 1;132A(4):447-9. doi: 10.1002/ajmg.a.30437.	2005
1	RC2-10	pn, 23 gwk	M	de novo	top	46,XY,r(2)(p25.3q37.3)	23403238	Prenatal diagnosis of ring chromosome 2 with lissencephaly and 2p25.3 and 2q37.3 microdeletions detected using array comparative genomic hybridization	Chen CP, Lin CJ, Chang TY, Chern SR, Wu PS, Chen YT, Su JW, Lee CC, Chen LF, Wang W.	Gene. 2013 Apr 25;519(1):164-8. doi: 10.1016/j.gene.2013.01.055. Epub 2013 Feb 9.	2013
1	RC2-11	10 m	M	de novo		46,XY,r(2)(p25q37)[17]/47,XY,r(2),+r(2)[3]/46,XY[1]	23895799	Ring 2 chromosome associated with failure to thrive, microcephaly and dysmorphic facial features	López-Uriarte A, Quintero-Rivera F, de la Fuente Cortez B, Puente VG, Campos Mdel R, de Villarreal LE.	Gene. 2013 Oct 15;529(1):65-8. doi: 10.1016/j.gene.2013.06.056. Epub 2013 Jul 27.	2013
1	RC2-12	nb	M	de novo		46,XY,r(2)(p25.3q37.3)[92]/r-var[8]	25774222	Clinico-radiological and molecular characterization of a child with ring chromosome 2 presenting growth failure, microcephaly, kidney and brain malformations	Severino M, Accogli A, Gimelli G, Rossi A, Kotzeva S, Di Rocco M, Ronchetto P, Cuoco C, Tassano E.	Mol Cytogenet. 2015 Mar 5;8:17. doi: 10.1186/s13039-015-0121-z. eCollection 2015.	2015
**14**	**13**										
1	RC3-1	nb	M	de novo		46,XY,r(3)	669717	Ring chromosome 3 in a retarded boy	Witkowski R, Ullrich E, Piede U.	Hum Genet. 1978 Jun 27;42(3):345-8. doi: 10.1007/BF00291318.	1978

1	RC3-2	2 m	M	nl mat		46,XY,r(3)(p26q29)[0.92BL/0.90SF]	7154048	The phenotype of ring chromosome 3	Wilson GN, Pooley J, Parker J.	J Med Genet. 1982 Dec;19(6):471-3. doi: 10.1136/jmg.19.6.471.	1982
1	RC3-3	10 m	F	de novo		46,XX,r(3)(p26q29)[223]/46,XX[10]/r-var[55]	6513151	A case of ring chromosome 3, 46,XX,-3,+r(3)(p26q29)	Kitatani M, Takahashi H, Yasuda J, Chen CC, Ida F, Shike S.	Jinrui Idengaku Zasshi. 1984 Jun;29(2):157-62. doi: 10.1007/BF01873537.	1984
1	RC3-4	4 m	M	de novo		46,XY,r(3)	2359108	Cornelia de Lange syndrome with ring chromosome 3	Lakshminarayana P, Nallasivam P.	J Med Genet. 1990 Jun;27(6):405-6. doi: 10.1136/jmg.27.6.405.	1990
0	RC3-4						2002489	Cornelia de Lange syndrome with ring chromosome 3	Wilson GN.	J Med Genet. 1991 Feb;28(2):143. doi: 10.1136/jmg.28.2.143.	1991
1	RC3-5	46 y	M		> 46 y	46,XX,r(3)(p26q29)[0.93]	1952790	[Ring chromosome 3 in a mentally retarded adult dwarf]	Teyssier M, Piperno D, Charrin C.	Ann Genet. 1991;34(1):33-6.	1991
1	RC3-6	18 y	F	de novo		46,XX,r(3)(p26q29)	1920370	De novo ring chromosome 3: a new case with a mild phenotype	McKinley M, Colley A, Sinclair P, Donnai D, Andrews T.	J Med Genet. 1991 Aug;28(8):536-8. doi: 10.1136/jmg.28.8.536.	1991
1	RC3-7	4 y	F		> 23 y	47,XX,-3,+r(3)(p21.3q25),+rea(3)[76]/46,XX[16]/r-var[6]	8786088	A novel mechanism for the origin of supernumerary marker chromosomes	Maraschio P, Tupler R, Rossi E, Barbierato L, Uccellatore F, Rocchi M, Zuffardi O, Fraccaro M.	Hum Genet. 1996 Mar;97(3):382-6. doi: 10.1007/BF02185778.	1996
1	RC3-8		F	de novo		46,XX,r(3)(p26q29)	11491310	De novo ring chromosome 3 in a girl with hypoplastic thumb and coloboma of iris	Barajas-Barajas LO, Velarde-Félix S, Elizarrarás-Rivas J, Hernández-Zaragoza	Genet Couns. 2001;12(2):151-6.	2001
1	RC3-9	8 m	M			46,XY,r(3)(p26.1q29)	21849783	Clinical, cytogenetic and molecular study in a case of r(3) with 3p deletion and review of the literature	Guilherme RS, Bragagnolo S, Pellegrino R, Christofolini DM, Takeno SS, Carvolheira GM, Kulikowski LD,	Cytogenet Genome Res. 2011;134(4):325-30. doi: 10.1159/000329478. Epub 2011	2011
1	RC3-10	4 m	F	de novo		46,XX,r(3)(p26q29)[90]/47,XX,+2r(3)[5]/46,XX,der(3)[5]	27077748	Chromosome r(3)(p25.3q29) in a Patient with Developmental Delay and Congenital Heart Defects: A Case Report and a Brief Literature	Zhang K, Song F, Zhang D, Liu Y, Zhang H, Wang Y, Dong R, Zhang Y, Liu Y, Gai Z.	Cytogenet Genome Res. 2016;148(1):6-13. doi: 10.1159/000445273. Epub 2016 Apr	2016
0	RC3-10						27984612	[Molecular cytogenetic analysis of a case with ring chromosome 3 syndrome]	Zhang K, Song F, Zhang D, Zhang H, Wang Y, Dong R, Zhang Y, Liu Y, Gai Z.	Zhonghua Yi Xue Yi Chuan Xue Za Zhi. 2016 Dec 10;33(6):816-819.	2016
1	RC3-11						32090537	A rare case of ring chromosome 3 syndrome	Huang T, Zhu L, Zhang SF, Hu XY, Cheng P, Luan SQ, Chen GH.	J Biol Regul Homeost Agents. 2020 Feb 24;34(1). doi: 10.23812/19-277-	2020
11	12										
1	RC4-1	nb	M	de novo	d. 4 w	46,XY,r(4)[165/200]	5801472	Congenital malformations associated with a ring 4 chromosome	Carter R, Baker E, Hayman D.	J Med Genet. 1969 Jun;6(2):224-7. doi: 10.1136/jmg.6.2.224.	1969
1	RC4-2	9 m	F	de novo		46,XX,r(4)	5149537	A ring-4 chromosome in a patient with normal intelligence and short stature	Surana RB, Bailey JD, Conen PE.	J Med Genet. 1971 Dec;8(4):517-21. doi: 10.1136/jmg.8.4.517.	1971
1	RC4-3	9 m	F	de novo		46,XX/46,XX,r(4)[0.09/0.58/0.64/0.72]age2/3/5/9	4411581	A child with a ring-4 chromosome (46,XX-46,XX,r 4)	Parker CE, Alfi OS, Derencsenyi A, Mavalwala J, Donnell G.	Am J Dis Child. 1974 Sep;128(3):371-4. doi: 10.1001/archpedi.1974.02110280101015.	1974
1	RC4-4	8 y	M	de novo		46,XY,r(4)[0.90]	1150264	Derivative chromosomal structures from a ring chromsome 4	Niss R, Passarge E.	Humangenetik. 1975 May 26;28(1):9-23. doi: 10.1007/BF00272478.	1975
0	RC4-4	12 y					872629	Sister chromatid exchanges in a ring chromosome 4	Bartram CR.	Cytogenet Cell Genet. 1977;18(4):238-41. doi: 10.1159/000130766.	1977
1	RC4-6	1.5 y	M	de novo		46.XY,r(4)(p16q35)	881718	Ring chromosome 4	McDermott A, Voyce MA, Romain D.	J Med Genet. 1977 Jun;14(3):228-32. doi: 10.1136/jmg.14.3.228.	1977
1	RC4-7	nb	M	de novo	d. 1 wk	46,XY,r(4)(p15q35)[100]/r-var[23]	881198	Ring chromosome 4 and Wolf syndrome	Pérez-Castillo A, Abrisqueta JA.	Hum Genet. 1977 Jun 10;37(1):87-91. doi: 10.1007/BF00293777.	1977
1	RC4-8	nb	F	de novo	d. 4 d	46,XX,r(4)(p16q35)[87]/r-var[13]	711237	Partial deletion of 4p16 band in a ring chromosome and Wolf Syndrome	del Mazo J, Abrisqueta JA, Pérez-Castillo A, Aller V, Lucas MA, de Torres ML, Martín MJ.	Hum Genet. 1978 Oct 19;44(1):105-8. doi: 10.1007/BF00283580.	1978
1	RC4-9	nb	F		d. 6 y	46,XX,r(4)	7205435	Neurological and neuropathological findings in ring chromosome 4	Young RS, Zalneraitis EL.	J Med Genet. 1980 Dec;17(6):487-90. doi: 10.1136/jmg.17.6.487.	1980
1	RC4-10	2 m	M	de novo		46,XY,r(4)(p16q35)[0.77]	7270516	Ring 4 chromosome with terminal p and q deletions	Finley WH, Finley SC, Chonmaitree T, Koors JE, Chandler WC.	Am J Dis Child. 1981 Aug;135(8):729-31. doi: 10.1001/archpedi.1981.02130320043015.	1981
1	RC4-11	1.5 y	F			46,XX,r(4)	4041283	Ring chromosome 4: Wolf syndrome and unspecific developmental anomalies	Kosztolányi G.	Acta Paediatr Hung. 1985;26(2):157-65.	1985
0	RC4-11						3674110	Decreased cell viability of fibroblasts from two patients with a ring chromosome: an in vitro reflection of growth failure?	Kosztolányi G.	Am J Med Genet. 1987 Sep;28(1):181-4. doi: 10.1002/ajmg.1320280125.	1987
1	RC4-12	12 y	M			46,XY,r(4)(p16q35)	4046483	Ring chromosome 4 : 46,XY, r(4) (p16q35) in a boy	Gutkowska A, Krajewska-Walasek M, Wiśniewski L.	Klin Padiatr. 1985 Jul-Aug;197(4):294-6. doi: 10.1055/s-2008-1033986.	1985

1	RC4-13	11 m	M			46,XY,r(4)(p16q35)	3671134	[Ring chromosome 4 in twins]	Giuffrè L, Cammarata M, Corsello G, Benigno V, Graziano L, Roccella F,	Pediatr Med Chir. 1987 May-Jun;9(3):349-50.	1987
1	RC4-14	nb	M			46,XY/46,XY,r(4)	3261147	Ring chromosome 4 mosaicism and Potter sequence	Fryns JP, Kleczkowska A, Jaeken J, Van den Berghe H.	Ann Genet. 1988;31(2):120-2.	1988
1	RC4-16	nb	F	de novo		46,XX,r(4)(p16q35)[81]/45,XX,-4[12]/r-var[2]	2240048	Ring chromosome 4 in a child with duodenal atresia	Halal F, Vekemans M.	Am J Med Genet. 1990 Sep;37(1):79-82. doi: 10.1002/ajmg.1320370118.	1990
1	RC4-17	8 y	M	de novo		46,XY,r(4)(p16q35)[72]/45,XY,-4[3]/r-var[25]	2015696	Ring chromosome 4 in a child with mild dysmorphic signs	Freyberger G, Wamsler C, Schmid M.	Clin Genet. 1991 Feb;39(2):151-5. doi: 10.1111/j.1399-0004.1991.tb03003.x.	1991
1	RC4-18	nb	F	de novo		46,XX[0.84]/46,XX,r(4)(p16q22.3)[0.12]/47,XX,r(4),+del(4)(q22.3)[0.04]	8933346	Amelia, dextrocardia, asplenia, and congenital short bowel in deleted ring chromosome 4	Hou JW, Wang TR.	J Med Genet. 1996 Oct;33(10):879-81. doi: 10.1136/jmg.33.10.879.	1996
1	RC4-19	nb	M	de novo	> 26 y	46,XY,r(4)(p16.3q35)[9]/46,XY[11]	9332654	Ring chromosome 4 mosaicism coincidence of oligomeganephronia and signs of Seckel syndrome	Anderson CE, Wallerstein R, Zamerowski ST, Witzleben C, Hoyer JR,	Am J Med Genet. 1997 Oct 31;72(3):281-5.	1997
1	RC4-20	pn: 17 gwks	F		top	46,XX,r(4)[0.44]/45,XX,-4[0.56]	12011164	Partial deletion of 4p and 4q in a fetus with ring chromosome 4: phenotype and molecular mapping of the breakpoints	Kocks A, Endele S, Heller R, Schröder B, Schäfer HJ, Städtler C, Makrigeorgi-Butera M, Winterpacht A.	J Med Genet. 2002 May;39(5):E23. doi: 10.1136/jmg.39.5.e23.	2002
1	RC4-21	27 y	F	de novo	> 27 y	46,XX,r(4)(p16q35)[160]/45,XX,-4[16]/r-var[17]/46,XX[21]	16103661	Molecular cytogenetic characterization of ring chromosome 4 in a female having a chromosomally normal child	Lee MH, Park SY, Kim YM, Kim JM, Yoo KJ, Lee HH, Ryu HM.	Cytogenet Genome Res. 2005;111(2):175-8. doi: 10.1159/000086389.	2005
1	RC4-22	2.5 y	F	de novo	> 26 y	46,XX,r(4)(p16q35)	16082703	Ring chromosome 4 in a patient with early onset type 2 diabetes, deafness, and developmental	Blackett PR, Li S, Mulvihill JJ.	Am J Med Genet A. 2005 Aug 30;137(2):213-6. doi:	2005
1	RC4-23						16719275	Wolf Hirshhorn syndrome in a case of ring chromosome 4: phenotype and molecular cytogenetic findings	Laleye A, Alao MJ, Adjagba M, Hans C, Delneste D, Gnamey DK, Ayivi B, Darboux RB.	Genet Couns. 2006;17(1):35-40.	2006
1	RC4-24	nb	M	de novo		46,XY,r(4)(p16.3q35)	16470698	Ring chromosome 4 and Wolf-Hirschhorn syndrome (WHS) in a child with multiple	Balci S, Engiz O, Aktaş D, Vargel I, Beksaç MS, Mrasek K, Vermeesch J,	Am J Med Genet A. 2006 Mar 15;140(6):628-32. doi:	2006
1	RC4-25	pn: 26 gwks	M	de novo	stillbirth, 29 gwks	46,XY,r(4)(p15.2q35.2)[11]/45,XY,-4[12]	17471607	Prenatal diagnosis of mosaic ring chromosome 4	Chen CP, Hsu CY, Tzen CY, Lee CC, Chen WL, Chen LF, Wang W.	Prenat Diagn. 2007 May;27(5):485-7. doi: 10.1002/pd.1717.	2007
1	RC4-26	nb	M	de novo		46,XY,r(4)(4p16.3q35)	17696124	Two unique patients with novel microdeletions in 4p16.3 that exclude the WHS critical regions: implications for critical region designation	South ST, Bleyl SB, Carey JC.	Am J Med Genet A. 2007 Sep 15;143A(18):2137-42. doi: 10.1002/ajmg.a.31900.	2007
1	RC4-27	10 m	M	de novo		46,XY,r(4)(p16q35)[84]/45,XY,-4[9]/r-var[7]	19262083	[A case of mosaic ring chromosome 4 with subtelomeric 4p deletion]	Kim JH, Oh PS, Na HY, Kim SH, Cho HC.	Korean J Lab Med. 2009 Feb;29(1):77-81. doi: 10.3343/kjlm.2009.29.1.77.	2009
1	RC4-28	1.5 y	M	de novo		46,XY,r(4)[83]/45,XY,-4[6]/r-var[11]	19921639	Characterization of double ring chromosome 4 mosaicism associated with bilateral hip dislocation, cortical dysgenesis, and epilepsy	Soysal Y, Balci S, Hekimler K, Liehr T, Ewers E, Schoumans J, Bui TH, Içduygu FM, Kosyakova N, Imirzalioğlu N.	Am J Med Genet A. 2009 Dec;149A(12):2782-7. doi: 10.1002/ajmg.a.33069.	2009
1	RC4-29						22029176	Mosaic ring chromosome 4 in a child with mild dysmorphisms, congenital heart defects and developmental delay	Chen CP, Lin SP, Su YN, Chern SR, Tsai EJ, Wu PC, Lee CC, Wang W.	Genet Couns. 2011;22(3):321-6.	2011
1	RC4-30	pn	F	de novo	denial top	46,XX,r(4)	24455347	Prenatal diagnosis of 4p and 4q subtelomeric microdeletion in de novo ring chromosome 4	Akbas H, Cine N, Erdemoglu M, Atay AE, Simsek S, Turkyilmaz A, Fidanboy	Case Rep Obstet Gynecol. 2013;2013:248050. doi:	2013
1	RC4-31	23 y	M	de novo	> 23 y	46,XY,r(4)(p16.3q35.2)[31]/45,XY,-4[3]	25057292	Meiotic prophase I defects in an oligospermic man with Wolf-Hirschhorn syndrome with ring chromosome 4	Yao Q, Wang L, Yao B, Gao H, Li W, Xia X, Shi Q, Cui Y.	Mol Cytogenet. 2014 Jul 1;7:45. doi: 10.1186/1755-8166-7-45. eCollection 2014.	2014
1	RC4-32	nb	F	de novo	d.10 wks, cardiac failure	46,XX,r(4)(p15.3q35)	27610251	Ring Chromosome 4 in a Child with Multiple Congenital Abnormalities: A Case Report and Review of the Literature	Paththinige CS, Sirisena ND, Kariyawasam UG, Saman Kumara LP, Dissanayake VH.	Case Rep Genet. 2016;2016:4645716. doi: 10.1155/2016/4645716. Epub 2016 Aug 16.	2016
1	RC4-33	2 y	M	de novo		46,XY,r(4)(p16.3q35.2)[42]/46,XY[11]/r-var[6]	28127864	Continuing role for classical cytogenetics: Case report of a boy with ring syndrome caused by complete ring chromosome 4 and review of literature	Burgemeister AL, Daumiller E, Dietze-Armana I, Klett C, Freiberg C, Stark W, Lingen M, Centonze I, Rettenberger G, Mehnert K, Zirn B.	Am J Med Genet A. 2017 Mar;173(3):727-732. doi: 10.1002/ajmg.a.38063. Epub 2017 Jan 27.	2017
1	RC4-34						30428788	A Case of Thumb Polydactyly which Ulnar Thumb Has No Active Motion in Ring Chromosome 4	Kishi Y, Ikeda H.	J Hand Surg Asian Pac Vol. 2018 Dec;23(4):566-570. doi: 10.1142/S2424835518720311.	2018
1	RC4-35	10 y	F	de novo		46,XX,r(4)(p16q35)[65]/45,XX,-4[5]/46,XX,dic r(4)[5]/46,XX[16]	32293439	Clinical, cytogenetic, and molecular findings in a patient with ring chromosome 4: case report and literature review	Paz-Y-Miño C, Proaño A, Verdezoto SD, García JL, Hernández-Rivas JM, Leone PE.	BMC Med Genomics. 2019 Nov 21;12(1):167. doi: 10.1186/s12920-019-0614-4.	2019
1	RC4-36	2 y	M			46,XY,r(4)(p16q35)[84]/45,XY,-4[3]/r-var[13]	32761591	[Analysis of clinical and genetic characteristics of a child with ring chromosome 4 syndrome]	Lyu Y, Song F, Zhang K, Gao M, Ma J, Wang D, Wan Y, Liu Y, Gai Z.	Zhonghua Yi Xue Yi Chuan Xue Za Zhi. 2020 Aug 10;37(8):843-846.	2020
**34**	**36**										
1	RC5-1	nb	F	de novo		46,XX,r(5)	4159145	"Cri du Chat" due to a ring-B chromosome	R A Rohde, R Tompkins	Lancet 2(7421):1075-6	1965

1	RC5-2 case 1	1 m	M	de novo		46,XY,r(5)[0.81]	699350	Ring chromosome 5 in two malformed boys with Cri du Chat syndrome	Suerinck E, Noël B, Rethore MO.	Clin Genet. 1978 Sep;14(3):125-9. doi: 10.1111/j.1399-0004.1978.tb02116.x.	1978
1	RC5-2 case 2	10 y	M	de novo		46,XY,r(5)[0.93]	699350				
1	RC5-3						6210897	Ring chromosome 5 with dental anomalies	Kula K, Patil S, Hanson J, Nowak A,	Pediatr Dent. 1981 Dec;3(4):329-	1981
1	RC5-4	2.5 m	M	de novo		46,XY,r(5)	3409543	Ring chromosome 5	Flannery DB, Rogers WG, Byrd JR.	Clin Genet. 1988 Jul;34(1):74-8. doi:	1988
2	RC5-5						2677233	[The cat cry (cri du chat) syndrome: report of a case with review of 10 cases at the National Taiwan University Hospital]	Chuang SM, Wang TR, Jean HH, Lee FY.	Taiwan Yi Xue Hui Za Zhi. 1989 Jun;88(6):635-8, 628-9.	1989
1	RC5-6 case 1	4 y	F	mat		46,XX,r(5)(p15q35)[0.89]	2227937	Investigation of three patients with the "ring syndrome", including familial transmission of ring 5, and estimation of reproductive risks	MacDermot KD, Jack E, Cooke A, Turleau C, Lindenbaum RH, Pearson J, Patel C, Barnes PM, Portch J, Crawfurd	Hum Genet. 1990 Oct;85(5):516-20. doi: 10.1007/BF00194228.	1990
1	RC5-6 case 3	4 m	F	de novo		46,XX,r(5)(p15q35)[0.74]	2227937				
1	RC5-7	2 y	F	de novo		46,XX,r(5)[0.88]	8172236	Ring chromosome 5 associated with severe growth retardation as the sole major physical abnormality	Migliori MV, Cherubini V, Bartolotta E, Pettinari A, Pecora R.	Am J Med Genet. 1994 Jan 1;49(1):108-10. doi: 10.1002/ajmg.1320490121.	1994
1	RC5-8	nb	M	de novo	d. 4.5 m	46,XY,r(5)[183]/45,XY,-5[30]	22193390	Clinical and molecular cytogenetic studies in ring chromosome 5: report of a child with congenital abnormalities	Basinko A, Giovannucci Uzielli ML, Scarselli G, Priolo M, Timpani G, De Braekeleer M.	Eur J Med Genet. 2012 Feb;55(2):112-6. doi: 10.1016/j.ejmg.2011.11.005. Epub 2011 Dec 2.	2012
1	RC5-9	3 y	F	de novo		46,XX,r(5)(p14q35)[80]/45,XX,-5[8]/47,+r(5)[3]	24052730	Ring autosomes: some unexpected findings	Caba L, Rusu C, Plăiaşu 5th, Gug G, Grămescu M, Bujoran C, Ochiană D, Voloşciuc M, Popescu R, Braha E, Pânzaru M, Butnariu L, Sireteanu A, Covic M, Gorduza E.	Balkan J Med Genet. 2012 Dec;15(2):35-46. doi: 10.2478/bjmg-2013-0005.	2012
1	RC5-10	17 y	F			46,XX,r(5)(p14.3q35.3)	32519173	Myelodysplastic syndromes in a pediatric patient with Cri du Chat syndrome with a ring chromosome 5	Nozawa A, Ozeki M, Yasue S, Endo S, Kadowaki T, Ohnishi H, Muramatsu H, Hama A, Takahashi Y, Kojima S, Fukao T.	Int J Hematol. 2020 Nov;112(5):728-733. doi: 10.1007/s12185-020-02909-7. Epub 2020 Jun 9.	2020
**13**	**10**										
1	RC6-1	1 y	F	de novo		BL: 46,XX,r(6)[66]; SF: 46,XX,r(6)[98]	4774541	Developmental abnormalities associated with a ring chromosome 6	Moore CM, Heller RH, Thomas GH.	J Med Genet. 1973 Sep;10(3):299-303. doi: 10.1136/jmg.10.3.299.	1973
1	RC6-2	?	M			46,XY,r(6)/45,XY,-6	4546343	[Ring chromosome 6. Karotype 46, XY, r (6)-45, XY,-6]	Van den Berghe H, Fryns JP, Cassiman JJ, David G.	Ann Genet. 1974 Mar;17(1):29-35.	1974
0	RC6-2 fu						2288465	Ring chromosome 6: twenty years follow-up	Fryns JP, Kleczkowska A, van den	Ann Genet. 1990;33(3):179.	1990
1	RC6-3	1 y	M			46,XY,r(6)[150]/45,XY,-6[15]/r-var[9]	1139788	Mental retardation and congenital malformations associated with a ring chromosome 6	Fried K, Rosenblatt M, Mundel G, Krikler R.	Clin Genet. 1975 Mar;7(3):192-6. doi: 10.1111/j.1399-0004.1975.tb00318.x.	1975
1	RC6-4	nb	M	de novo	d. 6 m	46,XY,r(6)	1204234	Ring chromosome 6 in a malformed boy	Salamanca-Gonez F, Nava S, Armendares S.	Clin Genet. 1975 Nov;8(5):370-5. doi: 10.1111/j.1399-0004.1975.tb01516.x.	1975
1	RC6-5	5 y	F			46,XX,r(6)	305748	[Ring 6-chromosome: a nonspecific clinical picture]	Sele B, Joannard A, Jalbert P, Boucharla	Ann Genet. 1977 Dec;20(4):232-6.	1977
1	RC6-6	4.5 y	M	nd		46,XY,r(6)(p25q26)[48]/46,XY[5]	511129	Ring chromosome 6: case report and review of literature	Kini KR, Van Dyke DL, Weiss L, Logan MS.	Hum Genet. 1979;50(2):145-9. doi: 10.1007/BF00390235.	1979
1	RC6-7	7 y	M	de novo		46,XY,r(6)	517581	Ring chromosome 6 in a child with minimal abnormalities	Carnevale A, Blanco B, Castillo J, del Castillo V, Dominguez D.	Am J Med Genet. 1979;4(3):271-7. doi: 10.1002/ajmg.1320040310.	1979
1	RC6-8	1.5 y	M			46,XY,r(6)	7205202	[Phenotype of a ring-chromosome 6 carrier. Clinical and cytogenetic study]	Cruz-Marin F, Gilgenkrantz S, Gregoire MJ, Beley G, Pierson M.	J Genet Hum. 1980 Dec;28(4):95-104.	1980
1	RC6-9	3 y	F	nd		46,XX,r(6)[0.73}	7091194	Ring chromosome 6: case report and review	Nishi Y, Yoshimura O, Ohama K, Usui T.	Am J Med Genet. 1982 May;12(1):109-14. doi: 10.1002/ajmg.1320120115.	1982
1	RC6-10 case 1	nb	F	de novo	d. 2 wks	46,XX,r(6)[78/106]	6660249	Ring chromosome 6: variability in phenotypic expression	Peeden JN, Scarbrough P, Taysi K, Wilroy RS, Finley S, Luthardt F,	Am J Med Genet. 1983 Dec;16(4):563-73. doi:	1983
1	RC6-10 case 2	6 y	F	de novo		46,XX,r(6)[138/252]	6660249				
1	RC6-10 case 3	2 m	F	de novo		46,XX,r(6)[39/40]	6660249				
1	RC6-10 case 4	1 y	M	de novo		46,XY,r(6)(p25q26)[91/116]	6660249				
1	RC6-11	nb	M	de novo		46,XY,r(6)(p24q26)[43/55]	3777023	Aniridia, congenital glaucoma, and hydrocephalus in a male infant with ring chromosome 6	Levin H, Ritch R, Barathur R, Dunn MW, Teekhasaenee C, Margolis S.	Am J Med Genet. 1986 Oct;25(2):281-7. doi: 10.1002/ajmg.1320250212.	1986
1	RC6-12	1 m	M	de novo		46,XY,r(6)(p24q26)[38]/45,XY,-6[9]/r-var[3]	3544845	Ring chromosome 6: report of a patient and literature review	Chitayat D, Hahm SY, Iqbal MA, Nitowsky HM.	Am J Med Genet. 1987 Jan;26(1):145-51. doi: 10.1002/ajmg.1320260122.	1987
1	RC6-13	1.5 y	F	de novo		46,XX,r(6)[51]/45,XX,-6[49]	3653144	Erroneous diagnosis of fetal alcohol syndrome in a patient with ring chromosome 6	Römke C, Heyne K, Stewens J, Schwinger E.	Eur J Pediatr. 1987 Jul;146(4):443. doi: 10.1007/BF00444963.	1987
1	RC6-14	3 y	F	nd		46,XX,r(6)/45,XX,-6	3314511	Decreased superoxide dismutase-2 activity in a patient with ring chromosome 6	Yoshimitsu K, Nishi Y, Kobayashi Y, Yoshimura O, Ohama K, Oguma N, Usui T.	Am J Med Genet. 1987 Sep;28(1):211-4. doi: 10.1002/ajmg.1320280128.	1987

1	RC6-15	2 y	F			46,XX,r(6)[0.72-0.90 in 11 times]	2333874	Ring chromosome 6: clinical and cytogenetic behaviour	Paz-y-Miño C, Benítez J, Ayuso C, Sánchez-Cascos A.	Am J Med Genet. 1990 Apr;35(4):481-3. doi: 10.1002/ajmg.1320350407.	1990
1	RC6-16	pn	F	CtB	d. 4 m, respiratory arrest	46,XX,r(6)(p25q27)[0.38]/45,XX,-6[0.62]	8559761	Prenatal diagnosis of ring chromosome 6	Dawson AJ, Marles SL, Harman CR, Phillips S, Menticoglou S.	Prenat Diagn. 1995 Sep;15(9):872-4. doi: 10.1002/pd.1970150915.	1995
1	RC6-17	pn	M	mat nl, pat nd	Ctb, > 17 m	45,XY,-6/45,XY,-6,+f/46,XY,r(p25q27)	8905901	Prenatal diagnosis of ring chromosome 6 in a fetus with hydrocephalus	Walker ME, Lynch-Salamon DA, Milatovich A, Saal HM.	Prenat Diagn. 1996 Sep;16(9):857-61. doi: 10.1002/(SICI)1097-0223(199609)16:9<857::AID-PD950>3.0.CO;2-J.	1996
1	RC6-18	pn	M	de novo	Ctb	46,XY,r(6)[0.92]/45,XY,-6[0.05]/46,XY[0.03]	11334612	Microdissection and reverse painting reveals a microdeletion 6(q26qter) in a de novo r(6) chromosome	Birnbacher R, Chudoba I, Pirc-Danoewinata H, König M, Kohlhauser C, Schnedl W, Haas OA.	Ann Genet. 2001 Jan-Mar;44(1):13-8. doi: 10.1016/s0003-3995(00)01033-9.	2001
1	RC6-19	3 y	M	de novo		46,XY,r(6)(p25q27)[17]/45,XY,-6[4]/r-var[6]	11223855	An 11-year-old boy with mosaic ring chromosome 6 and dilated aortic root	Ivanovich JL, Watson MS, Whelan AJ.	Am J Med Genet. 2001 Jan 15;98(2):182-4. doi: 10.1002/1096-	2001
1	RC6-20 case 1	pn	M		top	AC: 45,XY,-6<br>BL: 46,XY,r(6)(p25q27)[44]/45,XY,-6[6]	11857558	Ring chromosome 6 in three fetuses: case reports, literature review, and implications for prenatal diagnosis	Urban M, Bommer C, Tennstedt C, Lehmann K, Thiel G, Wegner RD, Bollmann R, Becker R, Schulzke I,	Am J Med Genet. 2002 Mar 1;108(2):97-104. doi: 10.1002/ajmg.10215.	2002
1	RC6-20 case 2	pn	M		top	BL: mos 46,XY,r(6)(p25q27)[92]/45,XY,-6[8]	11857558				
1	RC6-20 case 3	pn	M		top	AC: r(6)	11857558				
1	RC6-21	2 m	M	de novo		46,XY,r(6)(p25q27)[0.84]/r-var[0.16]	14708101	FOXC1 gene deletion is associated with eye anomalies in ring chromosome 6	Zhang HZ, Li P, Wang D, Huff S, Nimmakayalu M, Qumsiyeh M, Pober	Am J Med Genet A. 2004 Jan 30;124A(3):280-7. doi:	2004
1	RC6-22	pn	M	de novo	stillbirth	46,XY,r(6)(p25q27)[21]/45,XY,-6[47]	16053913	Prenatal diagnosis of ring chromosome 6 in a fetus with cerebellar hypoplasia and partial agenesis of corpus callosum: case report and review of the literature	Andrieux J, Devisme L, Valat AS, Robert Y, Frnka C, Savary JB.	Eur J Med Genet. 2005 Apr-Jun;48(2):199-206. doi: 10.1016/j.ejmg.2005.01.028. Epub 2005 Feb 12.	2005
1	RC6-23	15 y	F	de novo	> 24 y, birth of a healty boy	46,XX,r(6)(p25.3q27)[16]/45,XX,-6[2]/r-var[1]	18302251	Molecular characterization of a de novo ring chromosome 6 in a growth retarded but otherwise healthy woman	Höckner M, Utermann B, Erdel M, Fauth C, Utermann G, Kotzot D.	Am J Med Genet A. 2008 Apr 1;146A(7):925-9. doi: 10.1002/ajmg.a.32251.	2008
1	RC6-24	5 y	M	de novo		46,XY,r(6)[45]/46,XY[15]	18485670	An epileptic case with mosaic ring chromosome 6 and 6q terminal deletion	Kara N, Okten G, Guneş SO, Saglam Y, Tasdemir HA, Pinarli FA.	Epilepsy Res. 2008 Aug;80(2-3):219-23. doi:	2008
1	RC6-25	3 m	M	de novo		46,XY,r(6)(p25q27)	21063149	De novo ring chromosome 6 in a child with multiple congenital anomalies	Ahzad HA, Ramli SF, Loong TM, Salahshourifar I, Zilfalil BA, Yusoff NM.	Kobe J Med Sci. 2010 Sep 28;56(2):E79-84.	2010
1	RC6-26	nb	M			46,XY,r(6)(p25q27)/46,XY,dic r(6;6)	22876064	Mosaic ring chromosome 6 in an infant with significant patent ductus arteriosus and multiple congenital anomalies	Lee SJ, Han DK, Cho HJ, Cho YK, Ma JS.	J Korean Med Sci. 2012 Aug;27(8):948-52. doi: 10.3346/jkms.2012.27.8.948. Epub 2012 Jul 25.	2012
1	RC6-27	5 m	F			46,XX,r(6)(p25q27)	23398904	Array-CGH characterization and genotype-phenotype analysis in a patient with a ring chromosome 6	Ciocca L, Surace C, Digilio MC, Roberti MC, Sirleto P, Lombardo A, Russo S, Brizi V, Grotta S, Cini C, Angioni A.	BMC Med Genomics. 2013 Feb 11;6:3. doi: 10.1186/1755-8794-6-3.	2013
1	RC6-28	3 y	F			46,XX,r(6)(p25q27)[67]/45,XX,-6[25]/r-var[8]	26213576	Periventricular heterotopia and white matter abnormalities in a girl with mosaic ring	Nishigaki S, Hamazaki T, Saito M, Yamamoto T, Seto T, Shintaku H.	Mol Cytogenet. 2015 Jul 26;8:54. doi: 10.1186/s13039-015-0162-3.	2015
1	RC6-29	6 y	F	de novo		BL: 46,XX,r(6)(p25.3q27)[81]/45,XX,-6[7]<br>SF: 46,XX,r(6)(p25.3q27)[47]/45,XX,-6[3]	27103944	Molecular characterization of a novel ring 6 chromosome using next generation sequencing	Zhang R, Chen X, Li P, Lu X, Liu Y, Li Y, Zhang L, Xu M, Cram DS.	Mol Cytogenet. 2016 Apr 21;9:33. doi: 10.1186/s13039-016-0245-9. eCollection 2016.	2016
1	RC6-30	nb	M	de novo	> 49 y	46,XY,r(6)	28344652	Molecular cytogenetic characterisation of a novel de novo ring chromosome 6 involving a terminal 6p deletion and terminal 6q duplication in the different arms of the same chromosome	Pace NP, Maggouta F, Twigden M, Borg I.	Mol Cytogenet. 2017 Mar 23;10:9. doi: 10.1186/s13039-017-0311-y. eCollection 2017.	2017
1	RC6-31	2 y	M	de novo		46,XY,r(6)[0.82]/46,XY[0.06]/r-var[0.12]	29656294	Gray Matter Heterotopia, Mental Retardation, Developmental Delay, Microcephaly, and Facial Dysmorphisms in a Boy with Ring Chromosome 6: A 10-Year Follow-Up and Literature Review	Liu S, Wang Z, Wei S, Liang J, Chen N, OuYang H, Zeng W, Chen L, Xie X, Jiang J.	Cytogenet Genome Res. 2018;154(4):201-208. doi: 10.1159/000488692. Epub 2018 Apr 14.	2018
1	RC6-32	11 y	M	de novo		46,XY,r(6)(p25.3q27)[54]/45,XY,-6[13]/46,XY[6]/r-var[15]	30305128	A child with intellectual disability and dysmorphism due to complex ring chromosome 6: identification of molecular mechanism with review of literature	Sheth F, Liehr T, Shah V, Shah H, Tewari S, Solanki D, Trivedi S, Sheth J.	Ital J Pediatr. 2018 Oct 11;44(1):114. doi: 10.1186/s13052-018-0571-0.	2018
1	RC6-33	nb	M	nd		46,XY,r(6)(p24q25)	30225942	Ring chromosome 6 in a child with anterior segment dysgenesis and review of its overlap with other FOXC1 deletion phenotypes	Corona-Rivera JR, Corona-Rivera A, Zepeda-Romero LC, Rios-Flores IM, Rivera-Vargas J, Orozco-Vela M, Santana-Bejarano UF, Torres-Anguiano E, Pinto-Cardoso M, David D, Bobadilla-Morales L.	Congenit Anom (Kyoto). 2019 Sep;59(5):174-178. doi: 10.1111/cga.12309. Epub 2018 Oct 9.	2019

1	RC6-34	10 y	F	de novo		46,XX,r(6)(p25q27)	34622791	Restrictive cardiomyopathy with ring chromosome 6 anomaly in a child	Sunkak S, Kiraz A, Argun M, Erdoğan İ.	Anatol J Cardiol. 2021 Oct;25(10):745-746. doi: 10.5152/AnatolJCardiol.2021.80820 .	2021
**39**	**35**										
1	RC7-1 case 1	1 y	M			46,XY,r(7)[65]/45,XY,-7[1]/46,XY,dic r(7)[3]/46,XY[1]; 46,XY,r(7)[57]/45,XY,-7[2]/r-var[3]/46,XY[1]	4145271	Ring chromosome 7 with variable phenotypic expression	Zackai EH, Breg WR.	Cytogenet Cell Genet. 1973;12(1):40-8. doi: 10.1159/000130436.	1973
1	RC7-1 case 2	1.5 y	M			46,XY,r(7)[52]/45,XY,-7[4]/r-var[4]/46,XY[2]	4145271				
1	RC7-2	8.5 y	F	de novo	> 18 y	46,XX,r(7)[98/100]	7172483	A fourth case of ring chromosome 7	DeLozier CD, Theintz G, Sizonenko P, Engel E.	Clin Genet. 1982 Aug;22(2):90-8. doi: 10.1111/j.1399-0004.1982.tb01419.x.	1982
0	RC7-2						6705244	Cytogenetics of ring chromosome 7	DeLozier-Blanchet CD, Guenin R.	Clin Genet. 1984 Jan;25(1):84-5. doi: 10.1111/j.1399-0004.1984.tb00468.x.	1984
1	RC7-3	5 m	F	de novo		46,XX,r(7)[46/50]	3150243	Ring chromosome 7: report of a case	Kohyama J, Watanabe S, Nakajima M, Suzumura H, Ishikawa T, Ishikawa K,	Acta Paediatr Jpn. 1988 Aug;30(4):517-9. doi:	1988
1	RC7-4	3 y	M	de novo		46,XY,r(7)[0.63]	2189730	Ring chromosome 7: report of the fifth case	Caramia GM, Baroncini A, Osimani P, Forabosco A.	Eur J Pediatr. 1990 Apr;149(7):475-6. doi: 10.1007/BF01959398.	1990
1	RC7-5	nb	M	mat nl, pat nd	> 39 y	46,XY,r(7)	2395166	Ring chromosome 7 in a man with multiple congenital anomalies and mental retardation	Koiffmann CP, Diament A, de Souza DH, Wajntal A.	J Med Genet. 1990 Jul;27(7):462-4. doi: 10.1136/jmg.27.7.462.	1990
1	RC7-6	nb	M	de novo	d. 1 m resp/renal failure	46,XY,r(7)(p22q36)	1746606	Severe anomalies associated with ring chromosome 7	Biesecker LG, Cox B, Glover TW.	Am J Med Genet. 1991 Sep 15;40(4):429-31. doi: 10.1002/ajmg.1320400410.	1991
0	RC7-7						1415332	Ring chromosome 7, hyperpigmented skin lesions and malignant melanoma	DeLozier-Blanchet CD, Masouyé I, Vollenweider S.	Am J Med Genet. 1992 Aug 1;43(6):1039-40. doi: 10.1002/ajmg.1320430626.	1992
1	RC7-8	8.5 y	F	de novo		46,XX,r(7)	8428043	Melanoma associated with ring chromosome 7	Vollenweider Roten S, Delozier-Blanchet CD, Masouyé I, Saurat JH.	Dermatology. 1993;186(2):138-43. doi: 10.1159/000247325.	1993
0	RC7-9						8428052	Cutaneous findings in ring chromosome 7 syndrome	Vollenweider Roten S, Masouyé I, Delozier-Blanchet CD, Saurat JH.	Dermatology. 1993;186(2):84-7. doi: 10.1159/000247313.	1993
1	RC7-10	nb	M	de novo	d. 20 m	46,XY,r(7)(p22q36)	8362903	Case of ring chromosome 7: the first report of neuropathological findings	Tsukamoto H, Sakai N, Taniike M, Nakatsukasa M, Yoshiwara W, Sakamoto H, Fujimura H, Inui K, Okada	Am J Med Genet. 1993 Jul 1;46(6):632-5. doi: 10.1002/ajmg.1320460606.	1993
1	RC7-11	8 y	M	mat nl, pat nd	> 18 y	46,XY,r(7)(p22.3q36.3)/45,XY,-7	8922097	Boy with a ring 7 chromosome: a case report with special reference to dermatological findings	Wahlström J, Bjarnason R, Rosdahl I, Albertsson-Wikland K.	Acta Paediatr. 1996 Oct;85(10):1256-60. doi: 10.1111/j.1651-2227.1996.tb18243.x.	1996
1	RC7-12	nb	M		d. 5 m pneumonia	46,XY,r(7)(p22q36)[90]/45,XY,-7[8]/r-var[2]	8911601	Sub-band deletion of 7q36.3 in a patient with ring chromosome 7: association with holoprosencephaly	Sawyer JR, Lukacs JL, Hassed SJ, Arnold GL, Mitchell HF, Muenke M.	Am J Med Genet. 1996 Oct 16;65(2):113-6. doi: 10.1002/(SICI)1096-	1996
1	RC7-13	1.5 y	F			46,XX,UPD(7)mat[30]/47,XX,UPD7mat,+r(7) pat[15]	10227403	47,XX,UPD(7)mat,+r(7)pat/46,XX,UPD(7)mat mosaicism in a girl with Silver-Russell syndrome (SRS): possible exclusion of the putative SRS gene from a 7p13-q11 region	Miyoshi O, Kondoh T, Taneda H, Otsuka K, Matsumoto T, Niikawa N.	J Med Genet. 1999 Apr;36(4):326-9.	1999
0	RC7-14						10905891	Silver-Russell syndrome and ring chromosome 7	Wakeling EL, Hitchins M, Stanier P, Monk D, Moore GE, Preece MA.	J Med Genet. 2000 May;37(5):380. doi: 10.1136/jmg.37.5.380.	2000
1	RC7-15	nb	F	de novo		46,XX,r(7)	10982483	Ring chromosome 7 and sacral agenesis	Rodríguez L, Sanchís A, Villa A, Cánovas A, Peris S, Estívalis M, Pons S, Martínez-Frías ML.	Am J Med Genet. 2000 Sep 4;94(1):52-8. doi: 10.1002/1096-8628(20000904)94:1<52::aid-ajmg11>3.0.co;2-q.	2000
1	RC7-16	1.5 y	M			46,XY,r(7)[39]/r-var[3[/45,X-7[1]	12431259	Ring syndrome caused by ring chromosome 7 without loss of subtelomeric sequences	Vermeesch JR, Baten E, Fryns JP, Devriendt K.	Clin Genet. 2002 Nov;62(5):415-7. doi: 10.1034/j.1399-0004.2002.620511.x.	2002
1	RC7-17	nb	M			46,XY,r(7)(p22q36)	15523614	Somatic mosaicism of chromosome 7 in a highly proliferating melanocytic congenital naevus in a ring chromosome 7 patient	Mehraein Y, Ehlhardt S, Wagner A, Göttert E, Tilgen W, Zang KD, Dill-Müller D.	Am J Med Genet A. 2004 Dec 1;131(2):179-85. doi: 10.1002/ajmg.a.30370.	2004
1	RC7-18	?	F			46,XX,r(7)/46,XX	18300171	Ring chromosome 7 in an Indian woman	Kaur A, Dhillon S, Garg PD, Singh JR.	J Intellect Dev Disabil. 2008	2008
1	RC7-19	10 m	F			46,XX,r(7)(p22q36)[75]/45,XX,-7[12]/r-var[11]	21303521	Molecular cytogenetic analysis and clinical manifestations of a case with de novo mosaic ring chromosome 7	Tsai LP, Lee KF, Fang JS, Liu IY.	Mol Cytogenet. 2011 Feb 8;4(1):5. doi: 10.1186/1755-8166-4-5.	2011
1	RC7-20	pn	F	de novo	top	46,XX,r(7)(p22q36)[2]	23509645	First trimester diagnosis of holoprosencephaly secondary to a ring chromosome 7	Henderson LB, Corson VL, Saul DO, Anderson C, Millard S, Batista DA,	Case Rep Genet. 2013;2013:578202. doi:	2013

1	RC7-21	1.5 y	M	de novo		PL: 46,XY,r(7)(p22q36.3), i/d-SF: 45,XY,-7/46,XY,r(7)	24677512	Cytogenomic and phenotypic analysis in low-level monosomy 7 mosaicism with non-supernumerary ring chromosome 7	Salas-Labadía C, Cervantes-Barragán DE, Cruz-Alcívar R, Daber RD, Conlin LK, Leonard LD, Spinner NB, Durán-McKinster C, Dávila-Ortíz de	Am J Med Genet A. 2014 Jul;164A(7):1765-9. doi: 10.1002/ajmg.a.36503. Epub 2014 Mar 26.	2014
1	RC7-22	13 y	F			46,XX,r(7)(p22q36)[2]/85-91,idemx2[cp10]/46,XX[9]	33678971	Ring chromosome 7 in a child with T-cell acute lymphoblastic leukemia with myeloid markers	Tirado CA, Reyes A, Yeh W	Proc (Bayl Univ Med Cent). 2021 Jan 19;34(2):302-304	2021
1	RC7-23	22 y	M		> 22 y	46,XY,r(7)	33522711	Multiple desmoplastic Spitz nevi with BRAF fusions in a patient with ring chromosome 7	Roy SF, Bastian BC, Maguiness S, Giubellino A, Vemula SS, McCalmont	Pigment Cell Melanoma Res. 2021 Sep;34(5):987-993	2021
**21**	**24**										
1	RC8-1		M				4795572	[Ring chromosome 8 (46,XY, 8 r) in a boy with debility (author's transl)]	Pfeiffer RA, Lenard HG.	Klin Padiatr. 1973 May;185(3):187-91.	1973
1	RC8-2	5 y	M	de novo		SF: 46,XY,r(8)	604497	Ring chromosome 8 in a boy with multiple congenital abnormalities and mental retardation	Hamers AJ, van Kempen C.	J Med Genet. 1977 Dec;14(6):451-5. doi: 10.1136/jmg.14.6.451.	1977
1	RC8-3	2 y	F			46,XX,r(8)	1746890	Ring chromosome 8 associated with microcephaly	Mingarelli R, Valorani G, Zelante L, Dallapiccola B.	Ann Genet. 1991;34(2):90-2.	1991
1	RC8-4	13 y	F			46,XX,inv(7)(p22q11.23),r(8)(p23q24.3)	1552550	Pericentric inversion of chromosome 7 (inv(7)(p22q11.2)) and ring chromosome 8 (r(8)(p23q24.3)) in a girl with minor anomalies	Verma RS, Conte RA, Pitter JH, Luke S.	J Med Genet. 1992 Jan;29(1):66-7. doi: 10.1136/jmg.29.1.66.	1992
1	RC8-5	12 y	M	de novo		46,XY,r(8)	11391511	[Ring chromosome 8: microcephaly, mental retardation and minor facial anomalies with adhesive behavioral phenotype]	Bibas Bonet H, Fontenla M, Fauze R, G de Pinat I.	Rev Neurol. 2001 Apr 16-30;32(8):746-50.	2001
1	RC8-6	6.5 y	M	mat	>~18 y mat	46,XY,r(8)(p23q24.3)[24]/45,XY,-8[2]	15337475	Inherited ring chromosome 8 without loss of subtelomeric sequences	Le Caignec C, Boceno M, Jacquemont S, Nguyen The Tich S, Rival JM, David A.	Ann Genet. 2004 Jul-Sep;47(3):289-96. doi: 10.1016/j.anngen.2003.10.005.	2004
1	RC8-7	nb	M	de novo	> ~ 20 y	46,XY,r(8)/46,XY	16829350	Monosomy 8 rescue gave cells with a normal karyotype in a mildly affected man with 46,XY,r(8) mosaicism	Gradek GA, Kvistad PH, Houge G.	Eur J Med Genet. 2006 Jul-Aug;49(4):292-7. doi: 10.1016/j.ejmg.2005.08.004. Epub 2005 Oct 6.	2006
1	RC8-8						24551990	Ring chromosome 8 with mosaic trisomy 8 syndrome in an infant	Imataka G, Ishii J, Tsukada K, Suzumura H, Arisaka O.	Genet Couns. 2013;24(4):441-4.	2013
1	RC8-9	1 y	M			46,XY,r(8)(p23q24.3)[27]/45,XY,-8[3]	33316910	46,XY,r(8)/45,XY,-8 Mosaicism as a Possible Mechanism of the Imprinted Birk-Barel Syndrome: A Case Study	Kashevarova AA, Nikitina TV, Mikhailik LI, Belyaeva EO, Vasilyev SA, Lopatkina ME, Fedotov DA, Fonova EA, Zarubin AA, Sivtsev AA, Skryabin NA,	Genes (Basel). 2020 Dec 9;11(12):1473. doi: 10.3390/genes11121473.	2020
**9**	**9**										
1	RC9-1	2 m	M			46,XX,r(9)	5445002	Comparative behavior of ring chromosomes	Kistenmacher ML, Punnett HH	Am J Hum Genet 22(3):304-18, 1970	1970
1	RC9-2	22 y	F	de novo	> 22 y	46,XX,r(9)	4127395	A ring chromosome, diagnosed by quinacrine fluorescence as No. 9, in a mentally retarded girl	P Jacobsen, M Mikkelsen, F Rosleff	Clin Genet	1973
1	RC9-3						4548818	A case of annular chromosome 9. identification by controlled denaturation	J Fraisse, B Lauras, M J Ooghe, F Freycon, M O Rethoré	Ann Genet	1974
1	RC9-4	3 y	M (AG)			46,XY,r(9)(p24q34)	939548	Mental retardation and congenital malformations associated with a ring chromosome 9	Nakajima S, Yanagisawa M, Kamoshita S, Nakagome Y.	Hum Genet. 1976 Jun 29;32(3):289-93. doi: 10.1007/BF00295818.	1976
1	RC9-5	4 y	M (AG)			46,XY,r(9)	885556	Ring chromosome 9. 46,XY,r(9) in a male with ambiguous external genitalia	Metaxotou C, Kalpini-Mavrou A.	Hum Genet. 1977 Jul 26;37(3):351-4. doi: 10.1007/BF00393619.	1977
1	RC9-6	nb	M			46,XY,r(9)	468257	Moderate mental retardation and nonspecific dysmorphic syndrome associated with ring chromosome 9	Fryns JP, Lambrechts A, Jansseune H, Van den Berghe H.	Hum Genet. 1979;50(1):29-32. doi: 10.1007/BF00295585.	1979
1	RC9-7	nb	M		d. 9 d cardiac arrest	46,XY,r(9)(p24w34)	489005	A ring chromosome 9 in an infant with malformations	Inouye T, Matsuda H, Shimura K, Hamazaki M, Kikuta I, Iinuma K, Nakagome Y.	Hum Genet. 1979 Sep;50(3):231-5. doi: 10.1007/BF00399386.	1979
1	RC9-8						6982668	[A new case of ring chromosome 9]	Portnoi MF, van den Akker J, Le Porrier N, Joye N, Youssef S, Taillemite JL.	Ann Genet. 1982;25(3):164-7.	1982
1	RC9-9						6985015	Ring chromosome 9: identification of a new case by G- and C-banding	Dipierri JE, Matayoshi T.	Ann Genet. 1982;25(4):243-5.	1982
1	RC9-10						6301043	[A new case of ring chromosome 9]	Portnoi MF, van den Akker J, Le Porrier N, Joye N, Youssef S, Taillemite JL.	Sem Hop. 1983 Jan 20;59(3):185-8.	1983
1	RC9-11	25 y	F		> 25 y	46,XX,r(9)(p24q34)	3130846	Apparent Prader-Willi phenotype in a woman with ring chromosome 9	Hess RO, Meisner LF.	Am J Med Genet Suppl. 1987;3:133-8. doi: 10.1002/ajmg.1320280515.	1987

1	RC9-12	nb	M	de novo		46,XY,r(9)(p24.1q34.3)	3149132	A patient with ring 9 chromosome 46,XY,r(9)(p24.1q34.3)	Kasa N, Kasai R.	Acta Paediatr Jpn. 1988 Dec;30(6):710-3. doi: 10.1111/j.1442-200x.1988.tb02558.x.	1988
1	RC9-13		F				3265311	[Ring chromosome 9. Case report and review of the literature]	Manouvrier-Hanu S, Turck D, Gottrand F, Savary JB, Loeuille GA, Deminatti MM, Farriaux JP.	Ann Genet. 1988;31(4):250-3.	1988
1	RC9-14	3 m	F		d.2 y pneumonia	46,XX,r(9)	2774007	Another case of ring chromosome 9 associated with gastroesophageal reflux	Manouvrier-Hanu S, Turck D, Farriaux JP.	Am J Med Genet. 1989 Apr;32(4):558. doi: 10.1002/ajmg.1320320432.	1989
1	RC9-15	nb	M				1781957	Ring chromosome 9 in a newborn male presenting with facial dysmorphia, hypospadias and skeletal abnormalities	Van Maldergem L, Avni F, Mossay B, Herens C, Verloes A, Gillerot Y.	Genet Couns. 1991;2(2):123-7.	1991
1	RC9-16						8674446	[Ring chromosome 9]	Kontiokari T, Borgström GH, Ritanen-	Duodecim. 1995;111(5):439-41.	1995
1	RC9-17	6 m	M	de novo		46,XY,r(9)(p24q34)	8836504	Ring chromosome 9: an atypical case	Lanzi G, Fazzi E, Veggiotti P, Pagliano E, Gariglio M, Bonaglia C, Landolfo S.	Brain Dev. 1996 May-Jun;18(3):216-9. doi: 10.1016/0387-7604(95)00144-1.	1996
0	RC9-18						10021722	Phenotypic variability in the chromosome 9 ring	Cavaliere ML, Rinaldi MM, Castelluccio P, Cioffi C, Vendemmia M, Vendemmia S.	Acta Biomed Ateneo Parmense. 1997;68 Suppl 1:85-9.	1997
1	RC9-19	6 m	F	de novo		46,XX,r(9)[45/50]	10486077	Ring chromosome 9 with a 9p22.3-p24.3 duplication	Seghezzi L, Maraschio P, Bozzola M, Maserati E, Tupler R, Marchi A, Tiepolo L.	Eur J Pediatr. 1999 Oct;158(10):791-3. doi: 10.1007/s004310051206.	1999
1	RC9-20 case 1	16 y	F			46,XX,r(9)(p24q34)	16158426	Ring chromosome 9 [r(9)(p24q34)]: a report of two cases	Purandare SM, Lee J, Hassed S, Steele MI, Blackett PR, Mulvihill JJ, Li S.	Am J Med Genet A. 2005 Oct 15;138A(3):229-35. doi: 10.1002/ajmg.a.30382.	2005
1	RC9-20 case 2	8 m	M	de novo		46,XY,r(9)(p24q34)	16158426				
1	RC9-21	pn	M	de novo	top	45,XY,-9[9]/46,XY,r(9)([24q34.3)[34]/r-var[3]	16941502	Prenatal diagnosis of mosaic ring chromosome 9	Chen CP, Lin CL, Chen LL, Lee CC, Wang W.	Prenat Diagn. 2006 Sep;26(9):870-1. doi: 10.1002/pd.1515.	2006
1	RC9-22	8 m	M			46,X,inv(Y),r(9)(p24q34)	17526969	Ring chromosome 9 in a dysmorphic child	Sheth J, Joshi R, Sheth F.	Indian J Pediatr. 2007 May;74(5):507-8. doi: 10.1007/s12098-007-0090-2.	2007
1	RC9-23	8 m	F			45,XX,-9[4]/46,XX,r(9)(p24q34)[92]/r-var[4]	20931537	[Analysis of ring chromosome 9 syndrome with fluorescence in situ hybridization]	Ye ZC, Zhu XY, Zhao R, He XY, Zhang HJ, Li LP, Zhu YM.	Zhonghua Yi Xue Yi Chuan Xue Za Zhi. 2010 Oct;27(5):559-62. doi: 10.3760/cma.j.issn.1003-9406.2010.05.018.	2010
1	RC9-24	nb					24551976	Ring chromosome 9 in a newborn	Aldemir O, Celik IH, Karaer K, Ceylaner G.	Genet Couns. 2013;24(4):357-60.	2013
1	RC9-25	7 m	F			46,XX,r(9)(p23q34.3)[96]/45,XX,-9[4]	23633410	Ring chromosome 9 in a girl with developmental delay and dysmorphic features: case report and review of the literature	la Cour Sibbesen E, Jespersgaard C, Alosi D, Bisgaard AM, Tümer Z.	Am J Med Genet A. 2013 Jun;161A(6):1447-52. doi: 10.1002/ajmg.a.35901. Epub 2013 Apr 30.	2013
1	RC9-26	pn	F	de novo	top	46,XX,r(9)(p24q34)/45,XX,-9[0.16]	25722017	Prenatal diagnosis of a female fetus with ring chromosome 9, 46,XX,r(9)(p24q34), and a de novo interstitial 9p deletion	Penacho V, Galán F, Martín-Bayón TA, Mayo S, Manchón I, Carrasco A, Martínez-Castellano F, Alcaraz LA.	Cytogenet Genome Res. 2014;144(4):275-9. doi: 10.1159/000370256. Epub 2015 Feb 20.	2014
1	RC9-27	30 y	M		> 30 y	46,XY,r(9)(p24.3q34.3)[42]/45,XY,-9[5]/46,XY[3]	25449292	Azoospermia and ring chromosome 9--a case report	Laursen RJ, Tüttelmann F, Humaidan P, Elbæk HO, Alsbjerg B, Röpke A.	J Assist Reprod Genet. 2015 Feb;32(2):293-6. doi: 10.1007/s10815-014-0388-8. Epub 2014 Dec 2.	2015
1	RC9-28	2 y	M	de novo	d. 7 y	46,XY,r(9)(p22q34)[89]/45,XY,-9[4]/r-var[7]	27222354	Ring Chromosome 9 and Chromosome 9p Deletion Syndrome in a Patient Associated with Developmental Delay: A Case Report and Review of the Literature	Sivasankaran A, Kanakavalli MK, Anuradha D, Samuel CR, Kandukuri LR.	Cytogenet Genome Res. 2016;148(2-3):165-73. doi: 10.1159/000445862. Epub 2016 May 26.	2016
1	RC9-29	6 m	F (XY)	de novo		45,XY,-9[3]/46,XY,r(9)[96]/r-var[6]	26829739	[Analysis of genetics mechanism for the phenotypic diversity in a patient carrying a rare ring chromosome 9]	Qin S, Wang X, Li Y, Wei P, Chen C, Zeng L.	Zhonghua Yi Xue Yi Chuan Xue Za Zhi. 2016 Feb;33(1):71-5. doi: 10.3760/cma.j.issn.1003-9406.2016.01.018.	2016
1	RC9-30	6 m	F (XY)			46,XY,r(9)[29].45,XY,-9[8]/r-var[2]/46,XY[1]	29760778	Loss of DMRT1 gene in a Mos 45,XY,-9[8]/46,XY,r(9)[29]/47,XY,+idic r(9)× 2[1]/46,XY,idic r(9)[1]/46,XY[1] female presenting with short stature	Marsudi BA, Kartapradja H, Paramayuda C, Batubara JRL, Harahap AR, Marzuki NS.	Mol Cytogenet. 2018 May 8;11:28. doi: 10.1186/s13039-018-0379-z. eCollection 2018.	2018
1	RC9-31	2 m	F	de novo		45,XX,-9[4]/46,XX,r(9)(p24q34)[56]	31400141	[Kleefstra syndrome 1 and ring chromosome 9 in a case]	Lyu N, Li D, Li J, Shang Q, Ma C.	Zhonghua Yi Xue Yi Chuan Xue Za Zhi. 2019 Aug 10;36(8):837-840. doi: 10.3760/cma.j.issn.1003-9406.2019.08.021.	2019
1	RC9-32	21 y	M	adopted	> 21 y	46,XY,r(9)[100]/45,XY,-9[20]	32978894	Ring chromosome formation by intra-strand repairing of subtelomeric double stand breaks and clinico-cytogenomic correlations for ring	Chai H, Ji W, Wen J, DiAdamo A, Grommisch B, Hu Q, Szekely AM, Li P.	Am J Med Genet A. 2020 Dec;182(12):3023-3028. doi: 10.1002/ajmg.a.61890. Epub 2020	2020

**32**	**32**										
1	RC10-1	9 y	F	de novo		46,XX,r(10)	839503	Physical retardation is associated with ring chromosome mosaicism: 46, XX,r(10)/45, XX,10	Lansky S, Daniel W, Fleiszar K.	J Med Genet. 1977 Feb;14(1):61-3. doi: 10.1136/jmg.14.1.61.	1977
1	RC10-2	15 m	F	de novo		46,XX,r(10)(p15q26)	700708	Ring 10 chromosome: 46,XX,r10(p15q26)	Sparkes RS, Ling SM, Muller H.	Hum Genet. 1978 Sep 19;43(3):341-5. doi: 10.1007/BF00278844.	1978
1	RC10-3	nb	M	de novo		46,XY,r(10),t(10;19)(q25;p13)	567620	Malformative syndrome associated with a ring 10 chromosome and a translocated 10q/19 chromosome	Fryns JP, De Boeck K, Jaeken J, van den Berghe H.	Hum Genet. 1978 Aug 31;43(2):239-44. doi: 10.1007/BF00293602.	1978
1	RC10-4	8 m	M	de novo		PB: 46,XY,r(10)[192/200], SF: 46,XY,r(10)[182/200]	511137	Ring chromosome 10 associated with multiple congenital malformations	Simoni G, Rossella F, Dalprà L, Visconti G, Piria-Schwarz C.	Hum Genet. 1979 Oct 1;51(2):117-21. doi: 10.1007/BF00287164.	1979
1	RC10-5	8 m	F	de novo		46,XX,r(10)(p15q26)	7381872	Ring chromosome 10:46,XX,r(10)(p15 leads to q26)	Tsukino R, Tsuda N, Dezawa T, Ishii T, Koike M.	J Med Genet. 1980 Apr;17(2):148-50. doi: 10.1136/jmg.17.2.148.	1980
1	RC10-6	nb	M	de novo		46,XY,r(10)[84]/45,XY,-10[13]/r-var[1]	7025632	Phenotype associated with ring 10 chromosome: report of patient and review of literature	Michels VV, Driscoll DJ, Ledbetter DH, Riccardi VM.	Am J Med Genet. 1981;9(3):231-7. doi: 10.1002/ajmg.1320090309.	1981
1	RC10-7		F				6982669	[Ring chromosome 10: 46,XX,r(10)(p15q26)]	Serville F, Briault R, Taillemite JL, Despoisse S, Cotoni P, Broustet A.	Ann Genet. 1982;25(3):168-71.	1982
1	RC10-8	2 m	M	de novo		46,XY,r(10)[91]/45,XY,-10[5]/r-var[4]	6842550	Ring chromosome 10 and its clinical features	Nakai H, Adachi M, Katsushima N, Yamazaki N, Sakamoto M, Tada K.	J Med Genet. 1983 Apr;20(2):142-4. doi: 10.1136/jmg.20.2.142.	1983
1	RC10-9	2 y	M	mat nl,		46,XY,r(10)(p15.3q26.3)	6705254	Ring chromosome 10 syndrome: case report and the possibility of clinical diagnosis	Kondo I, Shimakura Y, Hirano T, Kaneko M, Yabuta K.	Clin Genet. 1984 Feb;25(2):196-200. doi: 10.1111/j.1399-0004.1984.tb00485.x.	1984
1	RC10-10	2.5 y	M			mos 45,XY,-10/46,XY/46,XY,r(10)(p15.3q26.3)	3834204	Report of a patient with a ring chromosome 10: mos45,XY,-10/46,XY/46,XY,r(10)(p15.3q26.3)	Kishi K, Ikeuchi T, Yamamoto K, Tonomura A, Sakurada N, Satoh Y.	Jinrui Idengaku Zasshi. 1985 Sep;30(3):233-8. doi: 10.1007/BF01876474.	1985
1	RC10-11	nb	F		d. 3m, renal failure	PB/SF: 46,XX,r(10)	7837258	A newborn with ring chromosome 10, aganglionic megacolon, and renal hypoplasia	Calabrese G, Franchi PG, Stuppia L, Mingarelli R, Rossi C, Ramenghi L, Marino M, Morizio E, Peila R,	J Med Genet. 1994 Oct;31(10):804-6. doi: 10.1136/jmg.31.10.804.	1994
1	RC10-12	32 y	M		> 32 y	46,XY,r(10)(p15q26)	14598349	Ring chromosome 10 (p15q26) in a patient with unipolar affective disorder, multiple minor anomalies, and mental retardation	Concolino D, Iembo MA, Moricca MT, Strisciuglio P, Marotta R, Rossi E, Giglio S.	Am J Med Genet A. 2003 Dec 1;123A(2):201-3. doi: 10.1002/ajmg.a.20299.	2003
1	RC10-13	nb	F	de novo		46,XX,r(10)	19968867	Chromosome r(10)(p15.3q26.12) in a newborn child: case report	Gunnarsson C, Graffmann B, Jonasson J.	Mol Cytogenet. 2009 Dec 7;2:25. doi: 10.1186/1755-8166-2-25.	2009
1	RC10-14	pn	M	de novo	TOP, 27 gwks	46,XY,r(10)(p15.3q26.3)	21914491	Clinical and molecular description of a fetus in prenatal diagnosis with a rare de novo ring 10 and deletions of 12.59 Mb in 10p15.3-p14 and 4.22 Mb in 10q26.3	Christopoulou G, Tzetis M, Konstantinidou AE, Tsezou A, Kanavakis E, Kitsiou-Tzeli S, Velissariou V.	Eur J Med Genet. 2012 Jan;55(1):75-9. doi: 10.1016/j.ejmg.2011.08.002. Epub 2011 Sep 9.	2012
1	RC10-15 case 1	2.5 y	M	de novo	d. 15 y	46,XY,r(10)(p15.3q26.2)	23247912	Ring chromosome 10: report on two patients and review of the literature	Guilherme RS, Kim CA, Alonso LG, Honjo RS, Meloni VA, Christofolini DM, Kulikowski LD, Melaragno MI.	J Appl Genet. 2013 Feb;54(1):35-41. doi: 10.1007/s13353-012-0128-7. Epub 2012 Dec 18.	2013
1	RC10-15 case 2	nb	F	de novo		46,XX,r(10)(p15.3q26.3)	23247912				
1	RC10-16	7 m	F	nd		46,XX,r(10)(p15.1q26.1)	25922618	Clinical, cytogenetic and molecular study of a case of ring chromosome 10	Čiuladaitė Ž, Burnytė B, Vansevičiūtė D, Dagytė E, Kučinskas V, Utkus A.	Mol Cytogenet. 2015 Apr 21;8:29. doi: 10.1186/s13039-015-0124-9. eCollection 2015.	2015
1	RC10-17	nb	F			46,XX,r(10)(p15q26.1)	34680908	Expanding the Neurological Phenotype of Ring Chromosome 10 Syndrome: A Case Report and Review of the Literature	Pruccoli J, Graziano C, Locatelli C	Genes (Basel). 2021 Sep 26;12(10):1513	2021
**18**	**17**										
1	RC11-1	3 m	F	de novo		46,XX,(11)	856722	Ring 11 chromosome (46,xx,r11(p15q25))	Valente M, Muller H, Sparkes RS.	Hum Genet. 1977 May 10;36(3):345-50. doi: 10.1007/BF00446287.	1977
1	RC11-2	2 y	F			46,XX,r(11)(p15q25)	6974530	Ring chromosome 11 [46,XX,r(11) (p15q25)] associated with clinical features of the 11q-	Niikawa N, Jinno Y, Tomiyasu T, Fukushima Y, Kudo K.	Ann Genet. 1981;24(3):172-5.	1981
1	RC11-3	6 m	F		d. 1.5 y cardiac failure	46,XX,r(11)(p15q24)	6829609	Brief clinical report: ring-11 chromosome: phenotype-karyotype correlation with deletions of 11q	Cousineau AJ, Higgins JV, Scott-Emuakpor AB, Mody G.	Am J Med Genet. 1983 Jan;14(1):29-35. doi: 10.1002/ajmg.1320140106.	1983
1	RC11-4 case 1	2.5 y	F	de novo		46,XX,r(11)(p15.5q25)[0.86]	6315941	Two cases of ring chromosome 11	Romain DR, Gebbie OB, Parfitt RG, Columbano-Green LM, Smythe RH,	J Med Genet. 1983 Oct;20(5):380-2. doi: 10.1136/jmg.20.5.380.	1983
1	RC11-4 case 2	8 m	M	mat nl	d. 1.5 y	46,XY,r(11)(p15q25)					
1	RC11-5	11 y	M	de novo		46,XY,r(11)(p15.5q25)[72]/46,XY[30]/45,XY,-11[3]	3086186	Congenital ocular and other systemic abnormalities associated with ring-11	Daniele S, Pecorelli F, Tiepolo L, Armellini R, Liotti FS.	Graefes Arch Clin Exp Ophthalmol. 1986;224(3):317-20. doi:	1986
1	RC11-6	nb	F				3487279	Ring chromosome 11. A case report and review of the literature	Palka G, Verrotti A, Peca S, Mosca L, Lombardo G, Verrotti M, Morgese G.	Ann Genet. 1986;29(1):55-8.	1986

1	RC11-7	4.5 y	F	mat	> 29 y	46,XX,r(11)(p15q25)	3189413	Ring chromosome 11 and café-au-lait spots	Fagan K, Suthers GK, Hardacre G.	Am J Med Genet. 1988 Aug;30(4):911-6. doi: 10.1002/ajmg.1320300406.	1988
1	RC11-8	pn	F	de novo	top	AF: 46,XX,r(11)(p15q25)[14]/45,XX,-11[7]	11503165	Prenatal diagnosis of a de novo ring chromosome 11	Mohamed AN, Ebrahim SA, Aatre R, Qureshi F, Jacques SM, Evans MI.	Am J Med Genet. 2001 Sep 1;102(4):368-71. doi: 10.1002/ajmg.1492. PMID: 11503165.	2001
1	RC11-9	2.5 y	M	de novo		45,XY,r911)(p15.5q25)[90]/45,XY,-11[8]/r-var[2]	20583153	Constitutional ring chromosome 11 mosaicism in a Wilms tumor patient: Cytogenetic, molecular and clinico-pathological studies	Affiliations	Am J Med Genet A. 2010 Jul;152A(7):1756-63. doi: 10.1002/ajmg.a.33420.	2010
1	RC11-10 case 1	nb	M	de novo		46,XY,r(11)(p15.5q25)	22975011	Molecular and clinical characterization of patients with a ring chromosome 11	Hansson KB, Gijsbers AC, Oostdijk W, Rehbock JJ, de Snoo F, Ruivenkamp CA,	Eur J Med Genet. 2012 Dec;55(12):708-14. doi:	2012
1	RC11-10 case 2	3 y	F	de novo		46,XX,r(11)(p15.5q23.3)	22975011				
1	RC11-10 case 3	6 y	F	mat	> 37 y	46,XX,r(11)(p15.5q25)	22975011				
1	RC11-11	5 y	F			46,XX,r(11)(p15.5q25)[27]/45,XX,-11[3]	23234794	[Wilms' tumor and ring chromosome 11 in a child]	Zhang H, Feng C, Tang SQ.	Zhongguo Dang Dai Er Ke Za Zhi.	2012
1	RC11-12	5 y	F		> 20 y	46,XX,r(11)(p15q25)	26576288	Endocrine abnormalities in ring chromosome 11: a case report and review of the literature	Lange R, Von Linsingen C, Mata F, Moraes AB, Arruda M, Vieira Neto L.	Endocrinol Diabetes Metab Case Rep. 2015;2015:150085. doi: 10.1530/EDM-15-0085. Epub 2015 Oct 15.	2015
1	RC11-13	12 y	F	de novo		46,XX,r(11)(p15.3q24.1),der(21)t(11;21)(p15.3;qter)[158]/45,XX,-11[16]/r-var[5]	26557157	De Novo ring chromosome 11 and non-reciprocal translocation of 11p15.3-pter to 21qter in a patient with congenital heart disease	Peng Y, Ma R, Zhou Y, Xia Y, Wen J, Zhang Y, Guo R, Li H, Pan Q, Zhang R, Tang C, Liang D, Wu L.	Mol Cytogenet. 2015 Nov 9;8:88. doi: 10.1186/s13039-015-0191-y. eCollection 2015.	2015
1	RC11-14	13 y	M			45,XY,-11[18]/46,XY,r(11)[78]/46,XY,dic r(11)[4]	28232783	Complex Mosaic Ring Chromosome 11 Associated with Hemizygous Loss of 8.6 Mb of 11q24.2qter in Atypical Jacobsen Syndrome	Galvão Gomes A, Paiva Grangeiro CH, Silva LR, Oliveira-Gennaro FG, Pereira CS, Joaquim TM, Panepucci RA, Squire [illegible]	Mol Syndromol. 2017 Jan;8(1):45-49. doi: 10.1159/000452681. Epub 2016 Nov 17.	2017
**17**	**14**										
1	RC12-1	1 y	F	nd		46,XX,r(12)(p13q24)[0.80]/45,XX,-12[0.8]/r-var[0.12]	7395909	The syndrome of ring chromosome 12	Scribanu N, McCullars EB, Baumiller RC, Colon AR.	Am J Med Genet. 1980;5(2):165-70. doi: 10.1002/ajmg.1320050210.	1980
1	RC12-2	23 y	M	nd	> 23y	BL/SF: 46,XY,r(12)(p13.3q24.3)	3354616	Ring chromosome 12	Park JP, Graham JM Jr, Andrews PA, Wurster-Hill DH.	Am J Med Genet. 1988 Feb;29(2):437-40. doi: 10.1002/ajmg.1320290228.	1988
1	RC12-3	30 y	F		> 30 y	46,XX,r(12)(p13.3q24.3)[26]/46,XX[12]	8725781	Leiomyoma of uterus in a patient with ring chromosome 12: case presentation and literature review	Hajianpour MJ, Hajianpour AK, Habibian R, Wohlmuth C.	Am J Med Genet. 1996 May 17;63(2):335-9. doi: 10.1002/(SICI)1096-	1996
1	RC12-4	15 y	F	nd		46,XX,r(12)(p13q24.33)[0.98]/45,XX,-12[0.02]	12599192	Ring chromosome 12 with variable phenotypic features: clinical report and review of the	Parmar RC, Muranjan MN, Kotvaliwale S, Sharma S, Bharucha BA.	Am J Med Genet A. 2003 Mar 15;117A(3):275-7. doi:	2003
1	RC12-5	7 y	M	de novo		46,XY,r(12)(p13.3q24.33)[73]/46,XY,-12[8]/r-var[2]	15930904	Association of microcephaly and cafe-au-lait spots in a patient with ring chromosome 12 syndrome	Zen PR, Pinto LL, Graziadio C, Pereira VB, Paskulin GA.	Clin Dysmorphol. 2005 Jul;14(3):141-3. doi: 10.1097/00019605-200507000-00007.	2005
1	RC12-6	27 y	M		> 27 y	46,XY,r(12)(p13q24.3)[0.85]/46,XY	17880954	Ring chromosome 12 and severe oligospermia: a case report	Martin JR, Wold A, Taylor HS.	Fertil Steril. 2008 Aug;90(2):443.e13-5. doi:	2008
1	RC12-7	20 y	M	de novo	> 20 y	46,XY,r912)	20933620	Ring chromosome 12 with inverted microduplication of 12p13.3 involving the Von Willebrand Factor gene associated with cryptogenic stroke in a young adult male	Nik-Zainal S, Cotter PE, Willatt LR, Abbott K, O'Brien EW.	Eur J Med Genet. 2011 Jan-Feb;54(1):97-101. doi: 10.1016/j.ejmg.2010.09.014. Epub 2010 Oct 8.	2011
**7**	**7**										
1	RC13-1	3 y	F	mat nl, pat nd		46,XX,r(13)	5096545	Ring 13 chromosome with normal haptoglobin inheritance	Hollowell JG, Littlefield LG, Dharmkrong-AT A, Folger GM, Heath	J Med Genet. 1971 Jun;8(2):222-6. doi: 10.1136/jmg.8.2.222.	1971
1	RC13-2 case 1	nb	M			46,XY,r(13)	4691554	Ring chromosome 13 and haptoglobin heterozygosity	Fitzgerald PH.	Clin Genet. 1973;4(1):25-7. doi: 10.1111/j.1399-0004.1973.tb01117.x.	1973
1	RC13-2 case 2	1 y	F			46,XX,r(13)	4691554				
1	RC13-3						4476859	Ring 13 chromosome in an infant with congenital malformations (author's transl)	Kuroki Y, Toyota J.	Jinrui Idengaku Zasshi. 1974 Jun;19(1):77-8.	1974
1	RC13-4	1.2 y	M			PB/SF: 46,XY,r(13)[0.85/0.82]	4140830	The behavior of ring chromosome 13	Hoo JJ, Obermann U, Cramer H.	Humangenetik. 1974;24(3):161-71. doi: 10.1007/BF00283581.	1974
1	RC13-5	1 y	M	de novo		PL/SF: 46,XY,r(13)	4140834	Malformative syndrome with ring chromosome 13	Fryns JP, Deoover J, Van den Berghe H.	Humangenetik. 1974;24(3):235-40. doi: 10.1007/BF00283592.	1974
1	RC13-6						1140815	Ring chromosome 13 in a polymalformed anencephalic	Schmid W, Mühlethaler JP, Briner J, Knechtli H.	Humangenetik. 1975;27(1):63-6. doi: 10.1007/BF00283507.	1975
1	RC13-7	1.5 y	F			46,XX,r(13)[0.92]	1139790	Ring chromosome 13 syndrome	Fried K, Rosenblatt M, Mundel G, Krikler R.	Clin Genet. 1975 Mar;7(3):203-8. doi: 10.1111/j.1399-0004.1975.tb00320.x.	1975

1	RC13-8	nb	F	de novo	> 21 y	46,XX,r(13)	939570	Parental origin of a ring 13 chromosome in a female with multiple anomalies	Magenis RE, Wyandt HE, Overton KM, Macfarlane J.	Hum Genet. 1976 Jul 27;33(2):181-6. doi: 10.1007/BF00281894.	1976
1	RC13-9						890095	Ring 13 chromosome associated with microcephaly, congenital heart defect, intrauterine growth retardation, and abnormal	Lowry RB, Dill FJ.	Birth Defects Orig Artic Ser. 1977;13(3B):216-22.	1977
1	RC13-10						666131	[Ring chromosome 13 with. 13p. preserved. A case report and review of literature (author's transl]	Benítez J, Ramos MC, Escorihuela R, Hernández Merino H.	An Esp Pediatr. 1978 Mar;11(3):253-62.	1978
1	RC13-11	2.5 y	F	de novo		46,XX,r(13)(p11q34)	717293	Ring chromosome 13 in a child with minor dysmorphic features. Irregular phenotypic expression of ring 13 syndrome	Verma RS, Dosik H, Chowdhry IH, Jhaveri RC.	Am J Dis Child. 1978 Oct;132(10):1018-21. doi: 10.1001/archpedi.1978.02120350082018.	1978
1	RC13-12	10 y	F			46,XX,r(13)(p11q34)	317785	Some clinical and cytogenetic observations on a ring chromosome 13 (p11 q34)	Hernandez A, Garcia-Cruz D, Plascencia L, Nazara Z, Rivera H, Sanchez-Corona	Ann Genet. 1979;22(4):221-4.	1979
1	RC13-13						317788	[Ring chromosome 13 (author's transl)]	Hevia A, Bullon M, Novales A, Fernandez-Novoa C, San Martin V,	Ann Genet. 1979;22(4):232-3.	1979
1	RC13-14	20 y	M	mat nl, pat nd		46,XY,r(13)	7440241	Prophase analysis of ring chromosome 13--an attempt at phenotype-karyotype correlation	Lagergren M, Börjeson M, Mitelman F.	Hereditas. 1980;93(2):231-3. doi: 10.1111/j.1601-5223.1980.tb01363.x.	1980
1	RC13-15 case 1	5 y	F		> 32 y	46,XX,r(13)	7211840	Study of two cases of ring 13 chromosome using high-resolution banding	Jones IM, Palmer CG, Weaver DD, Hodes ME.	Am J Hum Genet. 1981 Mar;33(2):252-61.	1981
1	RC13-15 case 2	5 y	F		> 18 y	46,XX,r(13)					
2	RC13-16						6974524	Two cases of ring chromosome 13. Chromosome banding patterns and mosaic configuration	Steinbach P, Drews K, Horstmann W, Barbi G, Scholz W.	Ann Genet. 1981;24(3):152-7.	1981
	RC13-17						7279505	[Children with ring chromosome 13]	Bliumina MG, Demintseva VS.	Pediatriia. 1981 May;(5):51-2.	1981
1	RC13-18						7242723	[A patient with ring chromosome 13]	Hammond A, Bijlsma JB.	Ned Tijdschr Geneeskd. 1981 May	1981
1	RC13-19	nb	M			46,XY,r(13)(p12q22)	7332149	[Ring chromosome 13 and multiple malformations (author's transl)]	Antich J, Plaza J, Geán E.	An Esp Pediatr. 1981 Nov;15(5):469-73.	1981
1	RC13-20 case 1	nb	F			46,XX,r(13)[0.88]	7129419	The ring chromosome 13 syndrome	Martin NJ, Harvey PJ, Pearn JH.	Hum Genet. 1982;61(1):18-23. doi:	1982
1	RC13-20 case 2	nb	F			46,XX,r(13)[0.88]	7129419				
1	RC13-20 case 3	3.5 y	F			46,XX,r(13)[0.89]	7129419				
1	RC13-21						6604485	Ring 13 in an adult male with a 13:13 translocation mother	de Almeida JC, Llerena JC Jr, Gomes DM, Martins RR, Pereira ET.	Ann Genet. 1983;26(2):112-5.	1983
1	RC13-22	pn	M	de novo	top	46,XY,r(13)(p11q13)	6657600	A fetus with a chromosome 13 ring and placenta with chromosome 13 rod/ring mosaicism	Benn A, Warburton D, Byrne JM, Rudelli R, Shonhaut A, Yeboa K,	Prenat Diagn. 1983 Oct;3(4):297-302. doi: 10.1002/pd.1970030406.	1983
1	RC13-23	2.5 y	M	de novo		46,XY,r(13)(p12q31)	4076259	Clinical features in a case with ring chromosome 13	Parcheta B, Wisniewski L, Piontek E, Szymanska J, Skawinski W, Wermenski K.	Eur J Pediatr. 1985 Nov;144(4):409-12. doi: 10.1007/BF00441791.	1985
1	RC13-24	3 m	F			46,XX,r(13)[0.94]	3806666	Tissue-specific mosaicism for the stability of a ring 13 chromosome	McCorquodale MM, Kolacki P, Kurczynski TW, Baugh E.	J Ment Defic Res. 1986 Dec;30 (Pt 4):389-99. doi: 10.1111/j.1365-2788.1986.tb01334.x.	1986
1	RC13-25						3504037	[Retinoblastoma: first case with a ring chromosome 13 in black Africa]	Diallo JS, Afoutou JM, Balo K.	Rev Int Trach Pathol Ocul Trop Subtrop Sante Publique. 1987;(64):183-6.	1987
1	RC13-26						3613828	[Ring chromosome 13 (case report)]	Zergollern L, Barisić I.	Lijec Vjesn. 1987 Apr-May;109(4-5):146-9.	1987
1	RC13-27						2221637	[Ring chromosome 13 (type I) 45,XY,-13/46, XY,r(13)(p11:q34)]	Díaz-Cardama Sousa I, Mora Gandarillas I, Vázquez Rodríguez M, Viso Lorenzo A, Alonso Villa MJ, Fernández Toral J.	An Esp Pediatr. 1990 Jun;32(6):556-8.	1990
1	RC13-28						2077349	Evidence for involvement of a Robertsonian translocation 13 chromosome in formation of a ring chromosome 13	Stetten G, Tuck-Muller CM, Blakemore KJ, Wong C, Kazazian HH Jr, Antonarakis SE.	Mol Biol Med. 1990 Dec;7(6):479-84.	1990
1	RC13-29	pn, 29 gwk	M		stillbirth	46,XY,r(13)(p11q21.1)	1433229	Ring chromosome 13: lack of distinct syndromes based on different breakpoints on 13q	Brandt CA, Hertz JM, Petersen MB, Vogel F, Noer H, Mikkelsen M.	J Med Genet. 1992 Oct;29(10):704-8. doi: 10.1136/jmg.29.10.704.	1992
1	RC13-30	5 y	M			46,XY[0.35]/46,XY,r(13)[0.61]/46.XY,dic r(13)[0.04]	1363214	Mosaic ring chromosome 13 analyzed by fluorescence in situ hybridization: report of a case	Hou JW, Liu CH, Wang TR, Zhu HM, Jiang S, Sciorra LJ, Lee ML.	J Formos Med Assoc. 1992 Nov;91(11):1108-11.	1992
1	RC13-31	pn, 20 gwks	M	de novo	stillbirth	46,XY,r(13)[5]/45,XY,-13[15]	8260080	Ultrasonographic prenatal diagnosis of the 13q-syndrome	Santolaya J, McCorquodale MM, Torres W, Meyer WJ, Gauthier D, Lemery D.	Fetal Diagn Ther. 1993 Jul-Aug;8(4):261-7. doi: 10.1159/000263837.	1993
1	RC13-32						8086666	Use of chromosome painting for marker chromosome identification in two children with congenital disorders	Doco-Fenzy M, Navrocki B, Cornillet P, Sabouraud P, Robillard P, Gruson N, Gaillard D, Adnet JJ	Bull Assoc Anat (Nancy). 1994 Jun;78(241):9-13. PMID: 8086666.	1994

1	RC13-33	nb	M		d. 3 y heart failure	46,XY,r(13)(p11q32)	9332662	Ring chromosome 13 with loss of the region D13S317-D13S285: phenotypic overlap with XK syndrome	Guala A, Dellavecchia C, Mannarino S, Rognone F, Giglio S, Minelli A, Danesino C.	Am J Med Genet. 1997 Oct 31;72(3):319-23.	1997
1	RC13-34						9664214	Skin pigmentation anomalies in ring chromosome 13	Fryns JP, Devriendt K, Legius E.	Genet Couns. 1998;9(2):155-6.	1998
1	RC13-35	3 m	M	de novo		46,XY,r(13)(p11q32)/45,XY,-13	9823499	Ring chromosome 13 in an infant with multiple congenital anomalies and penoscrotal transposition	Boduroglu K, Alikaşifoglu M, Tunçbilek E, Uludogan S.	Clin Dysmorphol. 1998 Oct;7(4):299-301. doi: 10.1097/00019605-199810000-00012.	1998
1	RC13-36	nb	F			46,XX,r(13)	9881566	Retinal detachment in an infant with the ring chromosome 13 syndrome	Filous A, Rasková D, Kodet R.	Acta Ophthalmol Scand. 1998 Dec;76(6):739-41. doi: 10.1034/j.1600-0420.1998.760624.x.	1998
1	RC13-37	1.5 y	F	de novo		46,XX,r(13)(p11q14)/46,XX,der(13)	9950374	Molecular and cytogenetic characterisation of an unusual case of partial trisomy/partial monosomy 13 mosaicism: 46,XX,r(13)(p11q14)/46,XX,der(13)t(13;13)(q10;q1	Gentile M, Buonadonna AL, Cariola F, Fiorente P, Valenzano MC, Guanti G.	J Med Genet. 1999 Jan;36(1):77-82.	1999
1	RC13-38	2.5 y	M			46.XY,r(13)	10951464	Boy with celiac disease, malformations, and ring chromosome 13 with deletion 13q32-->qter	Talvik I, Ounap K, Bartsch O, Ilus T, Uibo O, Talvik T.	Am J Med Genet. 2000 Aug 28;93(5):399-402.	2000
1	RC13-39	8 y	F		> 19y	46,XX,r(13)	11032444	Keratoconus associated with chromosome 13 ring abnormality	Heaven CJ, Lalloo F, Mchale E.	Br J Ophthalmol. 2000 Sep;84(9):1079. doi: 10.1136/bjo.84.9.1075d.	2000
1	RC13-40						11412482	[Ring 13 chromosome]	Moreno García M.	An Esp Pediatr. 2001 Jul;55(1):95.	2001
1	RC13-41	pn	F	de novo	top	46,XX,r(13)(p11q32)[0.77]/45,XX,-13[0.23]	11241535	Prenatal diagnosis of mosaic ring chromosome 13 with anencephaly	Chen CP, Chern SR, Lee CC, Chen WL, Wang W.	Prenat Diagn. 2001 Feb;21(2):102-5. doi: 10.1002/1097-	2001
1	RC13-42	nb	M			47,XY,+r(13)	11170089	Boy with bilateral retinoblastoma due to an unusual ring chromosome 13 with activation of a latent centromere	Morrissette JD, Celle L, Owens NL, Shields CL, Zackai EH, Spinner NB.	Am J Med Genet. 2001 Feb 15;99(1):21-8. doi: 10.1002/1096-8628(20010215)99:1<21::aid-	2001
1	RC13-43						11273829	[Ring chromosome 13 and congenital coagulation factor deficiency]	Ruiz Del Prado M, Alfonso Landa J, Cristóbal Navas C, Blázquez Regidor J,	An Esp Pediatr. 2001 Apr;54(4):411-2.	2001
1	RC13-44	nb	M			46,XY,r(13)(p11;q34)	11590457	Ring chromosome 13 in an Omani infant boy with mental retardation and multiple congenital anomalies	Venugopalan P, Kenue RK.	Saudi Med J. 2001 Sep;22(9):800-3.	2001
1	RC13-45						15300643	[Chromosome analysis and phenotype location analysis on a patient with the karyotype of 45, XX, -13/46, XX, r(13)/46, XX, r(13;13)/47, XX, 2r(13)(p13q32.3)]	Liang DS, Wu LQ, Long ZG, Pan Q, Dai HP, Xia JH.	Zhonghua Yi Xue Yi Chuan Xue Za Zhi. 2004 Aug;21(4):392-4.	2004
1	RC13-46	4.5 y	F	mat	> 21 y mat	46,XX,r(13)(p13q34)	15326636	Transmission of ring chromosome 13 from a mother to daughter with both having a 46,XX, r(13)(p13q34) karyotype	Bedoyan JK, Flore LA, Alkatib A, Ebrahim SA, Bawle EV.	Am J Med Genet A. 2004 Sep 1;129A(3):316-20. doi: 10.1002/ajmg.a.30242.	2004
1	RC13-47	4 m	M	de novo		46,XY,r(13)(p11q34)	16585823	Ring chromosome 13 in an infant with ambiguous genitalia	Sankar VH, Phadke SR.	Indian Pediatr. 2006 Mar;43(3):258-60.	2006
1	RC13-48	nb	M	de novo		46,XY,r(13)(p11.1q33)	18233171	Chromosome deletions in 13q33-34: report of four patients and review of the literature	Walczak-Sztulpa J, Wisniewska M, Latos-Bielenska A, Linné M, Kelbova C, Belitz B, Pfeiffer L, Kalscheuer V, Erdogan F, Kuss AW, Ropers HH,	Am J Med Genet A. 2008 Feb 1;146A(3):337-42. doi: 10.1002/ajmg.a.32127. PMID: 18203171.	2008
1	RC13-49	10 m	M	de novo		46,XYY,r(13)(p11q34)	21396087	Ring chromosome 13 syndrome characterized by high resolution array based comparative genomic hybridization in patient with 47, XYY syndrome: a case report	Liao C, Fu F, Zhang L.	J Med Case Rep. 2011 Mar 11;5:99. doi: 10.1186/1752-1947-5-99.	2011
1	RC13-50						23431756	A case of ring chromosome 13 with ambiguous genitalia and primary hypothyroidism	Sandal G, Caliskan D, Ormeci AR, Oztas S.	Genet Couns. 2012;23(4):529-32.	2012
1	RC13-51	nb	M	de novo	d. 2m	46,XY,r(13)(p11.2q34)[51]/45,XY,-13[12]/46,Xy,dic r(13)[1]	24052730	Ring autosomes: some unexpected findings	Caba L, Rusu C, Plăiaşu 5th, Gug G, Grămescu M, Bujoran C, Ochiană D, Voloşciuc M, Popescu R, Braha E,	Balkan J Med Genet. 2012 Dec;15(2):35-46. doi: 10.2478/bjmg-2013-0005.	2012
1	RC13-52						24032285	Anal atresia, abnormal genitalia, and absent thumb: congenital malformations associated with mosaic ring chromosome 13	Ocak Z, Ozlu T, Vural M.	Genet Couns. 2013;24(2):157-60.	2013
1	RC13-53 case 1	15 y	M			46,XY,r(13)[40]/45,XY,-13[10]	24032290	Clinical, cytogenetic and molecular characterization of two cases of mosaic ring	Uwineza A, Pierquin G, Gaillez S, Jamar M, Hellin AC, Caberg JH, Bours V.	Genet Couns. 2013;24(2):193-200.	2013
1	RC13-53 case 2	pn	M		top	46.XY,r(13)(q32q34)[18]/46,XY,dic r(13)[2]	24032290				
1	RC13-54	6 y	F			46,XX,r(13)(p13q34)[71]/45,XX,-13[12]/r-var[17]	23661454	Smallest critical region for microcephaly in a patient with mosaic ring chromosome 13	Su PH, Chen CP, Su YN, Chen SJ, Lin LL, Chen JY.	Genet Mol Res. 2013 Apr 25;12(2):1311-7. doi: 10.4238/2013.April.25.2.	2013

1	RC13-55	pn	M	de novo	top	46,XY,r(13)[33]/45,XY,-13[19]	23933417	Prenatal diagnosis and molecular cytogenetic characterization of mosaic ring chromosome 13	Chen CP, Tsai CH, Chern SR, Wu PS, Su JW, Lee CC, Chen YT, Chen WL, Chen LF, Wang W.	Gene. 2013 Oct 15;529(1):163-8. doi: 10.1016/j.gene.2013.07.050. Epub 2013 Aug 8.	2013
1	RC13-56 case 1	3 y	M	de novo		46,XY,r(13)(p11q34)	27625853	Molecular cytogenetic and phenotypic characterization of ring chromosome 13 in three unrelated patients	Abdallah-Bouhjar IB, Mougou-Zerelli S, Hannachi H, Gmidène A, Labalme A, Soyah N, Sanlaville D, Saad A, Elghezal H.	J Pediatr Genet. 2013 Sep;2(3):147-55. doi: 10.3233/PGE-13063.	2013
1	RC13-56 case 2	4 y	F	de novo		46,XX,r(13)(p11q34)	27625853				
1	RC13-56 case 3	3 y	M	de novo		46,XY,r(13)(p11q34)	27625853				
1	RC13-57	11 m	M			46,XY,r(13)(p11q34)[0.83]/45,XY,-13[0.17]	24932608	Ring chromosome 13 and ambiguous genitalia	Ozsu E, Yeşiltepe Mutlu G, Ipekçi B.	J Clin Res Pediatr Endocrinol. 2014;6(2):122-4. doi: 10.4274/Jcrpe.1194.	2014
1	RC13-58	3.5 y	M	de novo		46,XY,r(13)(p11.2q34)	25171325	A case with a ring chromosome 13 in a cohort of 203 children with non-syndromic autism and review of the cytogenetic literature	Charalsawadi C, Maisrikhaw W, Praphanphoj V, Wirojanan J, Hansakunachai T, Roongpraiwan R, Sombuntham T, Ruangdaraganon N, Limprasert P.	Cytogenet Genome Res. 2014;144(1):1-8. doi: 10.1159/000365909. Epub 2014 Aug 23.	2014
1	RC13-59	nb	M	de novo		46,XY,r(13)(p13q34)[89]/45,XY,-13[5]/46,XY,r(13;13)[7]	25377780	Molecular and cytogenetic evaluation of a patient with ring chromosome 13 and discordant results	Kaylor J, Alfaro M, Ishwar A, Sailey C, Sawyer J, Zarate YA.	Cytogenet Genome Res. 2014;144(2):104-8. doi: 10.1159/000368649. Epub 2014 Nov 6.	2014
1	RC13-60	nb	F			46,XX,r(13)[77]/45,XX,-13[17]/46,XX,idic r(13)[6]	25867311	Postnatal diagnosis of constitutive ring chromosome 13 using both conventional and molecular cytogenetic approaches	Minasi LB, Pinto IP, de Almeida JG, de Melo AV, Cunha DM, Ribeiro CL, Silva GP, Brasil MG, Silva DM, da Silva CC, da Cruz AD.	Genet Mol Res. 2015 Mar 6;14(1):1692-9. doi: 10.4238/2015.March.6.15.	2015
1	RC13-61 case 1	6 m	F	de novo		47,XX,r(13)(p13q34),+mar	29518772	Two Cases with Ring Chromosome 13 at either End of the Phenotypic Spectrum	Çakmaklı S, Çankaya T, Gürsoy S, Koç A, Kırbıyık Ö, Kılıçarslan ÖA, Özer E, Erçal	Cytogenet Genome Res. 2017;153(4):175-180. doi:	2017
1	RC13-61 case 2	nb	F	de novo	d. 24 hr	46,XX,r(13)(q11q24)	29518772				
1	RC13-62	pn	M	de novo	top	46,XY,r(13)[114]/45,XY,-13[3]	29237938	Molecular and Cytogenetic Characterization of a Fetus with Mosaic Ring Chromosome 13: A Very	Zhao XR, Han X, Wang YL, Hu WJ.	Chin Med J (Engl). 2017 Dec 20;130(24):3007-3008. doi:	2017
1	RC13-63	5 m	F	de novo		46,XX,r(13)(p11q33)[82]/45,XX,-13[10]/46,XX,r(13;13)(p11q33;p11q33)[8]	29972124	[Clinical and genetic features of ring chromosome 13 syndrome: an analysis of one case]	Fan MR, Wang GJ, Yu XY.	Zhongguo Dang Dai Er Ke Za Zhi. 2018 Jun;20(6):485-489. doi: 10.7499/j.issn.1008-8830.2018.06.011.	2018
1	RC13-64	17 y	M			SF: 46,XY,r(13)[19]/45,XY,-13[2]/46,XY,-13,+mar[1]	30500678	Induced pluripotent stem cell line, IMGTi003-A, derived from skin fibroblasts of an intellectually disabled patient with ring chromosome 13	Nikitina TV, Menzorov AG, Kashevarova AA, Gridina MM, Khabarova AA, Yakovleva YS, Lopatkina ME, Pristyazhnyuk IE, Vasilyev SA, Serov OL, Lebedev IN.	Stem Cell Res. 2018 Dec;33:260-264. doi: 10.1016/j.scr.2018.11.009. Epub 2018 Nov 20.	2018
1	RC13-65	? 20 y	F		> 20 y	46,XX,r(13)(p13q34)[84]/45,XX,-13[9]/46,XX,r(13;13)[5]	30512167	[Genetic analysis of a patient with premature ovarian failure and a 45,XX,-13/46,XX,r(13)(p13q34)/46,XX,r(13;13) karyotype]	Yin T, Zheng A, Tan J, Zhang R, Gu Y, Wang L.	Zhonghua Yi Xue Yi Chuan Xue Za Zhi. 2018 Dec 10;35(6):872-874. doi: 10.3760/cma.j.issn.1003-9406.2018.06.023.	2018
1	RC13-66	8 m	M			46,XY,r(13)	30907385	Cromosoma 13 en anillo	Cammarata-Scalisi F, Briceño Y, Cegarra E, Montilla D.	Bol Med Hosp Infant Mex. 2019;76(2):100-103. doi: 10.24875/BMHIM.18000108.	2019
1	RC13-67 case 1	nb	F	de novo		46,XX,r(13)[421/500]	31976095	Towards New Approaches to Evaluate Dynamic Mosaicism in Ring Chromosome 13 Syndrome	Petter C, Moreira LMA, Riegel M.	Case Rep Genet. 2019 Dec 28;2019:7250838. doi: 10.1155/2019/7250838. eCollection 2019.	2019
1	RC13-67 case 2	nb	M	de novo		46,XY,r(13)[429/500]	31976095				
1	RC13-67 case 3	nb	F	de novo		46,XX,r(13)[421/500]	31976095				
1	RC13-68	pn	M	de novo	top	46,XY,r(13)[18]/45,XY,-13[8]/46,XY,-13,+mar[14]	32039781	Prenatal diagnosis and molecular cytogenetic characterization of mosaicism for r(13), monosomy 13 and idic r(13) by amniocentesis	Chen CP, Chen CY, Chern SR, Wu PS, Chen SW, Lee CC, Chen LF, Wang W.	Taiwan J Obstet Gynecol. 2020 Jan;59(1):130-134. doi: 10.1016/j.tjog.2019.11.021.	2020
1	RC13-69	pn	F	de novo	top	46,XX,r(13)[12]/45,XX,-13[8]	34247823	Prenatal diagnosis of recurrent mosaic ring chromosome 13 of maternal origin	Chen CP, Chen CY, Chern SR,	Taiwan J Obstet Gynecol. 2021 Jul;60(4):771-774	2021
1	RC13-70	pn	F	de novo	top	46,XX,r(13)(p11.1q31)[40]/45,XX,-13[10]	33966748	Prenatal diagnosis of a fetus with mosaic ring chromosome 13: Case report and review of the literature	Hu XN, Li LL, Shi QY	Taiwan J Obstet Gynecol. 2021 May;60(3):554-558	2021
1	RC13-71	? 3 y	M			46,XY,r(13)(p11.1q34)	34140436	A Case Study of Ring Chromosome 13 in a Pediatric Patient	Okabe A, Palencia D, Trejo-Solis D, Duarte-Martinez A	J Assoc Genet Technol. 2021;47(2):75-77	2021
1	RC13-72	3 m	F		> 34 y	BL (3 y): 46,XX<br>SF(4 y): 46,XX/46,XX,r(13)	33438813	Lessons from a 30 year follow-up of monozygotic twins with discordant phenotype due to a ring 13 chromosomal mosaicism in one of them	Chanes B, Arriaza M, Lacassie Y	Am J Med Genet A. 2021 Apr;185(4):1242-1246	2021
**82**	**72**										

1	RC14-1, case O	8 y	M			46,XY,r(14)	1056013	Somatic rearrangement of chromosome 14 in human lymphocytes	McCaw BK, Hecht F, Harnden DG, Teplitz RL.	Proc Natl Acad Sci U S A. 1975 Jun;72(6):2071-5. doi: 10.1073/pnas.72.6.2071.	1975
1	RC14-2						302677	[Ring chromosome 14 in monozygotic twins]	Jalbert P, Sele B, Jalbert H, Sirand L,	Ann Genet. 1977 Mar;20(1):59-62.	1977
1	RC14-3						305756	Extended evaluation of previously reported twins with a ring 14 chromosome	Sparkes RS, Klisak I, Sparkes MC.	Ann Genet. 1977 Dec;20(4):273-5.	1977
1	RC14-4						614989	Multiple congenital malformations in a child with ring 14 chromosome	Dzarlieva R.	God Zb Med Fak Skopje. 1977;23:285-91.	1977
1	RC14-5	8 m	F	mat nl		46,XX,r(14)	7211367	Ring chromosome 14 in a mentally retarded girl	Iselius L, Ritzén M, Bui TH, Olsson K, Eklöf O.	Acta Paediatr Scand. 1980 Nov;69(6):803-6. doi: 10.1111/j.1651-2227.1980.tb07157.x.	1980
1	RC14-6	nb	F	de novo		46,XX,r(14)(p11q32)	6156115	A ring 14 chromosome with deleted short arm	Amarose AP, Dorus E, Huttenlocher PR,	Hum Genet. 1980;54(2):145-7. doi:	1980
1	RC14-7	8 m	F	de novo		46,XX,r(14)(p12q24)	7277427	Ring chromosome 14: a distinct clinical entity	Schmidt R, Eviatar L, Nitowsky HM, Wong M, Miranda S.	J Med Genet. 1981 Aug;18(4):304-7. doi: 10.1136/jmg.18.4.304.	1981
1	RC14-8	nb	M			46,XY,r(14)	6977306	Infant male with ring chromosome 14	Triolo O, Serra A, Bova R, Carlo Stella N, Caruso P.	Ann Genet. 1981;24(4):236-8.	1981
1	RC14-9	2.5 y	F			46,XX,r(14)	7241544	Inheritance of a ring 14 chromosome	Riley SB, Buckton KE, Ratcliffe SG, Syme J.	J Med Genet. 1981 Jun;18(3):209-13. doi: 10.1136/jmg.18.3.209.	1981
1	RC14-10	7 m	M	de novo		46,XY,r(14)	6170224	Ring 14 chromosome: association with seizures	Lippe BM, Sparkes RS.	Am J Med Genet. 1981;9(4):301-5. doi: 10.1002/ajmg.1320090406.	1981
1	RC14-11	12.5 y	F			46,XX,r(14)	6982671	Ring chromosome 14 syndrome	Fryns JP, Petit P, Kleczkowska A, De Muelenaere A, ven den Berghe H.	Ann Genet. 1982;25(3):179-80.	1982
2	RC14-12						6674412	Ring chromosome 14. A distinct clinical entity	Fryns JP, Kubien E, Kleczkowska A, Nawrocka-Kanska B, Van den Berghe H.	J Genet Hum. 1983 Dec;31 Suppl 5:367-75.	1983
1	RC14-13						6411044	[Ocular anomalies in phenotype 46,XY,r(14) (ring chromosome 14)]	Guillot M, Dufier JL, Perignon F, Lenoir G, de Grouchy J, Pinaudeau Y.	Arch Fr Pediatr. 1983 May;40(5):433.	1983
1	RC14-14	2.5 y	F			45,XX,r(14)[89/100]	6702898	Ring chromosome 14 and immunoglobulin locus	Krawczun M, Melink G, Cervenka J.	Am J Med Genet. 1984 Feb;17(2):465-9. doi: 10.1002/ajmg.1320170209.	1984
1	RC14-15						6609671	Ring-14 and trisomy 14q in the same child	Pangalos C, Velissariou V, Ghica M,	Ann Genet. 1984;27(1):38-40.	1984
1	RC14-16						6331796	[Ring chromosome 14. II. A case report of r(14) mosaicism. The r(14) phenotype]	Rethoré MO, Caille B, Huet de Barochez Y, de Blois MC, Ravel A,	Ann Genet. 1984;27(2):91-5.	1984
1	RC14-17						6331795	[Ring chromosome 14. I. A case report on homogeneous r(14)]	Raoul O, Razavi F, Lescs MC, Bouhanna A.	Ann Genet. 1984;27(2):88-90.	1984
2	RC14-18						4026139	[2 new cases of ring chromosome 14]	Caille B, Rethoré MO, Raoul O, Huet de Barochez Y, Dufier JL, Roy C, Harpey JP,	Ann Pediatr (Paris). 1985 May;32(5):441-6.	1985
1	RC14-19						3477901	Ocular findings in a patient with deletion short arm chromosome 5 (cri du chat) and ring	Clark DI, Howard PJ, Patterson A.	Trans Ophthalmol Soc U K. 1986;105 (Pt 6):723-5.	1986
1	RC14-20						3620197	Ring chromosome 14 without deletion	Angelova E, Mitreva B, Toncheva D.	Acta Paediatr Hung. 1987;28(1):59-62.	1987
1	RC14-21	3 y	F	de novo		46,XX,r(14)	3169736	Retinal/macular pigmentation in conjunction with ring 14 chromosome	Howard PJ, Clark D, Dearlove J.	Hum Genet. 1988 Oct;80(2):140-2. doi: 10.1007/BF00702856.	1988
1	RC14-22	4.5 y	M			46,XY,r(14)	2597013	[Epilepsy in ring chromosome 14 syndrome]	Duarte MH, Lison MP, Ferrari I, Soares LR, Fernandes RM.	Arq Neuropsiquiatr. 1989 Jun;47(2):205-11. doi: 10.1590/s0004-282x1989000200013.	1989
0	RC14-23						2543620	Molecular characterization of a ring chromosome 14 showing that the PI locus is centromeric to the D14S1 and IGH loci	Keyeux G, Gilgenkrantz S, Lefranc G, Lefranc MP.	Hum Genet. 1989 Jun;82(3):219-22. doi: 10.1007/BF00291158.	1989
1	RC14-24	6 m	M	mat		46,XY,r(14)	2202211	Transmission of ring 14 chromosome from mother to two sons	Matalon R, Supple P, Wyandt H, Rosenthal IM.	Am J Med Genet. 1990 Aug;36(4):381-5. doi: 10.1002/ajmg.1320360402.	1990
0	RC14-24 mat	22 y	F		> 22 y	46,XX,r(14)[16]/45,XX,t(14q21q)[9]	2202211				
1	RC14-25		M			46,XY,r(14)	2303262	Physical mapping of probes within 14q32, a subtelomeric region showing a high recombination frequency	Hofker MH, Smith S, Nakamura Y, Teshima I, White R, Cox DW.	Genomics. 1990 Jan;6(1):33-8. doi: 10.1016/0888-7543(90)90445-z.	1990
1	RC14-26						2288460	[r14 syndrome without major dysmorphism]	de Blois MC, Caille B, Rethoré MO, Dufier JL, Lejeune J.	Ann Genet. 1990;33(3):155-8.	1990
2	RC14-27						1746891	Ring chromosome 14 syndrome. Report of two cases, including extended evaluation of a previously reported patient and review	Zelante L, Torricelli F, Calvano S, Mingarelli R, Dallapiccola B.	Ann Genet. 1991;34(2):93-7.	1991
2	RC14-28						1462428	[The ring chromosome 14 syndrome]	Kristensen I, Wieg C, Friedrich UK.	Ugeskr Laeger. 1992 Nov 9;154(46):3248-9.	1992
1	RC14-29						1381073	[Ring chromosome 14]	Rudenskaia GE, Pozdniakova EO,	Pediatriia. 1992;(3):66-9.	1992

1	RC14-30	6 m	F	de novo		46,XX,r(14)(p13q32.3)	1443409	Ring 14 chromosome with complex partial seizures: a case report	Shirasaka Y, Ito M, Okuno T, Fujii T, Nozaki K, Mikawa H.	Brain Dev. 1992 Jul;14(4):257-60. doi: 10.1016/s0387-7604(12)80243-9.	1992
1	RC14-31	5 y	F			46,XX,r(14)	8502366	[Epilepsy in a child with ring chromosome 14]	Midro AT, Zadrozna-Tołwińska B.	Neurol Neurochir Pol. 1993 Jan-	1993
1	RC14-32	9 m	M	de novo		46,XY,r(14)(p13q32.33)	7759068	Molecular analysis redefines three human chromosome 14 deletions	Wintle RF, Costa T, Haslam RH, Teshima IE, Cox DW.	Hum Genet. 1995 May;95(5):495-500. doi: 10.1007/BF00223859.	1995
1	RC14-33	pn 12 gwk	F	de novo	top	46,XX,r(14)/45,XX,-14	8986702	Prenatal diagnosis of ring chromosome 14 after intracytoplasmic sperm injection	Jean M, Rival JM, Mensier A, Mirallié S, Lopes P, Barrière P.	Fertil Steril. 1997 Jan;67(1):164-5. doi: 10.1016/s0015-0282(97)81874-2.	1997
1	RC14-34						9662854	[Ring chromosome 14: report of a new case]	Campos Tristán C, Casado Durández P.	An Esp Pediatr. 1998 Jun;48(6):650-	1998
1	RC14-35	1 m	M			46,XY,r(14)(p11.2q32.3)/45,XY-14	10029266	Ring chromosome 14 complicated with complex partial seizures and hypoplastic corpus callosum	Ono J, Nishiike K, Imai K, Otani K, Okada S.	Pediatr Neurol. 1999 Jan;20(1):70-2. doi: 10.1016/s0887-8994(98)00099-x.	1999
1	RC14-36	29 y	F		> 29 y		10714162	[A case of ring 14 chromosome with ocular manifestations]	Hisatomi T, Kira R, Sakamoto T, Inomata H.	Nippon Ganka Gakkai Zasshi. 2000 Feb;104(2):121-4.	2000
1	RC14-37 pt1	3 m	M			46,XY,r(14)(p12q32.33)/45,XY,-14	12919399	Ring chromosome 14 with localization-related epilepsy: three cases	Morimoto M, Usuku T, Tanaka M, Otabe O, Nishimura A, Ochi M,	Epilepsia. 2003 Sep;44(9):1245-9. doi: 10.1046/j.1528-	2003
1	RC14-37 pt2	7 m	M	de novo		46,XY,r(14)(p11.2q32.33)	12919399				
1	RC14-37 pt3	9 m	M			46,XY,r(14)(p11.2q32.31)	12919399				
1	RC14-38	1.5 y	F	de novo		46,XX,r(14)[39]/45,XX,-14[11]	15733381	[Ring chromosome 14 syndrome: a case report]	Li R, Zhao ZY, Sun LY, Zheng X.	Zhonghua Er Ke Za Zhi. 2004 Dec;42(12):956-7.	2004
1	RC14-39	1 y	M			46,XY,r(14)(p11.2q32.31)[84]/45,XY,-14[10]/46,XY,dic r(14)[6]	15366814	Mosaic ring chromosome 14 and monosomy 14 presenting with growth retardation, epilepsy, and blepharophimosis	Hou JW.	Chang Gung Med J. 2004 May;27(5):373-8.	2004
1	RC14-40 HSC27804	7 m	F			46,XX,r(14)(p11.2q32.3)	16152642	FISH-mapping of telomeric 14q32 deletions: search for the cause of seizures	Schlade-Bartusiak K, Costa T, Summers AM, Nowaczyk MJ, Cox DW.	Am J Med Genet A. 2005 Oct 15;138A(3):218-24. doi:	2005
1	RC14-40 HSC1313	7 m	F			46,XX,r(14)(p11q32)	16152642				
1	RC14-40 HSC1412	1 y	M		d. 13 y pneumonia	46,XY,r(14)(p11q32)	16152642				
1	RC14-40 CC0066	n/a	M			46,XY,r(14)	16152642				
1	RC14-41	1.5 y	F			46,XX,r(14)(p11.2q32.3)	16951445	Ring chromosome 14 with epilepsy and development delay	Sheth FJ, Soni N.	Indian Pediatr. 2006 Aug;43(8):744-5.	2006
1	RC14-42	2 y	M			46,XY,r(14)(p11qter)[72]/46,XY,idic r(14)[18]	17715281	Mosaic trisomy r(14) associated with epilepsy and mental retardation	Tzoufi M, Kanioglou C, Dasoula A, Asproudis I, Tsatsoulis A, Sismani C,	J Child Neurol. 2007 Jul;22(7):869-73. doi:	2007
1	RC14-43	4 m	F	de novo		46,XX,r(14)[0.95]	17342151	Ring chromosome formation as a novel escape mechanism in patients with inverted duplication and terminal deletion	Knijnenburg J, van Haeringen A, Hansson KB, Lankester A, Smit MJ, Belfroid RD, Bakker E, Rosenberg C,	Eur J Hum Genet. 2007 May;15(5):548-55. doi: 10.1038/sj.ejhg.5201807. Epub	2007
1	RC14-44	16 y	M			46,XY,der(16)der(14)r(14;14)[38]/45,XY,der(16)[22]	18414512	Position effect leading to haploinsufficiency in a mosaic ring chromosome 14 in a boy with autism	Castermans D, Thienpont B, Volders K, Crepel A, Vermeesch JR, Schrander-Stumpel CT, Van de Ven WJ, Steyaert	Eur J Hum Genet. 2008 Oct;16(10):1187-92. doi: 10.1038/ejhg.2008.71. Epub 2008	2008
1	RC14-45	pn	F	de novo	top	46,XX,r(14)(p11q32.2)[7]/45,XX,-14[7]	18186139	Prenatal diagnosis of a ring chromosome 14 in a fetus with a severe skeletal dysplasia	Quenum-Miraillet G, Malan V, Martinovic J, Encha-Razavi F, Aral B, Texier I, Bonnefont JP, Vekemans M,	Prenat Diagn. 2008 Jan;28(1):69-71. doi: 10.1002/pd.1911.	2008
1	RC14-46 case 1	4 m	F			46,XX,r(14)(p13q32)	19416318	Ring 14 chromosome presenting as early-onset isolated partial epilepsy	Ville D, DE Bellescize J, Nguyen MA, Testard H, Gautier A, Perrier J, Till M,	Dev Med Child Neurol. 2009 Nov;51(11):917-22. doi:	2009
1	RC14-46 case 2	3 m	M			46,XY,r(14)[86]/45,XY,-14[14],	19416318				
1	RC14-46 case 3	6 m	M			46,XY,r(14)	19416318				
1	RC14-46 case 4	4 m	F			46,XX,r(14)	19416318				
1	RC14-47	2 y	M	de novo	> 30 y	46,XY,r(14)(p13q32.33)[0.96]	20979193	Cytogenetic and molecular evaluation and 20-year follow-up of a patient with ring chromosome 14	Guilherme RS, de Freitas Ayres Meloni V, Sodré CP, Christofolini DM, Pellegrino R, de Mello CB, Conlin LK, Hutchinson AL, Spinner NB, Brunoni D,	Am J Med Genet A. 2010 Nov;152A(11):2865-9. doi: 10.1002/ajmg.a.33689.	2010
1	RC14-48	1 y	M			46,XY,r(14)([80]/45,XY,-14[20]	20643614	Partial epilepsy complicated by convulsive and nonconvulsive episodes of status epilepticus in a patient with ring chromosome 14 syndrome	Giovannini S, Frattini D, Scarano A, Fusco C, Bertani G, Della Giustina E, Martinelli P, Orteschi D, Zollino M, Neri	Epileptic Disord. 2010 Sep;12(3):222-7. doi: 10.1684/epd.2010.0324. Epub 2010	2010
1	RC14-49	6 m	M			46,XY,r(14)(p13q32)[48]/46,XY,dup r(14)[2]	20034090	Ring chromosome 14 mosaicism: an unusual case associated with developmental delay and epilepsy, characterized by genome array-CGH	Nucaro AL, Falchi M, Pisano T, Rossino R, Boscarelli F, Stoico G, Milia A, Montaldo C, Cianchetti C, Pruna D.	Am J Med Genet A. 2010 Jan;152A(1):234-6. doi: 10.1002/ajmg.a.33167.	2010
1	RC14-50 case 1	8 m	F			46,XX,r(14)(p11q32.33)	23159383	Epilepsy in ring 14 chromosome syndrome	Specchio N, Trivisano M, Serino D, Cappelletti S, Carotenuto A, Claps D, Marras CE, Fusco L, Elia M, Vigevano F.	Epilepsy Behav. 2012 Dec;25(4):585-92. doi: 10.1016/j.yebeh.2012.09.032. Epub	2012
1	RC14-50 case 2	9 m	M			46,XY,r(14)(p11q32)[28]/45,XY,-14[4]	23159383				
0	RC14-51						22564756	The ring 14 syndrome	Zollino M, Ponzi E, Gobbi G, Neri G.	Eur J Med Genet. 2012 May;55(5):374-80. doi: 10.1016/j.ejmg.2012.03.009. Epub 2012 Apr 14.	2012

0	RC14-52	nb	M			Not a r(14) case	22488736	A child with an inherited 0.31 Mb microdeletion of chromosome 14q32.33: further delineation of a critical region for the 14q32 deletion syndrome	Holder JL Jr, Lotze TE, Bacino C, Cheung SW.	Am J Med Genet A. 2012 Aug;158A(8):1962-6. doi: 10.1002/ajmg.a.35289. Epub 2012 Apr 9.	2012
1	RC14-53	pn	M	de novo	top	46,XY,r(14)(p11.2q32.33)[27]/45,XY,-14[3]	23198189	Prenatal diagnosis of a fetus with congenital heart defect and ring chromosome 14	Sánchez J, García-Díaz L, Chinchón D, Antiñolo G.	Case Rep Genet. 2012;2012:794075. doi: 10.1155/2012/794075. Epub 2012 Nov 5.	2012
1	RC14-54	3 m	M			46,XY,r(14)	26171345	A Case of Autism with Ring Chromosome 14	Tajeran M, Baghbani F, Hassanzadeh-	Iran J Public Health. 2013	2013
0	RC14-55						24116895	Epilepsy in ring 14 syndrome: a clinical and EEG study of 22 patients	Giovannini S, Marangio L, Fusco C, Scarano A, Frattini D, Della Giustina E,	Epilepsia. 2013 Dec;54(12):2204-13. doi: 10.1111/epi.12393. Epub 2013	2013
1	RC14-56	6 m	F			46,XX,r(14)	24382541	Ring chromosome 14 syndrome presenting with intractable epilepsy: a case report	Incecik F, Hergüner MO, Mert G, Erdem S, Altunbaşak S.	Turk J Pediatr. 2013 Sep-Oct;55(5):549-51.	2013
1	RC14-57	2 y	M			46,XY,r(14)(p13q32.3)[28]/45,XY,-14[2]	24205694	[The effectiveness of lamotrigine in a case of ring chromosome 14 with refractory epilepsy]	Ogawa K, Iyoda K.	No To Hattatsu. 2013 Sep;45(5):379-82.	2013
1	RC14-58	9 m	F			46,XX,r(14)(p11.2q32.3)	23610869	Partial epilepsy and developmental delay in infant with ring chromosome 14	Imataka G, Noguchi M, Tsukada K, Takahashi T, Yamanouchi H, Arisaka O.	Genet Couns. 2013;24(1):81-3.	2013
0	RC14-59						24779649	Developing with ring 14 syndrome: a survey in different countries	Zampini L, Zanchi P, D'Odorico L.	Clin Linguist Phon. 2014 Nov;28(11):844-56. doi:	2014
1	RC14-60	2 m	F		> 18 y	47,XX,del(14)(q21q32.1),+r(14)	25901181	Dysregulation of FOXG1 by ring chromosome 14	Alosi D, Klitten LL, Bak M, Hjalgrim H, Møller RS, Tommerup N.	Mol Cytogenet. 2015 Apr 9;8:24. doi: 10.1186/s13039-015-0129-4. eCollection 2015.	2015
1	RC14-61	nb	F			46,XX,r(14)[0.95]	26773965	Expanding the ocular phenotype of 14q terminal deletions: A novel presentation of microphthalmia and coloboma in ring 14 syndrome with associated 14q32.31 deletion and review of the literature	Salter CG, Baralle D, Collinson MN, Self JE.	Am J Med Genet A. 2016 Apr;170A(4):1017-22. doi: 10.1002/ajmg.a.37436. Epub 2016 Jan 15.	2016
0	RC14-62						26315457	Position effect modifying gene expression in a patient with ring chromosome 14	Guilherme RS, Moysés-Oliveira M, Dantas AG, Meloni VA, Colovati ME,	J Appl Genet. 2016 May;57(2):183-7. doi: 10.1007/s13353-015-0311-8.	2016
1	RC14-63	1 y	M		d. 12 y, multi-organ failure	46,XY,r(14)(p12q32)	28899459	[Ring 14 chromosome syndrome in a boy mainly manifesting as drug-resistant epilepsy]	Chen C, Wu LW, He F, Yang LF, Miao P, Ma YP, Wang XL, Peng J.	Zhongguo Dang Dai Er Ke Za Zhi. 2017 Sep;19(9):949-951. doi: 10.7499/j.issn.1008-	2017
1	RC14-64	7 m	F			46,XX,r(14)(p12q32)	28124115	Developmental trends of communicative skills in children with chromosome 14 aberrations	Zampini L, Zanchi P, Rinaldi B, Novara F, Zuffardi O.	Eur J Pediatr. 2017 Apr;176(4):455-464. doi: 10.1007/s00431-017-2859-2. Epub 2017 Jan 25.	2017
1	RC14-65						28387189	Enamel Pit Defects and Taurodontism in a Patient with Ring Chromosome 14 and 47,XXX	Townsend JA, Lacour L, Scheuerle AE.	J Dent Child (Chic). 2017 Jan 15;84(1):39-43.	2017
1	RC14-66	5 y	F			46,XX,r(14)(p11.1q32.3)	31755799	Multimodal imaging of ring 14 syndrome associated maculopathy	Vasconcelos HM Jr, Vargas ME, Pennesi ME.	Ophthalmic Genet. 2019 Dec;40(6):541-544. doi:	2019
**73**	**66**										
1	RC15-1						4539486	[Ring 15 chromosome: r(15). Identification by controlled denaturation]	Forabosco A, Dutrillaux B, Vassoler G, Lejeune J.	Ann Genet. 1972 Dec;15(4):267-70.	1972
1	RC15-2	2 y	F	de novo		46,XX,r(15)	50276	Ring chromosome 15:r(15). Identification by R banding	Stoll C, Juif JG, Luckel JC, Lausecker C.	Humangenetik. 1975;27(3):259-62. doi: 10.1007/BF00278356.	1975
1	RC15-3	1.5 y	F			46,XX,r(15)	939571	Ring chromosome 15 in child with a minor dysmorphism of phenotype	Rumenić L, Joksimovi I, Anaf M.	Hum Genet. 1976 Jul 27;33(2):187-8. doi: 10.1007/BF00281895.	1976
1	RC15-4						600224	[The r(15) syndrome (ring chromosome 15). Description of a case]	Ferrante E, Boscherini B, Bruni L, Vignetti P, Finocchi G.	Minerva Pediatr. 1977 Nov 17;29(36):2163-8.	1977
1	RC15-5	5 y	F			46,XX,r(15)(p11q26)[95/100]	732019	Ring chromosome 15; 46,XX,r(15) (p11q26) in a girl	Fujita H, Matsumoto H.	Jinrui Idengaku Zasshi. 1978 Sep;23(3):233-7. doi: 10.1007/BF01872473.	1978
1	RC15-6	2 y	F	de novo		46,XX,r(15)	649157	Analysis of banding patterns and mosaic configurations in a case of ring chromosome 15	Schmid M, Henrichs I, Nestler H, Knörr-Gärtner H, Teller WM, Krone W.	Hum Genet. 1978 Apr 24;41(3):289-99. doi: 10.1007/BF00284763.	1978
1	RC15-7						625984	[Ring chromosome 15 in a child (author's transl)]	Scheibenreiter S, Frisch H.	Wien Klin Wochenschr. 1978 Jan 6;90(1):22-5.	1978
1	RC15-8 case 1	3 m	F	de novo		46,XX,r(15)	500090	Ring chromosome 15 syndrome	Fryns JP, Timmermans J, Hondt FD, François B, Emmery L, van den Berghe	Hum Genet. 1979 Sep 2;51(1):43-8. doi: 10.1007/BF00278290.	1979
1	RC15-8 case 2	6.5 y	F	de novo		46,XX,r(15)	500090				
1	RC15-9	2 y	M		> 37 y	46,XY,r(15)[41/45]/45,XY,-15[4/45]	7449181	Ring chromosome 15 in a male adult with radial defects. Evaluation of the phenotype	Meinecke P, Koske-Westphal T.	Clin Genet. 1980 Dec;18(6):428-33. doi: 10.1111/j.1399-0004.1980.tb01788.x.	1980
1	RC15-10	4 m	F	mat nl, pat nd		46,XX,r(15)	7405920	Ring chromosome 15 and failure to thrive	Kousseff BG.	Am J Dis Child. 1980 Aug;134(8):798-9. doi: 10.1001/archpedi.1980.02130200066022.	1980

1	RC15-11	1 y	F	de novo		46,XX,r(15)	6156798	Ring chromosome 15: phenotype, Ag-NOR analysis, secondary aneuploidy, and associated chromosome instability	Ledbetter DH, Riccardi VM, Au WW, Wilson DP, Holmquist GP.	Cytogenet Cell Genet. 1980;27(2-3):111-22. doi: 10.1159/000131472.	1980
1	RC15-12						6929984	A ring 15 chromosome in a girl with minor abnormalities	Gardner RJ, Chewings WE, Holdaway MD.	N Z Med J. 1980 Mar 12;91(655):173-4.	1980
1	RC15-13						7270144	Ring chromosome 15 syndrome	Fryns JP, Jaeken J, Devlieger H, Debucquoy P, Eggermont E, Van den Berghe H.	Acta Paediatr Belg. 1981 Jan-Mar;34(1):47-9.	1981
1	RC15-14	3 y	F	de novo		46,XX,r(15)	7228035	Ring (15) chromosome	Yunis E, Leibovici M, Quintero L.	Hum Genet. 1981;57(2):207-9. doi:	1981
1	RC15-15						7116681	Primary gonadal hypoplasia and dysmorphic features in ring chromosome 15 syndrome	László J, Gaál M, Bösze P.	Clin Genet. 1982 May;21(5):351.	1982
1	RC15-16	33 y	M	nd		46,XY,r(15)[0.92]/46,XY[0.05]/45,XY,-15[0.02]	7105474	Ring chromosome 15: report of a case in an infertile man	Moreau N, Teyssier M.	Clin Genet. 1982 Apr;21(4):272-9. doi: 10.1111/j.1399-0004.1982.tb00763.x.	1982
1	RC15-17						7170952	Ring chromosome 15	Kiss P, Osztovics M.	Acta Paediatr Acad Sci Hung.	1982
1	RC15-18	10.5 y	M	mat		46,XY,r(15)	6220608	A boy with ring chromosome 15 derived from a t(15q;15q) Robertsonian translocation in the mother: cytogenetic and biochemical findings	Neri G, Ricci R, Pelino A, Bova R, Tedeschi B, Serra A.	Am J Med Genet. 1983 Feb;14(2):307-14. doi: 10.1002/ajmg.1320140211.	1983
1	RC15-19	nb	F	de novo		46,XX,r(15)	6468449	Dysplastic features, growth retardation, malrotation of the gut, and fatal ventricular septal defect in a 4-month-old girl with ring chromosome	Otto J, Back E, Fürste HO, Abel M, Böhm N, Pringsheim W.	Eur J Pediatr. 1984 Aug;142(3):229-31. doi: 10.1007/BF00442457.	1984
1	RC15-20	3.5 y	M	de novo		46,XY,r(15)[126]/45,XY,-15[3]/r-var[8]	4040173	Phenotypic delineation of ring chromosome 15 and Russell-Silver syndromes	Wilson GN, Sauder SE, Bush M, Beitins IZ.	J Med Genet. 1985 Jun;22(3):233-6. doi: 10.1136/jmg.22.3.233.	1985
1	RC15-21	7 m	M	de novo		46,XY,r(15)[126]/45,XY,-15[3]/r-var[8]	3962666	Severe growth failure associated with atrophic intestinal mucosa and ring chromosome 15	Kosztolányi G, Pap M.	Acta Paediatr Scand. 1986 Mar;75(2):326-31. doi: 10.1111/j.1651-2227.1986.tb10209.x.	1986
1	RC15-22						3487276	Ring chromosome 15 syndrome. Further delineation of the adult phenotype	Fryns JP, Kleczkowska A, Buttiens M, Jonckheere P, Brouckmans-Buttiens K,	Ann Genet. 1986;29(1):45-8.	1986
0	RC15-23 pt2						3674110	Decreased cell viability of fibroblasts from two patients with a ring chromosome: an in vitro reflection of growth failure?	Kosztolányi G.	Am J Med Genet. 1987 Sep;28(1):181-4. doi: 10.1002/ajmg.1320280125.	1987
1	RC15-24	nb	?	mat	d. 4 m aneurysms	46,XX,r(15)	3596600	Ring chromosome 15 in a mother and her children	Fujimaki W, Baba K, Tatara K, Umezu R, Kusakawa S, Mashima Y.	Hum Genet. 1987 Jul;76(3):302. doi: 10.1007/BF00283630.	1987
0	RC15-24 mat	28 y	F	mother	> 33 y	46,XX,r(15)					
1	RC15-25 pt1	nb	F	de novo		46,XX,r(15)	3063822	Psychological findings in three children with ring 15 chromosome	Borghgraef M, Fryns JP, Van den Berghe H.	J Ment Defic Res. 1988 Aug;32 (Pt 4):337-47. doi: 10.1111/j.1365-2788.1988.tb01422.x.	1988
1	RC15-25 pt2	9.5 y	F			46,XX,r(15)	3063822				
1	RC15-25 pt3	6.5 y	F	de novo		46,XX,r(15)	3063822				
1	RC15-26 pt1	nb	M	de novo		46,XY,r(15)(p11q26)[19]/45,XY,-15[3]	3278612	Two patients with ring chromosome 15 syndrome	Butler MG, Fogo AB, Fuchs DA, Collins FS, Dev VG, Phillips JA 3rd.	Am J Med Genet. 1988 Jan;29(1):149-54. doi: 10.1002/ajmg.1320290119.	1988
1	RC15-26 pt2	3.5 y	F	de novo		46,XX,r(15)(p11q26)[0.80]/45,XX,-15[0.2]	3278612				
1	RC15-27	pn	M	mat nl, pat nd	stillborn, 38 gwks	46,XY,r(150(p11q26)	2746621	Ring chromosome 15 in a patient with features of Fryns' syndrome	de Jong G, Rossouw RA, Retief AE.	J Med Genet. 1989 Jul;26(7):469-70. doi: 10.1136/jmg.26.7.469.	1989
1	RC15-28	12 y	M	de novo		46,XY,r(15)(p12q26.3)	2358298	A case of ring chromosome 15 accompanied by almost normal intelligence	Kitatani M, Takahashi H, Ozaki M, Okino E, Maruoka T.	Hum Genet. 1990 Jun;85(1):138-9. doi: 10.1007/BF00276343.	1990
3	RC15-29						2081000	Ring chromosome 15: follow-up data on physical and psychological development	Fryns JP, Borghgraef M, Kleczkowska A, Van Den Berghe H.	Genet Couns. 1990;1(2):167-72.	1990
1	RC15-30	30 y	F	nd	> 60 y	46,XX,15qs+[0.38>0.60]/46,XX,r(15)[0.48>0.38],45,XX,-15[0.14>0.02]	1746611	Ring chromosome 15 and 15qs+ mosaic: clinical and cytogenetic behaviour spanning 29 years	Smith A, den Dulk G, Viersbach R, Michas J.	Am J Med Genet. 1991 Sep 15;40(4):460-3. doi: 10.1002/ajmg.1320400417.	1991
1	RC15-31, PW66	12 y	F			46,XX,r(16)	1684085	Molecular, cytogenetic, and clinical investigations of Prader-Willi syndrome patients	Robinson WP, Bottani A, Xie YG, Balakrishman J, Binkert F, Mächler M,	Am J Hum Genet. 1991 Dec;49(6):1219-34.	1991
1	RC15-32	2 y		mat		46,XY,r(15)(p12q26.3)	1576754	Familial occurrence of ring chromosome 15	Horigome Y, Kondo I, Kuwajima K, Suzuki T.	Clin Genet. 1992 Apr;41(4):178-80. doi: 10.1111/j.1399-0004.1992.tb03659.x.	1992
0	RC15-32 mat	43 y	F		> 43 y	46,XX,r(15)(p12q26.3)	1576754				
1	RC15-33	11 y	F	de novo		46,XX,r(15)(p12q26.3)	7506614	Ring chromosome 15 involving deletion of the insulin-like growth factor 1 receptor gene in a patient with features of Silver-Russell syndrome	Tamura T, Tohma T, Ohta T, Soejima H, Harada N, Abe K, Niikawa N.	Clin Dysmorphol. 1993 Apr;2(2):106-13.	1993
1	RC15-34	nb	M			46,XY,r(15)(p11.2q26.2	7545237	Good growth response to growth hormone treatment in the ring chromosome 15 syndrome	Nuutinen M, Kouvalainen K, Knip M.	J Med Genet. 1995 Jun;32(6):486-7. doi: 10.1136/jmg.32.6.486.	1995

0	RC15-35 case 1						7789178	Hemizygosity at the insulin-like growth factor I receptor (IGF1R) locus and growth failure in the ring chromosome 15 syndrome	Peoples R, Milatovich A, Francke U.	Cytogenet Cell Genet. 1995;70(3-4):228-34. doi: 10.1159/000134040.	1995
1	RC15-35 case 2	4 y	M	de novo		46,XY,r(15)	7789178				
0	RC15-35 case 3						7789178				
0	RC15-35 case 4						7789178				
1	RC15-35 case 5	3.5 y	F	de novo		46,XX,r(15)	7789178				
1	RC15-36	nb	F	de novo	> 24 y	46,XX,r(15)(p11.2q26.3)	8906535	Ring chromosome 15 syndrome in an adult female	Matsuishi T, Yamada Y, Endo K, Sakai H, Fukushima Y.	J Intellect Disabil Res. 1996 Oct;40 (Pt 5):478-80.	1996
1	RC15-37 case A	14 y	M			46,XY,r(15)	8779316	Distinct 15q genotypes in Russell-Silver and ring 15 syndromes	Rogan PK, Seip JR, Driscoll DJ, Papenhausen PR, Johnson VP, Raskin S, Woodward AL, Butler MG.	Am J Med Genet. 1996 Mar 1;62(1):10-5. doi: 10.1002/(SICI)1096-	1996
1	RC15-37 case B	10 y	M			46,XY,r(15)	8779316				
1	RC15-37 case C	8 y	M			46,XY,r(15)	8779316				
1	RC15-37 case D	3 y	F			46,XX,r(15)	8779316				
1	RC15-37 case E	9 m	M			46,XY,r(15)	8779316				
1	RC15-38	5 y	F	de novo		46,XX,r(15)(p11q26.2)	10594514	In vitro and in vivo responses to short-term recombinant human insulin-like growth factor-1 (IGF-I) in a severely growth-retarded girl with ring chromosome 15 and deletion of a single allele for the type 1 IGF receptor gene	de Lacerda L, Carvalho JA, Stannard B, Werner H, Boguszewski MC, Sandrini R, Malozowski SN, Leroith D, Underwood LE.	Clin Endocrinol (Oxf). 1999 Nov;51(5):541-50. doi: 10.1046/j.1365-2265.1999.00799.x.	1999
1	RC15-39	pn, 20 gwks	M	de novo	top	46,XY,r(15)(p13q26)	11746160	Increased fetal nuchal fold leading to prenatal diagnosis of ring chromosome 15	Liu YH, Chang SD, Chen FP.	Prenat Diagn. 2001 Dec;21(12):1031-3. doi: 10.1002/pd.168.	2001
1	RC15-40	2.5 y	F			46,XX,r(15)	14577679	A girl with cutaneous hyperpigmentation, café au lait spots and ring chromosome 15 without significant deletion	Morava E, Bartsch O, Czako M, Frensel A, Kárteszi J, Kosztolányi GY.	Genet Couns. 2003;14(3):337-42.	2003
1	RC15-41	5 y	M	mat		46,XY,r(15)	12872812	Maternal transmission of a ring chromosome 15	Nikitina NV, Bushueva OA, Nikolaeva EB, Pavlov GV, Lurie IW.	Genet Couns. 2003;14(2):181-6.	2003
1	RC15-42 pt1	pn	M		stillborn, 39 gwks	46,XY,r(15)	15384084	Molecular cytogenetic characterization of ring chromosome 15 in three unrelated patients	Tümer Z, Harboe TL, Blennow E, Kalscheuer VM, Tommerup N,	Am J Med Genet A. 2004 Nov 1;130A(4):340-4. doi:	2004
1	RC15-42 pt2	23 y	M		> 23 y	46,XY,r(15)	15384084				
1	RC15-42 pt3	28 y	M		> 28 y	46,XY,r(15)	15384084				
1	RC15-43 pt4	31 y	F	de novo	> 31 y	46,XX,r(15)(p11q26)	16267671				
1	RC15-44	pn, 18 gwks	F	de novo	top, 22 gwks	46,XX,r(15)	17380471	Molecular characterization of a ring chromosome 15 in a fetus with intra uterine growth retardation and diaphragmatic hernia	Hatem E, Meriam BR, Walid D, Adenen M, Moez G, Ali S.	Prenat Diagn. 2007 May;27(5):471-4. doi: 10.1002/pd.1707.	2007
1	RC15-45	pn, 11 gwks	F	de novo	top, 18 gwks	45,XX,r(15)[21]/45,XX,-15[9]	19455597	Prenatal diagnosis of a fetus with ring chromosome 15 characterized by array-CGH	Manolakos E, Vetro A, Kitmirides S, Papoulidis I, Kosyakova N, Mrasek K, Weise A, Agapitos E, Orru S, Peitsidis P,	Prenat Diagn. 2009 Sep;29(9):884-8. doi: 10.1002/pd.2295.	2009
1	RC15-46 pt1	3 y	F			46,XY,r(15)(p11.2q26)	21995437	Cutis tricolor parvimaculata in two patients with ring chromosome 15 syndrome	Boente Mdel C, Bazan C, Montanari D.	Pediatr Dermatol. 2011 Nov-Dec;28(6):670-3. doi: 10.1111/j.1525-1470.2011.01470.x. Epub 2011 Oct 13.	2011
1	RC15-46 pt2	7 m	F			46,XX,r(15)(p12q26.3)	21995437				
1	RC15-47	8 y	M	de novo		46,XY,r(15)	23042394	[Combined spectral karyotyping and microarray-based comparative genomic hybridization for the diagnosis of a case with ring chromosome 15]	Pan M, Choy KW, Liao C, Lau TK.	Zhonghua Yi Xue Yi Chuan Xue Za Zhi. 2012 Oct;29(5):562-5. doi: 10.3760/cma.j.issn.1003-9406.2012.05.013.	2012
1	RC15-48	7 y	F	de novo	> 27 y	46,XX,r(15)(p13q26.2)	22958471	Twenty-year cytogenetic and molecular follow-up of a patient with ring chromosome 15: a case report	Guilherme RS, Meloni Vde F, Takeno SS, Pellegrino R, Brunoni D, Kulikowski LD, Melaragno MI.	J Med Case Rep. 2012 Sep 7;6:283. doi: 10.1186/1752-1947-6-283.	2012
1	RC15-49	pn, 16 gwks	F	de novo	top, 20 gwks	46,XX,r(15)(p13q26)[16]45,XX,-15[9]	22482980	Prenatal diagnosis of mosaic ring chromosome 15 with abnormal maternal serum Down syndrome screening and Dandy-Walker malformation	Tan SJ, Chen CH, Chen CP, Chen CW, Chen CY, Hwang KS.	Taiwan J Obstet Gynecol. 2012 Mar;51(1):109-11. doi: 10.1016/j.tjog.2012.01.022.	2012
1	RC15-50						24551985	Ring chromosome 15: expanding the phenotype	Eid MM, El-Bassyouni HT, Eid OM, Hamad SA, Elgerzawy A, Zaki MS, El-	Genet Couns. 2013;24(4):417-25.	2013
1	RC15-51	pn, 22 gwks	F	de novo	d. at birth 36 gwks	46,XX,r(15)	25389503	Prenatal diagnosis of a fetus with ring chromosomal 15 by two- and three-dimensional ultrasonography	Britto IS, Regina Silva Herbest S, Tedesco GD, Drummond CL, Bussamra LC, Araujo Júnior E, Ruano R, Ruano SH, Aldrighi JM.	Case Rep Obstet Gynecol. 2014;2014:495702. doi: 10.1155/2014/495702. Epub 2014 Oct 20.	2014
1	RC15-52	1.25 y	F	de novo		46,XX,r(15)[0.96]/45,XX,-15[0.04]	24991444	Ophthalmic treatment and vision care of a patient with rare ring chromosome 15: a case report	Puchalska-Niedbał L, Zajączek S, Petriczko E, Kulik U.	Case Rep Pediatr. 2014;2014:285132. doi: 10.1155/2014/285132. Epub 2014 Jun 3.	2014

1	RC15-53	2 m	M	de novo		46,XY,r(15)(p11.2q26)	29090019	Molecular characterization and evaluation of complex rearrangements in a case of ring chromosome 15	Tewari S, Lubna N, Shah R, Al-Rikabi ABH, Shah K, Sheth J, Sheth F.	Mol Cytogenet. 2017 Oct 25;10:38. doi: 10.1186/s13039-017-0339-z. eCollection 2017.	2017
1	RC15-54 pt3	6 y	F			46,XX,r(15)(p10q26.2)	28899882	Chromosome 15 structural abnormalities: effect on IGF1R gene expression and function	Cannarella R, Mattina T., Condorellw R et al.	Endocr Connect 2017 6(7):528-539	2017
1	RC15-55	child	F	de novo		46,XX,r(15)(p13q26.3)	28604966	[Detection of a patient with ring chromosome 15 by low-coverage massively parallel copy number variation sequencing]	Pan Q, Zhang L, Zhang F, Jin X, Hu Y, Zhu L, Cheng L, Zhang Q, Ning Y.	Zhonghua Yi Xue Yi Chuan Xue Za Zhi. 2017 Jun 10;34(3):406-410. doi: 10.3760/cma.j.issn.1003-9406.2017.03.021.	2017
1	RC15-56	1.25 y	F	de novo		46,XX,r(15)	30442194	Ring chromosome 15 - cytogenetics and mapping arrays: a case report and review of the literature	Paz-Y-Miño C, Guevara-Aguirre J, Paz-Y-Miño A, Velarde F, Armendáriz-Castillo I, Yumiceba V, Hernández JM, García JL, Leone PE.	J Med Case Rep. 2018 Nov 16;12(1):340. doi: 10.1186/s13256-018-1879-5.	2018
1	RC15-57	3 m	F	de novo		46,XX,r(15)	29658137	Cutis tricolor parvimaculata in ring chromosome 15 syndrome: A case report	Ribeiro Dias Barroso C, Silveira Gomes L, Abrantes Silvestre V, Yamada Utagawa C.	Pediatr Dermatol. 2018 May;35(3):e204-e205. doi: 10.1111/pde.13497. Epub 2018 Apr 15.	2018
1	RC15-58	32	M	mat	> 32 y	46,XY,r(15)(p13q26.3)mat[45]/ 45,XY,-15[3]/ 47,XY, + idic r(15)(p13q26.3)x2[1]/ 46,XY,idic r(15)(p13q26.3)[1]	29223476	Fecundity in an infertile man with r(15) - a challenge to the current paradigm	Kalantari H, Karimi H, Almadani SN, Fakhri M, Mokhtari P, Gourabi H, Mohseni Meybodi A.	Reprod Biomed Online. 2018 Feb;36(2):210-218. doi: 10.1016/j.rbmo.2017.10.115. Epub 2017 Nov 22.	2018
0	RC15-58 mat		F	mother	> 67 y	46,XX,r(15)(p13q26.3)[23]/45,XX,- 15[3]/ 48,XXXX,r(15)(p13q26.3)[1]/ 47,XX, + idic r(15)(p13q26.3)x2[1]/ 47,XXX,r(15)(p13q26.3)[1]/ 46,XX,idic	29223476				
1	RC15-59	adult (>20 y)	M		> 20 y	46,XY,r(15)(p12q26.3)	29063501	The normality of sperm in an infertile man with ring chromosome 15: a case report	Nishikawa K, Itoi F, Nagahara M, Jose M, Matsunaga A, Ueda J, Iwamoto T.	J Assist Reprod Genet. 2018 Feb;35(2):251-256. doi: 10.1007/s10815-017-1061-9. Epub 2017 Oct 23.	2018
1	RC15-60	30 y	F		> 30 y	46,XX,r(15)(p12q26.3)[53]/46,XX,r(15;15)(p11.2q26.3;p11.2q11.2)[28]/45,XX, -15[10]/ 46,XX,r(15;15)(p11q26.3;p11q26.3)[4]	33218427	Clinical, cytogenetic and molecular analyses of a rare case with ring chromosome 15 and review of the literature	Shao HY, Wang HL, Wu H, Liu XY, Miao ZY.	Taiwan J Obstet Gynecol. 2020 Nov;59(6):980-984. doi: 10.1016/j.tjog.2020.09.034.	2020
1	RC15-61		F			46, XX, r(15)(p11.2q26.3)[92]/45, XX,-15[9]/46, XX, dic r(15)(p11.2q26.3;p11.2q26.3)[4]	33751532	[Molecular cytogenetic study of a case with ring chromosome 15]	Zhang J, Yang Y, Zhang J, Wang S, Yao F, Zhang Y, Jiang S.	Zhonghua Yi Xue Yi Chuan Xue Za Zhi. 2021 Mar 10;38(3):238-241. doi: 10.3760/cma.j.cn511374-20200214-00080.	2021
1	RC15-62	7 m	F			46,XX,r(15)	34747577	The phenotype and rhGH treatment response of ring Chromosome 15 Syndrome: Case report and literature review	Chen M, Ke X, Liang H	Mol Genet Genomic Med. 2021 Dec;9(12):e1842	2021
**75**	**62**										
1	RC16-1	33 y	F		> 33 y	PB: 46,XX,r(16)[26]/45,XX,-16[4]/47,XX,+7,r(16)[3]; SF: 46,XX,r(16)[39]/45,XX,-16[1]/47,XX,r(16)+18[1]	7327577	Ring chromosome 16	Neidengard L, Sparkes RS.	Hum Genet. 1981;59(2):175-7. doi: 10.1007/BF00293072.	1981
1	RC16-2	3.5 y	F	de novo		46,XX,r(16)[83]/45,XX,-16[17]	2464927	Developmental delay, short stature, and minor facial anomalies in a child with ring chromosome	Chodirker BN, Ray M, McAlpine PJ, Riordan D, Vust A, Pugh D, Chudley AE.	Am J Med Genet. 1988 Sep;31(1):145-51. doi:	1988
1	RC16-3	4.5 y	F			46,XX,r(16)	2195979	Ring chromosome 16: a new case	Vianello MG, Cottafava F, Bartoli D, Franzone G, Casazzava R, Gastaldi R.	Ann Genet. 1990;33(1):36-9.	1990
1	RC16-4	5 y	M	de novo		46,XY,r(16)(p13q24)[0.56]/46,XY[0.44]	9137886	Delineation of a ring chromosome 16 by the FISH-technique: a case report with review	Conte RA, Kleyman SM, Kharode C, Verma RS.	Clin Genet. 1997 Mar;51(3):196-9. doi: 10.1111/j.1399-0004.1997.tb02452.x.	1997
1	RC16-5	4 m	F	de novo		46,XX,r(16)(p13q24)	11807861	Molecular characterization of a ring chromosome 16 from a patient with bilateral cataracts	He W, Tuck-Muller CM, Martínez JE, Li S, Rowley ER, Wertelecki W.	Am J Med Genet. 2002 Jan 1;107(1):12-7. doi: 10.1002/ajmg.10091.	2002
**5**	**5**										
1	RC17-1	pn, 16 gwks	F	de novo	top	46,XX,r(17)/45,XX,-17	1150287	Ring 17 chromosome detected by amniocentesis	Weinberg AG, Bair JL, Harrod MJ.	Humangenetik. 1975 Jul 23;28(3):269-72. doi: 10.1007/BF00278557.	1975
1	RC17-2	6 y	M			46,XY,r(17)	121681	Ring chromosome 17 in a mentally retarded boy	Qazi OH, Madahar C, Kanchanapoomi R, Giridharan R, Beller E.	Ann Genet. 1979;22(4):234-8.	1979

1	RC17-3	5 m	M	de novo		46,XY,r(17)	7241549	An infant with ring 17 chromosome and unusual dermatoglyphs: a new syndrome?	Carpenter NJ, Leichtman LG, Stamper S, Say B.	J Med Genet. 1981 Jun;18(3):234-6. doi: 10.1136/jmg.18.3.234.	1981
1	RC17-4	4 y	M	de novo	> 26 y	46,XY,r(17)(p13q25)	7048927	Brief clinical report: ring chromosome 17 in a mentally retarded young man - clinical, cytogenetic, and biochemical investigations	Chudley AE, Pabello PD, McAlpine PJ, Nickel BE, Shokeir MH.	Am J Med Genet. 1982 Jun;12(2):219-25. doi: 10.1002/ajmg.1320120212.	1982
1	RC17-5	1 m	M	de novo		46,XY,r(17)(p13.3q25.3)[92/100]	6834189	Miller-Dieker syndrome: lissencephaly and monosomy 17p	Dobyns WB, Stratton RF, Parke JT, Greenberg F, Nussbaum RL, Ledbetter	J Pediatr. 1983 Apr;102(4):552-8. doi: 10.1016/s0022-3476(83)80183-	1983
0	RC17-6						6745939	New chromosomal syndrome: Miller-Dieker syndrome and monosomy 17p13	Stratton RF, Dobyns WB, Airhart SD, Ledbetter DH.	Hum Genet. 1984;67(2):193-200. doi: 10.1007/BF00273000.	1984
0	RC17-7 MDS-1						3189330	Molecular detection of microscopic and submicroscopic deletions associated with Miller-Dieker syndrome	vanTuinen P, Dobyns WB, Rich DC, Summers KM, Robinson TJ, Nakamura Y, Ledbetter DH.	Am J Hum Genet. 1988 Nov;43(5):587-96.	1988
1	RC17-8						2694873	[Ring chromosome 17 and recurring pneumopathy]	Lambruschini Ferri N, Ortola Castells ME, Rosell Andreo J, Ballesta Martínez	An Esp Pediatr. 1989 Nov;31(5):478-80.	1989
1	RC17-9	1.5 m	F	de novo	d. 9 m	46,XX,r(17)	1711306	Miller-Dieker syndrome with ring chromosome 17	Sharief N, Craze J, Summers D, Butler L, Wood CB.	Arch Dis Child. 1991 Jun;66(6):710-2. doi: 10.1136/adc.66.6.710.	1991
1	RC17-10						1995042	Flecked retina associated with ring 17 chromosome	Charles SJ, Moore AT, Davison BC, Dyson HM, Willatt L.	Br J Ophthalmol. 1991 Feb;75(2):125-7. doi: 10.1136/bjo.75.2.125.	1991
1	RC17-11	9 y	F			46,XX,r(17)	1381884	Ring chromosome 17. Case report and review of the literature	Teyssier M, Charrin C, Corgiolu Theuil G, David L.	Ann Genet. 1992;35(2):75-8.	1992
1	RC17-12						8002828	Flecked retina associated with café au lait spots, microcephaly, epilepsy, short stature, and ring 17 chromosome	Gass JD, Taney BS.	Arch Ophthalmol. 1994 Jun;112(6):738-9. doi: 10.1001/archopht.1994.01090180036013.	1994
1	RC17-13	3 y	F				8986275	Mosaic partial trisomy 17 due to a ring chromosome identified by fluorescence in situ	Morrison PJ, Smith NM, Martin KE, Young ID.	Am J Med Genet. 1997 Jan 10;68(1):50-3.	1997
1	RC17-14	4 m	M	de novo		46,XY,r(17)(p13.3q25.3)[43/50]/45,XY,-17[7/50]	10519353	Ring chromosome 17 syndrome with monosomy 17 mosaicism: case report and literature review	Endo A, Uesato T, Minato M, Takada M, Takahashi S, Harada K.	Acta Paediatr. 1999 Sep;88(9):1040-3. doi: 10.1080/08035259950168595.	1999
1	RC17-15 case 1	2 y	M		> 28 y	46,XY,r(17)(p13q25)	12974742	Ring chromosome 17: phenotype variation by deletion size	Shashi V, White JR, Pettenati MJ, Root SK, Bell WL.	Clin Genet. 2003 Oct;64(4):361-5. doi: 10.1034/j.1399-0004.2003.00146.x.	2003
1	RC17-15 case 1	14 y	M			46,XY,r(17)(p13q25)	12974742				
1	RC17-16	4 y	M			46,XY,r(17)	17884758	Ring chromosome 17 epilepsy may resemble that of ring chromosome 20 syndrome	Ricard-Mousnier B, N'Guyen S, Dubas F, Pouplard F, Guichet A.	Epileptic Disord. 2007 Sep;9(3):327-31. doi: 10.1684/epd.2007.0121. Epub 2007 Sep 20.	2007
1	RC17-17	4 m	F	de novo		46,XX,r(17)(p13q25)[83]/45,XX,-17[12]/r-var[5]	17163520	A girl with neurofibromatosis type 1, atypical autism and mosaic ring chromosome 17	Havlovicova M, Novotna D, Kocarek E, Novotna K, Bendova S, Petrak B,	Am J Med Genet A. 2007 Jan 1;143A(1):76-81. doi:	2007
1	RC17-18						17996402	Molecular cloning and analysis of breakpoints on ring chromosome 17 in a patient with autism	Vazna A, Havlovicova M, Sedlacek Z.	Gene. 2008 Jan 15;407(1-2):186-92. doi: 10.1016/j.gene.2007.10.009.	2008
1	RC17-19	16 y	M	de novo	> 23 y	46,XY,r(17)(p13.3q25)	19197320	Flecked retina associated with ring 17 chromosome	Kumari R, Black G, Dore J, Lloyd IC.	Eye (Lond). 2009 Nov;23(11):2134-5. doi: 10.1038/eye.2008.392. Epub	2009
1	RC17-20	4 y	M			46,XY,r(17)(p13q25)[27]/45,XY,-17[3]	19793054	Mild ring 17 syndrome shares common phenotypic features irrespective of the chromosomal breakpoints location	Surace C, Piazzolla S, Sirleto P, Digilio MC, Roberti MC, Lombardo A, D'Elia G, Tomaiuolo AC, Petrocchi S, Capolino R, El Hachem M, Claps	Clin Genet. 2009 Sep;76(3):256-62. doi: 10.1111/j.1399-0004.2009.01203.x. PMID: 19793054.	2009
0	RC17-21						24393457	Telomere shortening and telomere position effect in mild ring 17 syndrome	Surace C, Berardinelli F, Masotti A, Roberti MC, Da Sacco L, D'Elia G, Sirleto P, Digilio MC, Cusmai R, Grotta S, Petrocchi S, Hachem ME, Pisaneschi	Epigenetics Chromatin. 2014 Jan 7;7(1):1. doi: 10.1186/1756-8935-7-1.	2014
1	RC17-22	9 y	F			46,XX,r(17)	25635406	Ring 17 syndrome: first clinical report without intellectual disability	de Palma L, De Carlo D, Lenzini E, Boniver C, Tarantino V, Bacci B, Vecchi	Epileptic Disord. 2015 Mar;17(1):84-7; quiz 88. doi:	2015
1	RC17-23	4 y	F		> 31 y	46,XX,r(17)	29456482	Ring Chromosome 17 Not Involving the Miller-Dieker Region: A Case with Drug-Resistant Epilepsy	Coppola A, Morrogh D, Farrell F, Balestrini S, Hernandez-Hernandez L, Krithika S, Sander JW, Waters JJ,	Mol Syndromol. 2017 Dec;9(1):38-44. doi: 10.1159/000479949. Epub 2017 Sep 15.	2017
**21**	**23**										
0	RC18-1						14242103	[17-18 RING-CHROMOSOMES AND CONGENITAL MALFORMATIONS IN A YOUNG GIRL]	DE GROUCHY J, LEVEQUE B, DEBAUCHEZ C, SALMON C, LAMY M,	Ann Genet. 1964;7:17-23.	1964
1	RC18-2	10 y	M	na		BL: 46,XY,r(18)[121]/45,XY,-18[13] SF: 46,XY,r(18)[18]	5619991	Ring chromosome 18 in a patient with multiple anomalies	Palmer CG, Fareed N, Merritt AD.	J Med Genet. 1967 Jun;4(2):117-23. doi: 10.1136/jmg.4.2.117.	1967
1	RC18-3						5301753	[An observation of ring chromosome 18 (18r)]	De Grouchy J, Herrault A, Cohen-Solal	Ann Genet. 1968 Mar;11(1):33-8.	1968
1	RC18-4						5747161	[Ring chromosome 18]	Hooft C, Haentjens P, Orye E,	Acta Paediatr Belg. 1968;22(2):69-	1968

1	RC18-5	14 y	F			46,XX,r(18)	4171782	IgA absence associated with a ring-18 chromosome	Finley SC, Finley WH, Noto TA, Uchida IA, Roddam RF.	Lancet. 1968 May 18;1(7551):1095-6. doi: 10.1016/s0140-6736(68)91453-0.	1968
1	RC18-6	11 y	M			46,XY,r(18)	4173009	IgA and ring-18 chromosome	Richards BW, Hobbs JR.	Lancet. 1968 Jun 29;1(7557):1426-7. doi: 10.1016/s0140-6736(68)92001-1.	1968
0	RC18-7						4173760	IgA and ring-18 chromosome	Warren RJ.	Lancet. 1968 Aug 10;2(7563):350. doi: 10.1016/s0140-6736(68)90557-6.	1968
1	RC18-8	12 y	F	mat		46,XX,r(18)	4183928	Ring chromosome 18 and gamma-M-globulin abnormality	Jensen K, Christensen KR, Jacobsen P, Nielsen J, Friedrich U, Tsuboi T.	Lancet. 1969 Aug 30;2(7618):497-8. doi: 10.1016/s0140-6736(69)90206-2.	1969
0	RC18-8 mat	48 y			> 48 y	46,XX,r(18)	4183928				
1	RC18-9						5308385	[A new observation of a ring chromosome 18]	Deminatti M, Dupuis C, Maillard E, Delmas-Marsalet Y, Bulteel MF.	Ann Genet. 1969 Jun;12(2):126-9.	1969
1	RC18-10	10 m	F			46,XX,r(18)	5365577	Ring chromosome 18 (46, XX, 18r)	Cenani A, Pfeiffer RA, Simon HA.	Humangenetik. 1969;7(4):351-2. doi: 10.1007/BF00283558.	1969
1	RC18-11						5495679	[Ring chromosome 18]	Ricci N, Dallapiccola B, Ventimiglia B, Preto G.	Acta Genet Med Gemellol (Roma). 1970 Jul;19(3):439-47.	1970
1	RC18-12	10 y	M	de novo		46,XY,r(18)	5512218	Ring chromosome 18 in a mentally retarded boy	Richards BW, Rundle AT, Zaremba J, Stewart A.	J Ment Defic Res. 1970 Jun;14(2):174-86. doi: 10.1111/j.1365-2788.1970.tb01112.x.	1970
1	RC18-13						5534732	Ring chromosome 18 in mother and daughter	Christensen KR, Friedrich U, Jacobsen P, Jensen K, Nielsen J, Tsuboi T.	J Ment Defic Res. 1970 Mar;14(1):49-67. doi:	1970
1	RC18-14	6 y	F			46,XX,r(18)	5478808	[Ring-chromosome 18 and IgA deficiency in a 6-year-old girl (46,XX,18r)]	Murken JD, Salzer G, Kunze D.	Z Kinderheilkd. 1970;109(1):1-10.	1970
2	RC18-15						5313142	[2 cases of ring 18 chromosome]	Deminatti M, Debeugny P, Croquette-Bulteel MF, Delmas-Marsalet Y.	Ann Genet. 1970 Sep;13(3):149-55.	1970
1	RC18-16	2 y	F			46,XX,r(18)	5091271	Intestinal nodular lymphoid hyperplasia, hypogammaglobulinemia, and hematologic abnormalities in a child with a ring 18 chromosome	Michaels DL, Go S, Humbert JR, Dubois RS, Stewart JM, Ellis EF.	J Pediatr. 1971 Jul;79(1):80-8. doi: 10.1016/s0022-3476(71)80062-8.	1971
0	RC18-17						4645993	[Ring chromosome 18. Clinical study and caryologic considerations]	Giovannucci Uzielli ML, Carbone C, Calabri G.	Minerva Pediatr. 1972 Oct 20;24(36):1707-14.	1972
1	RC18-18	nb	F	de novo	d. 7 wks	46,XX,r(18)	5018394	Primary hypoparathyroidism associated with ring chromosome 18	Olambiwonnu NO, Ebbin AJ, Frasier SD.	J Pediatr. 1972 May;80(5):833-5. doi: 10.1016/s0022-3476(72)80140-9.	1972
1	RC18-19	10.75 y	F	de novo		46,XX,+r(18)[87]/45,XX,-18[12]	5025476	Conenital hypothyroidism in association with a ring chromosome 18	Winter JS, Ahluwalia K, Ray M.	J Med Genet. 1972 Mar;9(1):122-6. doi: 10.1136/jmg.9.1.122.	1972
1	RC18-20	3 m	F			46,XX,r(18)	4565746	[Ring chromosome 18. 18p-/18q- -deletion-syndrome]	Kunze J, Stephan E, Tolksdorf M.	Humangenetik. 1972;15(4):289-318. doi: 10.1007/BF00281730.	1972
1	RC18-21						1081367	A case of ring 18 chromosome in a sibship with multiple spontaneous abortions	Coco R, Barreiro CZ, Penchaszadeh VB.	Ann Genet. 1975 Jun;18(2):135-7.	1975
1	RC18-22						1246241	[Ring chromosome 18]	Kunze J, Spranger J, Tolksdorf M.	Monatsschr Kinderheilkd. 1976	1976
1	RC18-23						555611	[A child with a ring 18 chromosome : 46,XX,r(18) and a decreased enzymatic activity of erythrocyte peptidase A (author's transl)]	Serville F, Guillard JM, Junien C, Gauville J.	Ann Pediatr (Paris). 1979 Dec;26(10):711-5.	1979
1	RC18-24						7418077	[A mosaic of ring chromosome 18]	Srsen S, Volna J, Miklerová M.	Cesk Pediatr. 1980 Aug;35(8):419-22.	1980
1	RC18-25						6444875	Selective IgA deficiency: clinical and immunological evaluation of 50 pediatric patients	Burgio GR, Duse M, Monafo V, Ascione A, Nespoli L.	Eur J Pediatr. 1980 Mar;133(2):101-6. doi: 10.1007/BF00441577.	1980
1	RC18-26 case 1	16 y	M			46,XY,r(18)	6549034	Predisposition to autoimmune thyroiditis in ring chromosome 18 syndrome	Fukushima Y, Fukuda T, Kuroki Y, Niikawa N, Matsuura N, Yamada Y,	Jinrui Idengaku Zasshi. 1984 Jun;29(2):127-32. doi:	1984
1	RC18-26 case 2	1 y	F			46,XX,r(18)	6549034				
1	RC18-26 case 3	12 y	M			46,XY,r(18)/46,XY	6549034				
1	RC18-26 case 4	1 y	F			46,XX,r(18)	6549034				
1	RC18-26 case 5	6 y	M			46,XY,r(18)/46,XY	6549034				
1	RC18-27						4034214	[Mechanism of a genetically conditioned failure to thrive shown in a patient with ring chromosome 18]	Heinz-Erian P, Sigmund J, Frisch H, Kazda S, Bindra A, Rhomberg K.	Padiatr Padol. 1985;20(3):231-41.	1985
1	RC18-28						3760834	[Transmission of an 18 ring chromosome in two generations in subjects of normal phenotype]	Faugeras C, Barthe D.	J Genet Hum. 1986 Aug;34(3-4):313-20.	1986

1	RC18-29	6 m	M	mat	> 23 y (mat)	46,XY,r(18)mat	3706404	Ring chromosome 18 in a mother and son	Donlan MA, Dolan CR.	Am J Med Genet. 1986 May;24(1):171-4. doi: 10.1002/ajmg.1320240121.	1986
1	RC18-30						3601789	[A case of a girl with a ring chromosome 18 in the karyotype]	Midro AT, Czerwińska-Ciechan K, Wiśniewski L, Sawicka A,	Pol Tyg Lek. 1987 Mar 2;42(9):260-3.	1987
1	RC18-31	4 y	M			46,XY,r(18)	3565953	[Ring chromosome 18 46,XY,r(18)]	Benito E, Serrano A, Moreno F, Espinosa J, Dávila MJ, Cardesa JJ, Galán	An Esp Pediatr. 1987 Feb;26(2):121-3.	1987
1	RC18-[1]	4 y	F			46,XX,r(18)/46,XX,add(18p)	3652494	Chromosome studies in IgA-deficient patients	Taalman RD, et al.	Clin Genet. 1987: 32(2):81-7	1987
1	RC18-32	7 m	F			46,XX,r(18)	3367915	[Van der Woude syndrome in combination with ring chromosome 18]	Kalker U, Gabriel M, Jacobi G.	Monatsschr Kinderheilkd. 1988 Feb;136(2):95-8.	1988
1	RC18-33	6 m	F	de novo		46,XX,r(18)(p11.3q23)	3242534	Ring 18 chromosome with mental retardation, hemidysmorphism, and mitochondrial encephalomyopathy	Amit R, Gutman A, Udassin R, Barash V, Kohn G.	Pediatr Neurol. 1988 Sep-Oct;4(5):301-4. doi: 10.1016/0887-8994(88)90070-7.	1988
1	RC18-34	3.5 y	M	de novo		46,XY,r(18)	3178273	Growth hormone, suspected gonadotrophin deficiency, and ring 18 chromosome	Abusrewil SS, McDermott A, Savage DC.	Arch Dis Child. 1988 Sep;63(9):1090-1. doi: 10.1136/adc.63.9.1090.	1988
1	RC18-35						1462429	[Ring chromosome 18]	Andersen UM.	Ugeskr Laeger. 1992 Nov 9;154(46):3250-1.	1992
1	RC18-36	pn, 15 gwks	F	de novo	top	46,XX,r(18)(p11q12)/45,XX,-18	1494547	Prenatal diagnosis of monosomy 18 and ring chromosome 18 mosaicism	Eiben B, Unger M, Stoltenberg G, Rutt G, Goebel R, Meyer A, Gamerdinger U,	Prenat Diagn. 1992 Nov;12(11):945-50. doi: 10.1002/pd.1970121114.	1992
1	RC18-37 pt1	2 y	M			46,XY,r(18)[0.50]/46,XY[0.50]	1516230	Autism and ring chromosome 18 mosaicism	Fryns JP, Kleczkowska A.	Clin Genet. 1992 Jul;42(1):55. doi: 10.1111/j.1399-0004.1992.tb03139.x.	1992
1	RC18-37 pt2	37 y	F		> 37 y	46,XX[0.75]/46,XX,r(18)[0.25]	1516230				
1	RC18-38	nb	M	mat	> 20 y (mat)	46,XY,r(18)mat	1524410	Transmission of ring chromosome 18 46,XX/46,XX,r(18) mosaicism in a mother and ring chromosome 18 syndrome in her son	Fryns JP, Kleczkowska A, Smeets E, Van Den Berghe H.	Ann Genet. 1992;35(2):121-3.	1992
1	RC18-39	nb	F	mat		46,XX,r(18)	8301656	Transmission of a ring chromosome 18 from a mother with 46,XX/47,XX, + r(18) mosaicism to her daughter, resulting in a 46,XX,r(18) karyotype	Jenderny J, Caliebe A, Beyer C, Grote W.	J Med Genet. 1993 Nov;30(11):964-5. doi: 10.1136/jmg.30.11.964.	1993
0	RC18-39 mat	28 y	F		> 28 y	46,XX[98]/47,XX,r(18)[2]	8301656				
1	RC18-40 pt 5	17 y	M			46,XY,r(18)	7839807	Epileptic seizures, arthrogryposis, and migrational brain disorders: a syndrome?	Brodtkorb E, Torbergsen T, Nakken KO, Andersen K, Gimse R, Sjaastad O.	Acta Neurol Scand. 1994 Oct;90(4):232-40. doi: 10.1111/j.1600-0404.1994.tb02713.x.	1994
1	RC18-41	10 m	M			46,XY,r(18)	8157016	Hypothalamic growth hormone deficiency in a patient with ring chromosome 18	Meloni A, Boccone L, Angius L, Loche S, Falchi AM, Cao A.	Eur J Pediatr. 1994 Feb;153(2):110-2. doi: 10.1007/BF01959220.	1994
1	RC18-42	1 m	M	mat nl, pat nd		46,XY,r(18)	8157419	Nasal pyriform aperture stenosis and the holoprosencephaly spectrum	Tavin E, Stecker E, Marion R.	Int J Pediatr Otorhinolaryngol. 1994 Jan;28(2-3):199-204. doi:	1994
1	RC18-43	pn, 16 gwks	F	de novo	top	46,XX,r(18)/45,XX,-18/47,XX,r(18),+mar	8958334	Ring chromosome 18 in a fetus with only facial anomalies	Los FJ, van den Berg C, Braat PG, Cha'ban FK, Kros JM, Van Opstal D.	Am J Med Genet. 1996 Dec 11;66(2):216-20. doi: 10.1002/(SICI)1096-	1996
1	RC18-44	5 y	F	de novo		46,XX,r(18)(p11q23)	8942020	Growth hormone neurosecretory dysfunction associated with ring chromosome 18	Aritaki S, Takagi A, Someya H, Jun L.	Acta Paediatr Jpn. 1996 Oct;38(5):544-8. doi:	1996
1	RC18-45	3 y	M	de novo		46,XY,r(18)(p11.2q21.33)	9453033	Abnormal myelination in a patient with ring chromosome 18	Nakayama J, Hamano K, Shimakura Y, Iwasaki N, Nakahara C, Imoto N, Kobayashi K, Arinami T, Hamaguchi H.	Neuropediatrics. 1997 Dec;28(6):335-7. doi: 10.1055/s-2007-973727.	1997
1	RC18-46	nb	F	de novo	d. shortly	46,XX,r(18)(p11q12)	9354845	Anencephaly with holoprosencephalic facies due to ring chromosome 18	Bird LM, Pretorius DH, Mendoza AE, Jones MC.	Clin Dysmorphol. 1997 Oct;6(4):351-8. doi: 10.1097/00019605-199710000-00009.	1997
1	RC18-47	pn	F	de novo	top	46,XX,r(18)[27]/45,XX,-18[13]	9813427	Prenatal diagnosis and fetopathological findings in a fetus with ring chromosome 18	Thies U, Bartels I, von Beust G, Bink K, Hansmann I, Rehder H, Suren A, Zoll B.	Fetal Diagn Ther. 1998 Sep-Oct;13(5):315-20. doi:	1998
1	RC18-48	nb	F	de novo		46,XX,r(18)(p11.2q23)[89]/45,XX,-18[5]/46,XX,dic r(18)[6]	9568951	Agammaglobulinaemia in a girl with a mosaic of ring 18 chromosome	Litzman J, Brysová V, Gaillyová R, Thon V, Pijácková A, Michalová K, Zemanová	J Paediatr Child Health. 1998 Feb;34(1):92-4. doi: 10.1046/j.1440-	1998
1	RC18-49	4.25 y	M	de novo		46,XY,r(18)	10051018	Insulin dependent diabetes mellitus (IDDM) and autoimmune thyroiditis in a boy with a ring chromosome 18: additional evidence of autoimmunity or IDDM gene(s) on chromosome 18	Dacou-Voutetakis C, Sertedaki A, Maniatis-Christidis M, Sarri C, Karadima G, Petersen MB, Xaidara A, Kanariou M, Nicolaidou P.	J Med Genet. 1999 Feb;36(2):156-8.	1999
1	RC18-50	adult	F		> 25 y	46,XX,r(18)(p11.3q23)[32]/45,XX,-18[4]	11754054	First familial case of ring chromosome 18 and monosomy 18 mosaicism	Yardin C, Esclaire F, Terro F, Baclet MC, Barthe D, Laroche C.	Am J Med Genet. 2001 Dec 1;104(3):257-9.	2001
1	RC18-51 case 1	5 m	F			46,XX,r(18)	11424138	Clinical and molecular-cytogenetic studies in seven patients with ring chromosome 18	Stankiewicz P, Brozek I, Hélias-Rodzewicz Z, Wierzba J, Pilch J, Bocian E, Balcerska A, Wozniak A, Kardaś I,	Am J Med Genet. 2001 Jul 1;101(3):226-39. doi: 10.1002/1096-8628(20010701)101:3<226::aid-	2001
1	RC18-51 case 2	3 m	F			46,XX,r(18)(p11.32q21.3)	11424138				

1	RC18-51 case 3	10 y	M			46,XY,r(18)(p11.32q21.3)	11424138				
1	RC18-51 case 4	2 y	M			46,XY,r(18)(p11.1q22.3)	11424138				
1	RC18-51 case 5	3 m	F			46,XX,r(18)(p11.1q22.3)	11424138				
0	RC18-51 case 6	nb	F			46,XX,add(18)(p11.2)[11]/47,XX,add(18),+r[5]	11424138				
0	RC18-51 case 7	10 y	F			46,XX,add(18)(p11.32)[60]/47,XX,add(18),+r[	11424138				
1	RC18-52	pn, 20 gwks			top, 23 gwks	AF: 46,XY,r(18)(p11q23)[103]/45,XY,-18[13] UCB: 46,XX,r(18)[57]/45,XY,-18[12] CVS: 46,XY	11438954	Complete karyotype discrepancy between placental and fetal cells in a case of ring chromosome 18	Fischer W, Dermitzel A, Osmers R, Pruggmayer M.	Prenat Diagn. 2001 Jun;21(6):481-3. doi: 10.1002/pd.99.	2001
1	RC18-53	nb	F			46,XX,r(18)(p11.3q23)[88]/47,XX,r(18)(p11.3 q23),+r(18)(p11.22q12.2)[112]	12400074	Meiotic origin of two ring chromosomes 18 in a girl with developmental delay	Baumer A, Giovannucci Uzielli ML, Guarducci S, Lapi E, Röthlisberger B,	Am J Med Genet. 2002 Nov 15;113(1):101-4. doi:	2002
1	RC18-54	nb	F	de novo		47,XX,r(18),+mar[80]/46,XX,mar[10]/46,XX,r(	12574939	Chromosome 18 replaced by two ring chromosomes of chromosome 18 origin	Miller K, Pabst B, Ritter H, Nürnberg P, Siebert R, Schmidtke J, Arslan-Kirchner	Hum Genet. 2003 Apr;112(4):343-7. doi: 10.1007/s00439-002-0885-1.	2003
1	RC18-55	1 y	F			46,XX,r(16)	17160222	[Growth hormone deficiency, hypothyroidism and ring chromosome 18: case report]	Thomas JV, Mezzasalma DF, Teixeira AM, Campos LN, Luescher JL, Beserra	Arq Bras Endocrinol Metabol. 2006 Oct;50(5):951-6. doi:	2006
1	RC18-56		F			46,XX,r(18)	16850691	Thai girl with ring chromosome 18 (46XX, r18)	Sripanidkulchai R, Suphakunpinyo C, Jetsrisuparb C, Luengwattanawanich S.	J Med Assoc Thai. 2006 Jun;89(6):878-81.	2006
1	RC18-57 case A	pn, 14 gwks	M		top, 15 gwks	46,XY,r(18)[36]/45,XY,-18[7]	17595336	Three unusual but cytogenetically similar cases with up to five different cell lines involving structural and numerical abnormalities of chromosome 18	Carreira IM, Mascarenhas A, Matoso E, Couceiro AB, Ramos L, Dufke A, Mazauric M, Stressig R, Kosyakova N, Melo JB, Liehr T.	J Histochem Cytochem. 2007 Nov;55(11):1123-8. doi: 10.1369/jhc.7A7244.2007. Epub 2007 Jun 26.	2007
1	RC18-57 case B	pn	M		top, 34 gwks	46,XY,t(18)[0.90]/45,XY,-18[0.10]	17595336				
1	RC18-57 MZ twin C1/C2	6 m	F			46,XX,r(18)(p11.32q22.3)	17595336				
1	RC18-58	1.6 y	M			46,XY,r(18)	18935916	Clinical course of a 20-month-old child diagnosed prenatally with mosaic ring chromosome 18 and monosomy 18	Mello AL, Crotwell PL, Flanagan JD, Woltanski AR, Keppen LD, Van Eerden P, Boyle JG, Stein Q.	S D Med. 2008 Sep;61(9):327-9, 331.	2008
1	RC18-59	6.5 y	F	de novo		46,XX,r(18)(p11.3q23)[0.86]/45,XX,-18[0.07]/46,XX,dic r(18)[0.07]	17668239	An unexpected finding in a child with neurological problems: mosaic ring chromosome 18	Koç A, Kan D, Karaer K, Ergün MA, Karaoğuz MY, Gücüyener K, Hinreiner	Eur J Pediatr. 2008 Jun;167(6):655-9. doi: 10.1007/s00431-007-0568-y.	2008
1	RC18-60	pn, 16 gwks	M		cp	AF: 46,XY,r(18)[27]/45,XY,-18[5]/46,XY[5]	21056319	Mosaic ring chromosome 18, ring chromosome 18 duplication/deletion and disomy 18: perinatal findings and molecular cytogenetic characterization by fluorescence in situ hybridization and array comparative genomic	Chen CP, Kuo YT, Lin SP, Su YN, Chen YJ, Hsueh RY, Lin YH, Wu PC, Lee CC, Chen YT, Wang W.	Taiwan J Obstet Gynecol. 2010 Sep;49(3):327-32. doi: 10.1016/S1028-4559(10)60069-1.	2010
1	RC18-61	9 m	F		> 25 y	46,XX,r(18)(p11.2q23)[98]/46.XX,dic r(18)[2]	19860520	A case of ring chromosome 18 syndrome treated with a combined orthodontic-prosthodontic	Ono T, Okuma M, Hamada T, Motohashi N, Moriyama K.	Cleft Palate Craniofac J. 2010 Mar;47(2):201-10. doi: 10.1597/08-	2010
1	RC18-62	22 y	F		> 22 y	46,XX,r(18)(p11.3q23)[14]/46,XX[36]	22090726	Prenatal diagnosis in a mentally retarded woman with mosaic ring chromosome 18	Bagherizadeh E, Behjati F, Saberi SH, Shafeghati Y.	Indian J Hum Genet. 2011 May;17(2):111-3. doi: 10.4103/0971-6866.86201.	2011
1	RC18-63	9 y	F	de novo		46,XX,r(18)(p11.3q23)[92]/45,XX,-18[7]/46,XX[1]	21333764	De novo mosaic ring chromosome 18 in a child with mental retardation, epilepsy and immunological problems	Lo-Castro A, El-Malhany N, Galasso C, Verrotti A, Nardone AM, Postorivo D, Palmieri C, Curatolo P.	Eur J Med Genet. 2011 May-Jun;54(3):329-32. doi: 10.1016/j.ejmg.2011.02.004. Epub 2011 Feb 17.	2011
1	RC18-64	3.5 y	F		> 20 y	46,XX,r(18)(p11.3q23)	22145491	Autoimmune polyendocrinopathy associated with ring chromosome 18	Jain N, Reitnauer PJ, Rao KW, Aylsworth AS, Calikoglu AS.	J Pediatr Endocrinol Metab. 2011;24(9-10):847-50. doi: 10.1515/jpem.2011.320.	2011
1	RC18-65	9 y	M			46,XY,r(18)[0.35]/46,XY[0.65]	21848009	Ring chromosome 18 in a child with febrile	Celep F, Sonmez FM, Kul S, Ucar F,	Genet Couns. 2011;22(2):165-71.	2011
0	RC18-66						21484996	Familial ring (18) mosaicism in a 23-year-old young adult with 46,XY,r(18) (::p11→q21::)/46,XY karyotype, intellectual disability, motor retardation and single maxillary incisor and in his phenotypically normal mother, karyotype	Balci S, Tümer C, Karaca C, Bartsch O	Am J Med Genet A. 2011 May;155A(5):1129-35. doi: 10.1002/ajmg.a.33868. Epub 2011 Apr 11. PMID: 21484996.	2011
1	RC18-67	4 m	M			46,XY,r(18)(p11.2q23)	22947348	Hypothyroidism and levothyroxine-responsive liver dysfunction in a patient with ring chromosome 18 syndrome	Ohkubo K, Ihara K, Ohga S, Ishimura M, Hara T.	Thyroid. 2012 Oct;22(10):1080-3. doi: 10.1089/thy.2011.0521. Epub 2012 Sep 4.	2012
1	RC18-68	nb	F			46,XX,r(18)[5]/46,XX[105] CMA[0.26]	22887345	Tissue-limited ring chromosome 18 mosaicism as a cause of Pitt-Hopkins syndrome	Takenouchi T, Yagihashi T, Tsuchiya H, Torii C, Hayashi K, Kosaki R, Saitoh S, Takahashi T, Kosaki K.	Am J Med Genet A. 2012 Oct;158A(10):2621-3. doi: 10.1002/ajmg.a.35230. Epub 2012 Aug 10.	2012
1	RC18-69	2 m	F			46,XX,r(18)	22290857	Abnormal myelination in ring chromosome 18 syndrome	Benini R, Saint-Martin C, Shevell MI, Bernard G.	J Child Neurol. 2012 Aug;27(8):1042-7. doi: 10.1177/0883073811430268. Epub 2012 Jan 30.	2012
1	RC18-70	6 y	M		d. 6 y	46,XY,r(18)(p11.2q21.33)	21669507	Abnormal brain MRI signal in 18q-syndrome not due to dysmyelination	Tanaka R, Iwasaki N, Hayashi M, Nakayama J, Ohto T, Takahashi M, Numano T, Homma K, Hamano K,	Brain Dev. 2012 Mar;34(3):234-7. doi: 10.1016/j.braindev.2011.05.008.	2012
1	RC18-71	13.5 y	F	de novo		46,XX,r(18)(p11.32q23)[43]/47,XX,r(18),+ma r[7]	24052730	Ring autosomes: some unexpected findings	Caba L, Rusu C, Plăiaşu 5th, Gug G, Grămescu M, Bujoran C, Ochiană D, Volosciuc M, Popescu R, Braha E,	Balkan J Med Genet. 2012 Dec;15(2):35-46. doi: 10.2478/bjmg-2013-0005.	2012

1	RC18-72	2 y	F	de novo		46,XX,r(18)(p11.32q21.3)	24052730	Ring autosomes: some unexpected findings			
1	RC18-73	2 m	M	de novo		46,XY,r(18)(p11.3q23)	24052730	Ring autosomes: some unexpected findings			
1	RC18-74 pt 1	10 y	M			46,XY,r(18)	23876976	Single-nucleotide polymorphism array-based characterization of ring chromosome 18	Spreiz A, Guilherme RS, Castellan C, Green A, Rittinger O, Wellek B, Utermann B, Erdel M, Fauth C, Haberlandt E, Kim CA, Kulikowski LD, Meloni VA, Utermann G, Zschocke J, Melaragno MI, Kotzot D.	J Pediatr. 2013 Oct;163(4):1174-8.e3. doi: 10.1016/j.jpeds.2013.06.005. Epub 2013 Jul 19.	2013
1	RC18-74 pt 2	22 y	F		> 22 y	46,XX,r(18)	23876976				
1	RC18-74 pt 3	8 y	F			46,XX,r(18)	23876976				
1	RC18-74 pt 4	14	F			46,XX,r(18)	23876976				
1	RC18-74 pt 5	1.5 y	F			46,XX,r(18)	23876976				
1	RC18-74 pt 6	39 y	F		> 39 y	46,XX,r(18)	23876976				
1	RC18-74 pt 7	11 y	M			46,XY,r(18)	23876976				
1	RC18-74 pt 8	7 y	F			46,XX,r(18)	23876976				
1	RC18-74 pt 9	13.5 y	F			46,XX,r(18)	23876976				
1	RC18-75	2.5 y	M	de novo		46,XY,r(18)(p11.32q21.32)	25635256	Ring chromosome 18: a case report	Heydari S, Hassanzadeh F, Hassanzadeh Nazarabadi M.	Int J Mol Cell Med. 2014 Fall;3(4):287-9.	2014
1	RC18-76	14 y	M			46,XY,r(18)(p11q23)	24909220	A case of ophiasis type of alopecia areata in a patient with ring chromosome 18 syndrome	Kagimoto Y, Mizuashi M, Kikuchi K, Aiba S.	J Dermatol. 2014 Jun;41(6):559-60. doi: 10.1111/1346-8138.12512.	2014
1	RC18-77	23 y	M	mat	> 23 y	46,XY,r(18)(p11q21)[75]/46,XY[25]	24677800	Formation of a familial ring chromosome 18 investigated by SNP-array analysis	Balci S, Zschocke J, Kotzot D, Ergün MA, Spreiz A.	Am J Med Genet A. 2014 Jul;164A(7):1854-6. doi: 10.1002/ajmg.a.36496. Epub 2014 Mar 26.	2014
0	RC18-77 mat					47,XX,r(18)(p11q21)[10]/46,XX[90]	24677800				
1	RC18-78	3 y	M	de novo		46,XY,r(18)(p11.2q23)[97]/45,XY,-18[3]	25668898	Selective IgM deficiency in a boy with ring chromosome 18	Celmeli F, Turkkahraman D, Cetin Z, Mihci E, Yegin O.	J Investig Allergol Clin Immunol. 2014;24(6):442-4.	2014
	RC18-79	5.5 y	F	de novo		46,XX,r(18)	25229966	[Growth hormone deficiency in a girl with ring 18 chromosome syndrome]	Zhang YN, Reheman P, Du HW, Wang LJ.	Zhongguo Dang Dai Er Ke Za Zhi. 2014 Sep;16(9):947-8.	2014
1	RC18-80 case 1	pn, 18 gwks	F		top	46,XX,r(18)[27]/45,XX,-18[5]	26224010	Molecular characterization of ring chromosome 18 by low-coverage next generation sequencing	Ji X, Liang D, Sun R, Liu C, Ma D, Wang Y, Hu P, Xu Z.	BMC Med Genet. 2015 Jul 30;16:57. doi: 10.1186/s12881-015-0206-x.	2015
1	RC18-80 case 2	8 m	F	de novo		46,XX,r(18)	26224010				
0	RC18-81						26468787	Sphincterplasty for Velopharyngeal Insufficiency in the Child Without a Cleft-Palate: Etiologies and Speech Outcomes	Golinko MS, Mason K, Nett K, Riski JE, Williams JK.	J Craniofac Surg. 2015 Oct;26(7):2067-71. doi: 10.1097/SCS.0000000000001967.	2015
0	RC18-82						25339348	Ring 18 molecular assessment and clinical consequences	Carter E, Heard P, Hasi M, Soileau B, Sebold C, Hale DE, Cody JD.	Am J Med Genet A. 2015 Jan;167A(1):54-63. doi: 10.1002/ajmg.a.36822. Epub 2014 Oct 22.	2015
1	RC18-83	nb	F			46,XX,r(18)(p11q22)	27577543	Patchy white matter hyperintensity in ring chromosome 18 syndrome	Anzai M, Arai-Ichinoi N, Takezawa Y, Endo W, Inui T, Sato R, Kikuchi A, Uematsu M, Kure S, Haginoya K.	Pediatr Int. 2016 Sep;58(9):919-22. doi: 10.1111/ped.13043. Epub 2016 Aug 31.	2016
1	RC18-84	2 y	F	de novo		46,XX,r(18)	27448395	Breakpoints and deleted genes identification of ring chromosome 18 in a Chinese girl by whole-genome low-coverage sequencing: a case report study	Yao H, Yang C, Huang X, Yang L, Zhao W, Yin D, Qin Y, Mu F, Liu L, Tian P, Liu Z, Yang Y.	BMC Med Genet. 2016 Jul 22;17(1):49. doi: 10.1186/s12881-016-0307-1.	2016
1	RC18-85	nb	M			46,XY,r(18)	26893613	Ring chromosome 18 in combination with 18q12.1 (DTNA) interstitial microdeletion in a patient with multiple congenital defects	Zlotina A, Nikulina T, Yany N, Moiseeva O, Pervunina T, Grekhov E, Kostareva A.	Mol Cytogenet. 2016 Feb 18;9:18. doi: 10.1186/s13039-016-0229-9. eCollection 2016.	2016
1	RC18-86 case 2	9 y	M			46,XY,r(18)	26947896	Humoral deficiency in thre peadiatric patients with genetic diseases	Campoverde KC, Gean E, Gibert MP et al.,	Allergol Immunopathol. 2016;44(3):257-62	2016
1	RC18-87	nb	F		> 32 y	46,XX,r(18)[14]/46,XX[4]/r-var[2]	29560252	Rheumatoid arthritis in an adult patient with mosaic distal 18q-, 18p- and ring chromosome 18	Chau A, Ramesh KH, Jagannath AD, Arora S.	F1000Res. 2017 Nov 2;6:1940. doi: 10.12688/f1000research.11539.2. eCollection 2017.	2017
1	RC18-88		M			46,XY,r(18)[52]/45,XY-18[3]	31598948	[Molecular cytogenetic diagnosis of a case with ring chromosome 18 syndrome]	Lyu Y, Wang X, Zhang K, Gao M, Ma J, Liu X, Gai Z, Liu Y.	Zhonghua Yi Xue Yi Chuan Xue Za Zhi. 2019 Oct 10;36(10):1010-1014. doi: 10.3760/cma.j.issn.1003-9406.2019.10.015.	2019
1	RC18-89	3 y	F	de novo		46,XX,r(18)(p11.32q22.2)[0.93]/45,XX,-18[0.07]	31788257	Central and peripheral dysmyelination in a 3-year-old girl with ring chromosome 18	Lammert DB, Miedema D, Ochotorena J, Dosa N, Petropoulou K, Lebel RR, Sakonju A.	Clin Case Rep. 2019 Sep 27;7(11):2087-2091. doi: 10.1002/ccr3.2426. eCollection 2019 Nov.	2019
1	RC18-90	7 y	F	de novo		46,XX,r(18)[30]/46,XX,psu idic(18)(p11.2[25]	32972420	Mosaic chromosome 18 anomaly delineated in a child with dysmorphism using a three-pronged cytogenetic techniques approach: a case report	Sheth H, Trivedi S, Liehr T, Patel K, Jain D, Sheth J, Sheth F.	BMC Med Genomics. 2020 Sep 24;13(1):141. doi: 10.1186/s12920-020-00796-9.	2020

1	RC18-91	nb	F	de novo		46,XX,r(18)(p11.31q21.31)	33224015	A Case of Ring Chromosome 18 with Single Umbilical Artery Detected During Prenatal Period	Eras N.	Mol Syndromol. 2020 Nov;11(4):217-222. doi: 10.1159/000509646. Epub 2020 Sep 10.	2020
1	RC18-92	4 y	M			46,XY,r(18)	32209754	Blaschkoid hypermelanosis in a patient with ring 18 chromosome	Varas-Meis E, Delgado-Vicente S, Fernández-Canga P, Rodríguez Prieto MA.	Indian J Dermatol Venereol Leprol. 2020 May-Jun;86(3):316-318. doi: 10.4103/ijdvl.IJDVL_282_18.	2020
1	RC18-93	11 y	F	mat nl		46,XX,r(18)	33829328	Mosaic ring chromosome 18 in a Chinese child with epilepsy: a case report and review of the literature.	Wang J, Xiao L, Wang J, Ding Z, Ni J, Long X.	Neurol Sci. 2021 Dec;42(12):5231-5239	2021
1	RC18-94	20 y	M		> 20 y	46,XY,r(18)	34606568	Anesthetic Management of a Patient With Ring 18 Syndrome	Maekawa M, Yasuda M, Sasaki H	Anesth Prog. 2021 Oct 1;68(3):178-179	2021
1	RC18-95	27 y	F	de novo	> 27 y	46,XX,r(18)(p11.32q22)	34775554	A case report of Ring chromosome 18 with systemic Lupus Erythematosus and Crohn's disease	Rezaeizadeh T, Delshad E, Mansour Samaei N, Gholipour N	Mol Biol Rep. 2022 Feb;49(2):1085-1088. doi: 10.1007/s11033-021-06933-6, Epub2021	2022
**110**	**95**										
1	RC19-1 Case 1	13 y	F	de novo		46,XX[0.78]/46,XX,r(19)[0.22]	5038363	Ring formation of chromosomes nos. 19 and 20	Uchida IA, CC Lin	Cytogenetics 1972;11(3):208-15	1972
1	RC19-2	nb	M	nd		PB: 46,XY[45]/46,XY,r(19)[5], SF[4/50]/PB[8/50]	3233786	Mosaicism for ring 19: a case report	Sybert VP, Bradley CM, Salk D	Clin Genet 34(6):382-5, 1988	1988
1	RC19-3	pn, 17 gwks	F	de novo	stillborn, 21 gwks	AF: 46,XX/46,XX,r(19), PB: r(19)[20/25], SF: r(19)[21/30]	2274494	Ring 19 mosaicism detected during prenatal diagnosis	Gillessen-Kaesbach G, Ngo NT.	Prenat Diagn. 1990 Oct;10(10):683-7. doi: 10.1002/pd.1970101009.	1990
1	RC19-4 case 1	adult	F		> 20 y	PB: 46,XX,r(19),22p+[93]/45,XX,-19,22p+[7] SF: 46,XX,r(19)[86]/46,XX[7]/45,XX,-19[4]/r-var[3]	8454286	High-resolution cytogenetic characterization of telomeric associations in ring chromosome 19	Sawyer JR, Rowe RA, Hassed SJ, Cunniff C.	Hum Genet. 1993 Mar;91(1):42-4. doi: 10.1007/BF00230220.	1993
1	RC19-5	1.2 y	F	mat		46,XX,r(19)[119/121]/46,XX[1]/45,XX,-19[1] mat 46,XX[96]/46,XX,r(19)[4]	8985487	Familial ring (19) chromosome mosaicism: case report and review	Flejter WL, Finlinson D, Root S, Nguyen W, Brothman AR, Viskochil D.	Am J Med Genet. 1996 Dec 18;66(3):276-80. doi: 10.1002/(SICI)1096-[illegible]	1996
1	RC19-6 case 1	1.5 y	F	mat	> 20 y (mat)	46,XX,r(19), mat 46,XX,r(19)[0.20]/46,XX[0.80]	16179226	High resolution microarray CGH and MLPA analysis for improved genotype/phenotype evaluation of two childhood genetic disorder cases: ring chromosome 19 and partial duplication 2q	Hermsen MA, Tijssen M, Acero IH, Meijer GA, Ylstra B, Toral JF.	Eur J Med Genet. 2005 Jul-Sep;48(3):310-8. doi: 10.1016/j.ejmg.2005.04.009.	2005
**6**	**6**										
1	RC20-[1]	10 y	F	de novo	> 26 y	46,XX[0.78]/46,XX,r(20)[0.22]	5038363				
1	RC20-1	4 m	M			46,XY,r(20)[47]/45,XY,-20[3]	4627939	A ring-20 chromosome	Atkins L, Miller WL, Salam M.	J Med Genet. 1972 Sep;9(3):377-80. doi: 10.1136/jmg.9.3.377.	1972
1	RC20-2						305753	[Ring-20 chromosome: a new syndrome]	Jalbert P, Jalbert H, Sele B, Tachker D, Bost M, Meynard R, Pison H.	Ann Genet. 1977 Dec;20(4):258-62.	1977
1	RC20-3	5 y	F		> 21 y	PB/BM: 46,XX,r(20)[0.90]/46,XX[0.09]	926142	The r(20) syndrome	Herva R, Saarinen I, Leikkonen L.	J Med Genet. 1977 Aug;14(4):281-3. doi: 10.1136/jmg.14.4.281.	1977
1	RC20-4	1 y	M	de novo		46,XY,r(20)[21]/46,XY[3]	475436	Ring 20 chromosome in a child with seizures, minor anomalies, and retardation	Stewart JM, Cavanagh N, Hughes DT.	Arch Dis Child. 1979 Jun;54(6):477-9. doi: 10.1136/adc.54.6.477.	1979
1	RC20-5	8 m	F	de novo		46,XX,r(20)	2418815	A case of ring 20 chromosome with cardiac and renal anomalies	Burnell RH, Stern LM, Sutherland GR.	Aust Paediatr J. 1985 Nov;21(4):285-6. doi: 10.1111/j.1440-1754.1985.tb00167.x.	1985
1	RC20-6	nb	M	de novo		46,XY,r(20)(p13q13)	3612710	Ring 20 chromosome phenotype	Porfirio B, Valorani MG, Giannotti A, Sabetta G, Dallapiccola B.	J Med Genet. 1987 Jun;24(6):375-7. doi: 10.1136/jmg.24.6.375.	1987
1	RC20-7	2 y	M	mat	> 38 y (mat)	46,XY,r(20)[PB0.88,SF0.66]/46,XY	2777254	Familial ring (20) chromosomal mosaicism	Back E, Voiculescu I, Brünger M, Wolff G.	Hum Genet. 1989 Sep;83(2):148-54. doi: 10.1007/BF00286708.	1989
0	RC20-7 sister	2 y	F	mat		46,XX,r(20)[PB0.67,SF0.22]/46,XX; mat: 46,XX,r(20)[PB0.31,SF0.34]/46,XX	2777254				
1	RC20-8						2757359	A case of the ring 20 syndrome	Thomsen SG, Petersen MB, Andersen EA, ostergaard GZ, Lindenberg S.	Ann Genet. 1989;32(2):111-3.	1989
1	RC20-9	8.5 y	M	de novo		46,XY,r(20)(p13q13.3)	1605251	Ring chromosome 20 and possible assignment of the structural gene encoding human carboxypeptidase-L to the distal segment of the long arm of chromosome 20	Halal F, Chitayat D, Parikh H, Rosenblatt B, Tranchemontagne J, Vekemans M, Potier M.	Am J Med Genet. 1992 Jun 1;43(3):576-9. doi: 10.1002/ajmg.1320430314.	1992
1	RC20-10	5 m	M	de novo		46,XY,r(20)	8403451	Ring chromosome 20 with loss of telomeric sequences detected by multicolour PRINS	Brandt CA, Kierkegaard O, Hindkjaer J, Jensen PK, Pedersen S, Therkelsen AJ.	Clin Genet. 1993 Jul;44(1):26-31. doi: 10.1111/j.1399-0004.1993.tb03837.x.	1993

1	RC20-11	7 y	F	de novo		46,XX/46,XX,r(20)(p13q13)	8132117	Ring chromosome 20 mosaicism in a girl with complex partial seizures	Holopainen I, Penttinen M, Lakkala T, Aärimaa T.	Dev Med Child Neurol. 1994 Jan;36(1):70-3. doi: 10.1111/j.1469-8749.1994.tb11768.x.	1994
1	RC20-12	3.3 y	M	de novo		46,XY/46,XY,r(20)(p13q13.33)	7635104	Nonphotosensitive video game-induced partial seizures	Takahashi Y, Shigematsu H, Kubota H, Inoue Y, Fujiwara T, Yagi K, Seino M.	Epilepsia. 1995 Aug;36(8):837-41. doi: 10.1111/j.1528-1157.1995.tb01623.x.	1995
1	RC20-13 pt 1	8 y	F			46,XX,r(20)(p13q13.33)[4]/46,XX[16]	9217679	Ring chromosome 20 and nonconvulsive status epilepticus. A new epileptic syndrome	Inoue Y, Fujiwara T, Matsuda K, Kubota H, Tanaka M, Yagi K, Yamamori K,	Brain. 1997 Jun;120 (Pt 6):939-53. doi: 10.1093/brain/120.6.939.	1997
1	RC20-13 pt 2	3.3 y	M			46,XY,r(20)(p13q13.33)[16]/46,XY[14]	9217679				
1	RC20-13 pt 3	14 y	F		> 21 y	46,XX,r(20)(p13q13.33)[5]/46,XX[15]	9217679				
1	RC20-13 pt 4	7 y	F		> 28 y	46,XX,r(20)(p13q13.33)[8]/46,XX[12]	9217679				
1	RC20-13 pt 5	7 y	F		> 31 y	46,XX,r(20)(p13q13.3)[13]/46,XX[36]	9217679				
1	RC20-13 pt 6	11 y	M		> 25 y	46,XY,r(20)(p13q13.33)[3]/46,XY[27]	9217679				
1	RC20-14						9935297	[A new epileptic syndrome: ring chromosome 20--interhemispheric peak delay of spikes]	Kamuro K, Yoshimi S, Morikawa T.	No To Hattatsu. 1998 Sep;30(5):431-2.	1998
1	RC20-15	3 y	F		> 24 y	46,XX,r(20)(p13q13.3)[0.87]/46,XX[0.13]	9682935	A study of ring 20 chromosome karyotype with epilepsy	Yamadera H, Kobayashi K, Sugai K, Suda H, Kaneko S.	Psychiatry Clin Neurosci. 1998 Feb;52(1):63-8. doi: 10.1111/j.1440-1819.1998.tb00974.x.	1998
1	RC20-15.5 pt 1	5 y	M	mat	> 44 y (mat)	46,XY,r(20)[0.83]/46,XX[0.17];<br>mat 46,XX,r(20)[0.12]/46,XX[0.88]	9738673	chromosome 20 ring: a chromosomal disorder associste particular electroclinical pattern.	Canevini MP, Sgro V, Zuffardi O, Canger R,	Epilepsia 1998, 39:942-951.	1998
1	RC20-15.5 pt 3	21 y	M		> 21 y	46,XY,r(20)[0.13]/46,XY[0.67]	9738673				
3	RC20-16						10937160	Non-convulsive status in the ring chromosome 20 syndrome: a video illustration of 3 cases	Petit J, Roubertie A, Inoue Y, Genton P.	Epileptic Disord. 1999 Dec;1(4):237-41.	1999
2	RC20-17						10743013	[Ring chromosome 20: an identifiable epileptic syndrome]	Roubertie A, Petit J, Genton P.	Rev Neurol (Paris). 2000 Feb;156(2):149-53.	2000
1	RC20-18 pt1	11 y	F			46,XX,r(20)[0.68]	11571346	Ring chromosome 20 epilepsy syndrome in children: electroclinical features	Augustijn PB, Parra J, Wouters CH, Joosten P, Lindhout D, van Emde Boas	Neurology. 2001 Sep 25;57(6):1108-11. doi: 10.1212/wnl.57.6.1108.	2001
1	RC20-18 pt2	3 y	F			46,XX,r(20)[0.12]	11571346				
1	RC20-18 pt3	11 y	M			46,XY,r(20)[0.14]	11571346				
1	RC20-18 pt4	8 y	F			46,XX,r(20)[0.28]	11571346				
1	RC20-19						11310277	[Ring chromosome 20: an epileptic channel disorder?]	Serrano-Castro PJ, Aguilar-Castillo MJ, Olivares-Romero J, Jiménez-Machado	Rev Neurol. 2001 Feb 1-15;32(3):237-41.	2001
1	RC20-20	2 y	M	de novo		46,XY,r(20)(p13q13.3)	11879375	Ring 20 chromosome syndrome with epilepsy and dysmorphic features: a case report	García DM, Ortiz R, Gómez A, Barriuso E.	Epilepsia. 2001 Dec;42(12):1607-10. doi: 10.1046/j.1528-	2001
1	RC20-21	3 m	F			46,XX,r(20)	12546436	Intractable epilepsy with ring chromosome 20 syndrome treated with vagal nerve stimulation: case report and review of the literature	Chawla J, Sucholeiki R, Jones C, Silver K.	J Child Neurol. 2002 Oct;17(10):778-80. doi: 10.1177/08830738020170101805.	2002
1	RC20-22	9 y	M	de novo		46,XY,r(20)(p13q13.3)[0.90]/r-var[0.10]	12244405	Epilepsy and ring chromosome 20: case report	Gomes Mda M, Lucca I, Bezerra SA, Llerena J Jr, Moreira DM.	Arq Neuropsiquiatr. 2002 Sep;60(3-A):631-5. doi: 10.1590/s0004-	2002
0	RC20-23						11914429	Ring chromosome 20 epilepsy syndrome in children: electroclinical features	Serrano-Castro PJ.	Neurology. 2002 Mar 26;58(6):987; author reply 987. doi: 10.1212/wnl.58.6.987.	2002
1	RC20-24						14593631	[Ring chromosome 20, hypersensitivity to valproate and hyperammonemic encephalopathy]	Ortiz-Sáenz de Santa María MR, Barriuso-Pérez E, Soto-Alvarez MI,	Rev Neurol. 2003 Oct 16-31;37(8):733-5.	2003
0	RC20-25						15249613	PET evidence for a role of the basal ganglia in patients with ring chromosome 20 epilepsy	Biraben A, Semah F, Ribeiro MJ, Douaud G, Remy P, Depaulis A.	Neurology. 2004 Jul 13;63(1):73-7. doi: 10.1212/01.wnl.0000132840.40838.13.	2004
1	RC20-26						15131740	[Ring chromosome 20: a distinctive syndrome identifiable by electroclinical diagnosis]	Gonzalez-Delgado M, Salas J, Hernando I, Calleja S, Hernandez C.	Neurologia. 2004 May;19(4):215-9.	2004
1	RC20-27	8 y	M			46,XY,r(20)(p13q13.3)[2]/46,XY[28]	14966172	Ictal magnetoencephalographic study in a patient with ring 20 syndrome	Tanaka N, Kamada K, Takeuchi F.	J Neurol Neurosurg Psychiatry. 2004 Mar;75(3):488-90. doi:	2004
1	RC20-28						16164241	[Continuous midazolam infusion for refractory nonconvulsive status epilepticus in children]	Nobutoki T, Sugai K, Fukumizu M, Hanaoka S, Sasaki M.	No To Hattatsu. 2005 Sep;37(5):369-73.	2005
0	RC20-29						16097967	Regarding: 'Mosaicism and seizure onset in ring chromosome 20 syndrome'	Serrano-Castro PJ, Aguilar Castillo MJ.	Acta Neurol Scand. 2005 Sep;112(3):202; author reply 203.	2005
1	RC20-30 pt 1	12 y	F		> 25 y	46,XX,r(20)(p13q13)[0.20]/46,XX[0.80]	16128150	Ring chromosome 20 with nonconvulsive status epilepticus: electroclinical correlation of a rare epileptic syndrome	Locharernkul C, Ebner A, Promchainant C.	Clin EEG Neurosci. 2005 Jul;36(3):151-60. doi: 10.1177/155005940503600305.	2005
1	RC20-30 pt 2	15 y	F		> 37 y	46,XX,r(20)(p13q13)[0.20]/46,XX[0.80]	16128150				

1	RC20-31	1.5 y	M	de novo		46,XY,r(20)[200]	15892377	Ring chromosome 20 syndrome with intractable epilepsy	Alpman A, Serdaroglu G, Cogulu O, Tekgul H, Gokben S, Ozkinay F.	Dev Med Child Neurol. 2005 May;47(5):343-6. doi: 10.1017/s0012162205000642.	2005
1	RC20-32	17 y	F		> 36 y	46,XX,r(20)[0.13]/45,XX,-20[0.03]/46,XX[0.84]	15691292	Mosaicism and phenotype in ring chromosome 20 syndrome	Nishiwaki T, Hirano M, Kumazawa M, Ueno S.	Acta Neurol Scand. 2005 Mar;111(3):205-8. doi:	2005
1	RC20-33	5 y	F		> 26 y	46,XX/46,XX,r(20)(p13q13.3)	16835934	Mosaic ring 20 with no detectable deletion by FISH analysis: Characteristic seizure disorder and literature review	Zou YS, Van Dyke DL, Thorland EC, Chhabra HS, Michels VV, Keefe JG, Lega MA, Feely MA, Uphoff TS, Jalal	Am J Med Genet A. 2006 Aug 1;140(15):1696-706. doi: 10.1002/ajmg.a.31332.	2006
1	RC20-34	21 y	F		> 46 y	46.XX.r(20)[0.50]/46,XX[0.25]/r-var[0.25]	16806995	Electroclinical evolution in ring chromosome 20 epilepsy syndrome: a case with severe phenotypic features followed for 25 years	de Falco FA, Olivieri P, de Falco A, Concolino D, Battaglia F, Verardi R, Grande G, Stabile M.	Seizure. 2006 Sep;15(6):449-53. doi: 10.1016/j.seizure.2006.03.004. Epub 2006 Jun 27.	2006
1	RC20-35 case 1	6 y	M			46,XY,r(20)[0.43]	16529619	Early pattern of epilepsy in the ring chromosome 20 syndrome	Ville D, Kaminska A, Bahi-Buisson N, Biraben A, Plouin P, Telvi L, Dulac O,	Epilepsia. 2006 Mar;47(3):543-9. doi: 10.1111/j.1528-	2006
1	RC20-35 case 2	5 y	M		> 24 y	46,XY,r(20)[0.77]	16529619				
1	RC20-35 case 3	12 Y	F			46,XX,r(20)[0.40]	16529619				
1	RC20-35 case 4	5 y	M			46,XY,r(20)[0.80]	16529619				
1	RC20-35 case 5	8 y	M			46,XY,r(20)[0.22]	16529619				
1	RC20-35 neonatal	nb	M			46,XY,r(20)	16529619				
1	RC20-36	5 y	M			46,XY,r(20)[0.80]/46,XY[0.20]	16359603	Epilepsy responds to vagus nerve stimulation in ring chromosome 20 syndrome	Parr JR, Pang K, Mollett A, Zaiwalla Z, Selway R, McCormick D, Jayawant S.	Dev Med Child Neurol. 2006 Jan;48(1):80; author reply 80. doi:	2006
1	RC20-37	2 y	F			46,XX,r(20)(p13q13.3)[14]/46,XX[6]	17851150	Ring chromosome 20 syndrome without deletions of the subtelomeric and CHRNA4--KCNQ2 genes	Elghezal H, Hannachi H, Mougou S, Kammoun H, Triki C, Saad A.	Eur J Med Genet. 2007 Nov-Dec;50(6):441-5. doi:	2007
1	RC20-38	24 y	F	de novo	> 42 y	46,XX,r(20)(p13q13.3)[0.10]/46,XX[0.90]	17079116	More severe epilepsy and cognitive impairment in the offspring of a mother with mosaicism for the ring 20 chromosome	Herrgård E, Mononen T, Mervaala E, Kuusela L, Aikiä M, Stenbäck U, Pääkkönen L, Airaksinen RL, Kälviäinen	Epilepsy Res. 2007 Jan;73(1):122-8. doi: 10.1016/j.eplepsyres.2006.09.006.	2007
0	RC20-38 daughter	7 y	F	mat		46,XX,r(20)(p13q13.3)[0.40]/46,XX[0.60]	17079116				
0	RC20-38 son	5 y	M	mat		46,XY,r(20)[0.40]/46,XY[0.60]	17079116				
1	RC20-39	3 y	M		d. 13 y cardiovascular collapse	46,XY,r(20)	19017565	Refractory and lethal status epilepticus in a patient with ring chromosome 20 syndrome	Jacobs J, Bernard G, Andermann E, Dubeau F, Andermann F.	Epileptic Disord. 2008 Dec;10(4):254-9. doi: 10.1684/epd.2008.0212.	2008
1	RC20-40 pt 1	7.5 y	F			46,XX,r(20)(p13q13.3)	19583784	Ring chromosome 20 syndrome: a link between epilepsy onset and neuropsychological impairment in three children	Vignoli A, Canevini MP, Darra F, La Selva L, Fiorini E, Piazzini A, Lazzarotto F, Zucca C, Dalla Bernardina B.	Epilepsia. 2009 Nov;50(11):2420-7. doi: 10.1111/j.1528-1167.2009.02176.x. Epub 2009 Jul	2009
1	RC20-40 pt 2	9.5 y	F			46,XX,r(20)[53]/46,XX[47]	19583784				
1	RC20-40 pt 3	4 y	F		> 20 y	46,XX,r(20)[26]/46,XX[74]	19583784				
5	RC20-41						20927024	Exploring dopaminergic activity in ring chromosome 20 syndrome: a SPECT study	Del Sole A, Chiesa V, Lucignani G, Vignoli A, Giordano L, Lecchi M,	Q J Nucl Med Mol Imaging. 2010 Oct;54(5):564-9.	2010
1	RC20-42 case BV	12 y	F			46,XX,r(20)[0.42]	20939888	Genetic investigations on 8 patients affected by ring 20 chromosome syndrome	Giardino D, Vignoli A, Ballarati L, Recalcati MP, Russo S, Camporeale N, Marchi M, Finelli P, Accorsi P,	BMC Med Genet. 2010 Oct 12;11:146. doi: 10.1186/1471-2350-11-146.	2010
1	RC20-42 case PE	14 y	F			46,XX,r(20)[0.34]	20939888				
1	RC20-42 case MM	pn	F		CtB	46,XX,r(20)[0.30]	20939888				
1	RC20-43	5 y	F			PB: 46,XX,r(20)[0.34], SF: 46,XX,r(20)(p13q13.3)[0.08]	22000318	New association between ring chromosome 20 syndrome and hypomelanosis of Ito	Cappanera S, Passamonti C, Zamponi N.	Pediatr Neurol. 2011 Nov;45(5):341-3. doi: 10.1016/j.pediatrneurol.2011.08.006.	2011
1	RC20-44	13 y	F			46,XX,r(20)[0.77]	21397468	[Polymorphic expression of epilepsy and cognitive impairment in ring chromosome 20 syndrome]	Villéga F, Ngayap H, Espil-Taris C, Husson M, Rooryck-Thambo C, Arveiler B, Lacombe D, Pédespan JM.	Arch Pediatr. 2011 Apr;18(4):394-6. doi: 10.1016/j.arcped.2010.12.025.	2011
0	RC20-45						20972251	Molecular analysis of ring chromosome 20 syndrome reveals two distinct groups of patients	Conlin LK, Kramer W, Hutchinson AL, Li X, Riethman H, Hakonarson H, Mulley JC, Scheffer IE, Berkovic SF, Hosain SA,	J Med Genet. 2011 Jan;48(1):1-9. doi: 10.1136/jmg.2010.080382. Epub 2010 Oct 23.	2011
1	RC20-46	pn	F		top	Twin: 46,XY and 46,XX,r(20)[44]/45,XX,-20[6]	23272278	Prenatal diagnosis of a fetus with a ring chromosome 20 characterized by array-CGH	Cignini P, Dugo N, Giorlandino C, Gauci R, Spata A, Capriglione S, Cafà EV.	J Prenat Med. 2012 Oct;6(4):72-3.	2012
1	RC20-47	adult	F		> 20 y	46,XX,r(20)	23157113	[A case of epilepsy with ring chromosome 20 syndrome]	Tanaka A, Ohtake M, Yoshimi T, Suzuki T, Abe I, Iwasaki H, Sue H, Kaito K.	Rinsho Byori. 2012 Sep;60(9):847-52.	2012
1	RC20-48	9 y	F			46,XX,r(20)[53]/46,XX[47]	22738216	Ictal involvement of the nigrostriatal system in subtle seizures of ring chromosome 20 epilepsy	Meletti S, Vignoli A, Benuzzi F, Avanzini P, Ruggieri A, Pugnaghi M, Nichelli P,	Epilepsia. 2012 Aug;53(8):e156-60. doi: 10.1111/j.1528-	2012
1	RC20-49	9 y	F			46,XX,r(20)[12]/46,XX[38]	22591830	Gelastic seizures in ring chromosome 20 syndrome: a case report with video illustration	Dimova P, Boneva I, Todorova A, Minotti L, Kahane P.	Epileptic Disord. 2012 Jun;14(2):181-6. doi: 10.1684/epd.2012.0508.	2012
1	RC20-50	9 y	M			46,XY,r(20)[15]/46,XY[35]	21858587	Cognitive impairment and abnormal behaviour related to ring chromosome 20 aberration	Gahr M, Kerling F, Ludolph A, Plener P.	J Autism Dev Disord. 2012 Jun;42(6):1146-8. doi: 10.1007/s10803-011-1346-2.	2012

1	RC20-51 pt1	4 y	F			46,XX,r(20)[25]	22424860	Ring chromosome 20 syndrome: electroclinical description of six patients and review of the literature	Elens I, Vanrykel K, De Waele L, Jansen K, Segeren M, Van Paesschen W, Ceulemans B, Boel M, Frijns JP, Buyse	Epilepsy Behav. 2012 Apr;23(4):409-14. doi: 10.1016/j.yebeh.2012.02.008. Epub	2012
1	RC20-51 pt2	6 y	M			46,XY,r(20)[0.40]	22424860				
1	RC20-51 pt3	6 y	M		> 53 y	46,XY,r(20)[0.16]	22424860				
1	RC20-51 pt4	16 y	F		> 66 y	46,XX,r(20)[0.18]/45,X,-20[0.04]	22424860				
1	RC20-51 pt5	13 y	M		> 22 y	36,XY,r(20)[0.13]	22424860				
1	RC20-51 pt6	5 y	M			46,XY,r(20)	22424860				
1	RC20-52	17 y	F			46,XX,r(20)	22246017	Frontal motor seizure following non-convulsive status epilepticus in ring chromosome 20	Kamoun FF, Ellouz EJ, Hsairi IG, Triki CC.	Neurosciences (Riyadh). 2012 Jan;17(1):74-7.	2012
1	RC20-53 case 1	5 y	M			46,XY,r(20)[0.25]/46,XY[0.75]	22000954	The evolving electroclinical syndrome of "epilepsy with ring chromosome 20"	Radhakrishnan A, Menon RN, Hariharan S, Radhakrishnan K.	Seizure. 2012 Mar;21(2):92-7. doi: 10.1016/j.seizure.2011.09.009. [illegible]	2012
1	RC20-53 case 2	2.5 y	M			46,XY,r(20)[0.06]/46,XY[0.94]	22000954				
1	RC20-53 case 3	10 y	M		> 20 y	46,XY,r(20)[0.40]/46,XY[0.60]	22000954				
0	RC20-54						24330998	[Ring chromosome 20 syndrome]	Chen CH, Wu HS.	Zhonghua Er Ke Za Zhi. 2013 Sep;51(9):710-2.	2013
1	RC20-55	6 y	F			46,XX,r(20)(p13q13.3)[11]/46,XX[9]	23916860	Ring chromosome 20: a pediatric potassium channelopathy responsive to treatment with ezogabine	Walleigh DJ, Legido A, Valencia I.	Pediatr Neurol. 2013 Nov;49(5):368-9. doi: 10.1016/j.pediatrneurol.2013.06.005. Epub 2013 Aug 2.	2013
1	RC20-56 no.1	2.5 y	F			46,XX,r(20)[0.29]	23731915	Sleep in ring chromosome 20 syndrome: a peculiar electroencephalographic pattern	Zambrelli E, Vignoli A, Nobili L, Didato G, Mastrangelo M, Turner K, Canevini	Funct Neurol. 2013 Jan-Mar;28(1):47-53.	2013
1	RC20-56 no.2	6 y	F			46,XX,r(20)[0.33]	23731915				
1	RC20-56 no.3	9 y	F			46,XX,r(20)[0.53]	23731915				
1	RC20-56 no.4	21 y	M		> 34 Y	46,XY,r(20)[0.13]	23731915				
1	RC20-56 no.5	5 y	M		> 30 y	46,XY,r(20)[0.83]	23731915				
1	RC20-56 no.6	11 y	F		> 59 y	46,XX,r(20)[0.12]	23731915				
1	RC20-57	5 y	F			46,XX,r(20)(p13q13.3)[22]/46,XX[28]	23188914	Two siblings with similar phenotypes: one of them had ring 20 chromosome	Tezer FI, Aktas D, Alikasifoglu M, Saygi S.	Clin EEG Neurosci. 2013 Jan;44(1):58-61. doi: 10.1177/1550059412451700. Epub 2012 Nov 27.	2013
1	RC20-58						25391199	Rare epileptic syndrome of ring chromosome 20 with epileptic encephalopathy: a case report	Wechapinan T, Sri-Udomkajorn S, Suwannachote S.	J Med Assoc Thai. 2014 Jun;97 Suppl 6:S239-42.	2014
1	RC20-59	14 y	M			46,XY[23]/46,XY,r(20)(p13q13.3)[25]	24819941	[Electroclinical characteristics of a patient with ring chromosome 20 syndrome]	Vega-Zelaya L, Alonso-Cerezo C, Quesada JF, Sola RG, Pastor J.	Rev Neurol. 2014 May 16;58(10):450-4.	2014
0	RC20-60						24483620	Epilepsy-related brain networks in ring chromosome 20 syndrome: an EEG-fMRI study	Vaudano AE, Ruggieri A, Vignoli A, Avanzini P, Benuzzi F, Gessaroli G, Nichelli PF, Darra F, Cantalupo G,	Epilepsia. 2014 Mar;55(3):403-13. doi: 10.1111/epi.12539. Epub 2014 Jan 31.	2014
0	RC20-61						23968845	Low frequency mu-like activity characterizes cortical rhythms in epilepsy due to ring chromosome 20	Avanzini P, Vaudano AE, Vignoli A, Ruggieri A, Benuzzi F, Darra F, Mastrangelo M, Dalla Bernardina B,	Clin Neurophysiol. 2014 Feb;125(2):239-49. doi: 10.1016/j.clinph.2013.07.009. Epub	2014
1	RC20-62	6 y	F		> 43 y	46,XX,r(20)	26062827	[Stimulation of the centromedian nucleus in refractory epilepsy associated to ring chromosome 20]	Arévalo-Sáenz A, Torres CV, Pastor J, Alonso-Cerezo C, Sola RG.	Rev Neurol. 2015 Jun 16;60(12):548-52.	2015
0	RC20-63						27816898	Epilepsy in ring chromosome 20 syndrome	Vignoli A, Bisulli F, Darra F, Mastrangelo M, Barba C, Giordano L, Turner K, Zambrelli E, Chiesa V, Bova S, Fiocchi I, Peron A, Naldi I, Milito G,	Epilepsy Res. 2016 Dec;128:83-93. doi: 10.1016/j.eplepsyres.2016.10.004. Epub 2016 Oct 24.	2016
0	RC20-64						27009934	Long-term EEG in patients with the ring chromosome 20 epilepsy syndrome	Freire de Moura M, Flores-Guevara R, Gueguen B, Biraben A, Renault F.	Epilepsia. 2016 May;57(5):e94-6. doi: 10.1111/epi.13352. Epub 2016 Mar 24.	2016
1	RC20-65	7 y	F			46,XX,r(20)(p13q13.3)[23]/45,XX,-20[1]/46,XX[6]	26980640	Refractory and severe status epilepticus in a patient with ring chromosome 20 syndrome	Hirano Y, Oguni H, Nagata S.	Brain Dev. 2016 Sep;38(8):746-9. doi:	2016
1	RC20-66	9 y	F			46,XX,r(20)(p13q13.3)[23/169]	27066580	Co-occurrence of 16p13.11 microdeletion and ring chromosome 20 syndrome	Rodan LH, Zak M, Stavropoulos J, Joseph-George AM, Minassian BA.	Neurol Genet. 2016 Jan 14;2(1):e43. doi:	2016
1	RC20-67	17 y	F		> 25 y	46,XX,r(20) mos	26240087	Significant Improvements of EEG and Clinical Findings With Oral Lacosamide in a Patient With Ring Chromosome 20	Onder H, Tezer FI.	Clin EEG Neurosci. 2016 Oct;47(4):330-332. doi: 10.1177/1550059415593428. Epub 2015 Aug 2.	2016
1	RC20-68	10 m	M	de novo		46,XY,r(20)(p13q13.3)	28482393	[A patient with ring chromosome 20 syndrome and AGTR2 polymorphisms]	Yi Z, Pan H, Li L.	Zhonghua Er Ke Za Zhi. 2017 May 4;55(5):388-389. doi:	2017
1	RC20-69	10 y	F		> 35 y	46,XX,r(20)(p13q13)[0.30]/46,XX[0.70]	28181065	Phenotypical heterogeneity of morpheic seizures in ring chromosome 20 syndrome: a videopolysomnographic evidence.	Giuliano L, Fatuzzo D, Mainieri G, Sofia V, Zappia M	Neurol Sci. 2017 May;38(5):925-926. doi: 10.1007/s10072-017-2838-4. Epub 2017 Feb 8. PMID:	2017
1	RC20-70	6 y	F			46,XX,r(20)[0.20]	30455928	Lithium improved behavioral and epileptic symptoms in an adolescent with ring chromosome 20 and bipolar disorder not otherwise specified	Inal A, Chaumette B, Soleimani M, Guerrot AM, Goldenberg A, Lebas A, Gerardin P, Ferrafiat V.	Clin Case Rep. 2018 Oct 12;6(11):2234-2239. doi: 10.1002/ccr3.1796. eCollection 2018 Nov.	2018

1	RC20-71 pt 1	6 y	F			46,XX,r(20)[0.84]	29414555	Specificity of electroclinical features in the diagnosis of ring chromosome 20	Gago-Veiga AB, Toledano R, García-Morales I, Pérez-Jiménez MA, Bernar J,	Epilepsy Behav. 2018 Mar;80:215-220. doi:	2018
1	RC20-71 pt 2	2 y	F			46,XX,r(20)[0.70]	29414555				
1	RC20-71 pt 3	12 y	F		> 30 y	46,XX,r(20)[0.22]	29414555				
1	RC20-71 pt 4	13 y	F		> 27 y	46,XX,r(20)[0.30]	29414555				
1	RC20-71 pt 5	9 y	F			46,XX,r(20)[0.46]	29414555				
1	RC20-71 pt 6	4 y	M			46,XY,r(20)[0.44]	29414555				
1	RC20-72	2 y	F	mat	> 20 y (mat)	46,XX,r(20)[0.50], mat 46,XX,r(20)[0.12]	30385235	A further case of familial ring chromosome 20 mosaicism - molecular characterization of the ring and review of the literature	Unterberger I, Dobesberger J, Schober H, Krabichler B, Lamina C, Schatz U, Zschocke J, Luef G, Kotzot D, Fauth C.	Eur J Med Genet. 2019 Nov;62(11):103564. doi: 10.1016/j.ejmg.2018.10.016. Epub	2019
1	RC20-73 case 1	6 m	M			46,XY[0.88]/46,XY,r(20)[0.12]	33623285	Epilepsy in Ring Chromosome 20 Syndrome Might Have Variable Clinical Features	Patil AA, Vinayan KP, Roy AG.	Ann Indian Acad Neurol. 2020 Sep-Oct;23(5):718-722. doi:	2020
1	RC20-73 case 2	6 m	F			46,XX,r(20)	33623285				
1	RC20-74 case 1	11 y	F			46,XX,r(20)	32301722	Praxis-induced reflex seizures in two Japanese cases with ring chromosome 20 syndrome	Yamagishi H, Goto M, Osaka H, Kuwajima M, Muramatsu K, Yamagata	Epileptic Disord. 2020 Apr 1;22(2):214-218. doi:	2020
1	RC20-74 case 2	6 y	M			46,XY,r(20)	32301722				
1	RC20-75	7 y	M			46,XY,r(20)(p13q13.3)[4]/46,XY[26]	32247529	Improvement of epilepsy with lacosamide in a patient with ring chromosome 20 syndrome	Tayama T, Mori T, Goji A, Toda Y, Kagami S.	Brain Dev. 2020 Jun;42(6):473-476. doi:	2020
1	RC20-76	nb	F	de novo		46,XX,r(20)	32082653	Candidate Genes Associated with Delayed Neuropsychomotor Development and Seizures in a Patient with Ring Chromosome 20	Corrêa T, Venâncio AC, Galera MF, Riegel M.	Case Rep Genet. 2020 Jan 21;2020:5957415. doi: 10.1155/2020/5957415. eCollection	2020
1	RC20-77	10 y	F		> 21 y	46,XX,t(1;12)(q21;q11),r(20)[14/25]	35002161	A Case of Drug-resistant Epilepsy Associated with Ring Chromosome 20	Balabhadra A, Parekh M, Patil A	Ann Indian Acad Neurol. 2021 Sep-Oct;24(5):805-807	2021
1	RC20-TECH pt 1	9 y	M		> 34 y	46,XY,r(20)	33207017	Transcriptome analysis of a ring chromosome 20 patient cohort	Myers KA, Bennett MF, Hildebrand MS, Coleman MJ, Zhou G, Hollingsworth G, Cairns A, Riney K, Berkovic SF, Bahlo	Epilepsia. 2021 Jan;62(1):e22-e28. doi: 10.1111/epi.16766. Epub 2020 Nov 18.	2021
1	RC20-TECH pt 2	6 y	F			46,XX,r(20)[0.73]	33207017				
1	RC20-78	7 y	M			46,XY,r(20)[0.62]	35196643	Intravenous methylprednisolone is a potential add on therapy for Ring chromosome 20 syndrome	Kishore VK, Viswanathan LG, Asranna A, Kenchiah R, Chowdary M R, Sinha S.	Seizure. 2022 Feb 5;96:118-120.	2022
**118**	**80**										
1	RC21-1	10 y	F			46,XX,r(21)	1242523	[A dysplasia-epilepsy syndrome in a patient with ring chromosome 21]	Kunze J, Doose H, Tolksdorf M.	Neuropadiatrie. 1975 Nov;6(4):398-402. doi: 10.1055/s-0028-1091680.	1975
1	RC21-2						1154140	Clinical and cytogenetic aspects of the 21 deletion syndrome	Gericke GS, Steyn MF, Retief AE, Thom JC, Van Niekerk WA.	S Afr Med J. 1975 Jun 7;49(24):959-64.	1975
1	RC21-3					46,XY,r(21)	1004994	[Ring chromosome 21. A new case]	Larget-Piet L, Berthelot J, Guittet J, Hamon A, Larget-Piet A, Rouchy R.	Pediatrie. 1976 Sep;31(6):539-49.	1976
1	RC21-4						955958	[Ring chromosome 21]	Neuhäuser G.	Hippokrates. 1976 Aug;47(3):246-7.	1976
1	RC21-5						278545	Missing X chromosome and ring chromosome 21 in a case of acute myelomonocytic leukemia	Olinici CD, Marinca E, Macavei I, Dobay O.	Arch Geschwulstforsch. 1978;48(3):202-4.	1978
1	RC21-6	nb	M	de novo		PB/BM/SF: 46,XY,r(21)/45,XY,-21	7332027	Analysis of banding patterns in a case of ring chromosome 21	Richer CL, Fitch N, Sitahal S, Murer-Orlando M, Jean P.	Am J Med Genet. 1981;10(4):323-31. doi: 10.1002/ajmg.1320100404.	1981
1	RC21-7	2.5 y	M				7037042	Acute megakaryoblastic leukaemia associated with intrinsic platelet dysfunction and constitution ring 21 chromosome in a young boy	Pui CH, Williams DL, Scarborough V, Jackson CW, Price R, Murphy S.	Br J Haematol. 1982 Feb;50(2):191-200. doi: 10.1111/j.1365-2141.1982.tb01909.x.	1982
1	RC21-8 pt 1/2	pn	M	mat	CP, > 28 y (mat)	46,XY,r(21)	6228144	Ring chromosome 21 in phenotypically apparently normal persons: report of two families from Switzerland and Italy	Schmid W, Tenconi R, Baccichetti C, Caufin D, Schinzel A.	Am J Med Genet. 1983 Nov;16(3):323-9. doi: 10.1002/ajmg.1320160305.	1983
1	RC21-8 pt 3/4	nb	F	mat	> 38 y (mat)	46,XX[0.97]/47,XX,+r(21)(p11q22.3)[0.03] mat: 46,XX,r(21)(p11q22.3)[48]	6228144				
1	RC21-9						6680430	[Description of a case of ring chromosome 21 and pericentric inversion of Y chromosome]	Ponzio G, Carozzi F, Dragone E, Spada A, Brignone S, De Marchi M, Carbonara	Pathologica. 1983;75 Suppl:276-9.	1983
1	RC21-10	25 y	F			46,XX,r(21)	6745921	Ring chromosome 21 in a healthy woman with three spontaneous abortions	Rhomberg K.	Hum Genet. 1984;67(1):120. doi: 10.1007/BF00270571.	1984
1	RC21-11	pn	M	de novo	cp	46,XY,r(p13q22.3)/45,XY,-21	6510909	Prenatal detection of an unstable ring 21 chromosome	Stetten G, Sroka B, Corson VL, Boehm CD.	Hum Genet. 1984;68(4):310-3. doi: 10.1007/BF00292590.	1984
1	RC21-12	nb	F			46,XX,r(21)/45,XX,-21	6335374	Ring chromosome 21. Observation in a female infant	Carlo Stella N, Barberi I, Corrado F, Triolo O.	Ann Genet. 1984;27(4):249-51.	1984
1	RC21-13						6331791	Ring chromosome 21 in a normal female	Kleczkowska A, Fryns JP.	Ann Genet. 1984;27(2):126-8.	1984
0	RC21-13 (FU)						3499841	Ring chromosome 21 in the mother and 21/21 translocation in the fetus: karyotype: 45,XX,-21,-21,+t(21;21)(p11;q11)	Fryns JP, Kleczkowska A.	Ann Genet. 1987;30(2):109-10.	1987

1	RC21-14 case 1	nb	M			46,XY,r(21)/45,XY,-21	6714262	Three new cases of partial monosomy 21 resulting from one ring 21 chromosome and two unbalanced reciprocal translocations	Philip N, Baeteman MA, Mattei MG, Mattei JF.	Eur J Pediatr. 1984 Apr;142(1):61-4. doi: 10.1007/BF00442594.	1984
1	RC21-15	27 y	M			46,XY,r(21)(p11q22.3)	4075565	Ring chromosome 21 in a phenotypically normal but infertile man	Huret JL, Leonard C, Kanoui V.	Clin Genet. 1985 Dec;28(6):541-5. doi: 10.1111/j.1399-0004.1985.tb00423.x.	1985
1	RC21-16	pn	M	de novo	CtB	AF/SF: 46,XY,r(21)(p13q22)/45,XY,-21/r-var	3160292	Ring chromosome 21: characterization of DNA sequences at sites of breakage and reunion	Kazazian HH Jr, Antonarakis SE, Wong C, Trusko SP, Stetten G, Oliver M, Potter MJ, Gusella JF, Watkins PC.	Ann N Y Acad Sci. 1985;450:33-42. doi: 10.1111/j.1749-6632.1985.tb21481.x.	1985
1	RC21-17	nb	F	de novo		46,XX,r(21)(p11.2q22.3)[0.77]/45,XX,-21[0.23]	3564968	Ring chromosome 21 and SOD activity of blood cells	Aoki T, Yoshimitsu K, Itodagawa M, Okazaki H, Sugimoto T, Kobayashi Y.	Acta Paediatr Scand. 1986 Nov;75(6):1055-8. doi: 10.1111/j.1651-2227.1986.tb10343.x.	1986
1	RC21-18						3733078	Ring chromosome 21 in healthy persons: different consequences in females and in males	Dallapiccola B, De Filippis V, Notarangelo A, Perla G, Zelante L.	Hum Genet. 1986 Jul;73(3):218-20. doi: 10.1007/BF00401230.	1986
1	RC21-19	8 m	M			46,XY,r(21)(p13q22.3)[44]/45,XY,-21[4]/r-var[2]	3815879	Ring 21 chromosome: the mild end of the phenotypic spectrum	Gardner RJ, Monk NA, Clarkson JE, Allen GJ.	Clin Genet. 1986 Dec;30(6):466-70. doi: 10.1111/j.1399-0004.1986.tb01912.x.	1986
0	RC21-20						3621668	Holoprosencephaly associated with ring chromosome 21	Hoovers JM, Jansweijer MC.	Clin Genet. 1987 Sep;32(3):207. doi: 10.1111/j.1399-0004.1987.tb03357.x.	1987
1	RC21-21	13 y	M			46,XY,r(21)c	3474055	Acute lymphoblastic leukemia in a child with constitutional ring chromosome 21	Falchi AM, Orofino MG, Nucaro AL, De Virgiliis S, Cao A.	Cancer Genet Cytogenet. 1987 Aug;27(2):219-24. doi: 10.1016/0165-4608(87)90003-3.	1987
1	RC21-22	15 y	M	mat	> 20 y (mat)	46,XY,r(21), mat: 46,XX,r(21)(p11q22.3)	2887318	Familial transmission of a ring chromosome 21	Hertz JM.	Clin Genet. 1987 Jul;32(1):35-9. doi: 10.1111/j.1399-0004.1987.tb03320.x.	1987
1	RC21-23	3 m	M	de novo		46,XY,r(21)	3568433	A male infant with holoprosencephaly, associated with ring chromosome 21	Aronson DC, Jansweijer MC, Hoovers JM, Barth PG.	Clin Genet. 1987 Jan;31(1):48-52. doi: 10.1111/j.1399-0004.1987.tb02766.x.	1987
1	RC21-24						2960262	Tandem duplication chromosome 21 in the offspring of a ring chromosome 21 carrier	Miller K, Reimer A, Schulze B.	Ann Genet. 1987;30(3):180-2.	1987
1	RC21-25	3.5 y	M	de novo		PB: 45,XY,-21, SF: 46,XY,r(21)[0.80]/45,XY,-21[0.20]	3236369	Apparent monosomy 21 owing to a ring 21 chromosome: parental origin revealed by DNA analysis	Dalgleish R, Duckett DP, Woodhouse M, Shannon RS, Young ID.	J Med Genet. 1988 Dec;25(12):851-4. doi: 10.1136/jmg.25.12.851.	1988
0	RC21-26						2648387	Molecular mechanism in the formation of a human ring chromosome 21	Wong C, Kazazian HH Jr, Stetten G, Earnshaw WC, Van Keuren ML, Antonarakis SE.	Proc Natl Acad Sci U S A. 1989 Mar;86(6):1914-8. doi: 10.1073/pnas.86.6.1914.	1989
1	RC21-27						2741714	Ring chromosome 21	Zergollern L, Muzinić D, Raic Z.	Acta Med Iugosl. 1989;43(2):147-56.	1989
1	RC21-28	6 m	M	mat	> 20 y (mat)	46,XY,r(21)(p13q22.3) mat: 46,XX,r(21)(p13q22.3)	2253944	Maternal transmission of ring chromosome 21	Kennerknecht I, Barbi G, Vogel W.	Hum Genet. 1990 Nov;86(1):99-101. doi: 10.1007/BF00205185.	1990
1	RC21-29	nb	F	mat	> 20 y (mat)	46,XX,r(21)(p11.2q22.3)	2369070	Ring chromosome 21 transmitted from mother to daughter: its stability in a lymphoblastoid cell line	Ikeuchi T, Yamamoto K, Qiao F, Hayakawa K, Migita T, Nishikawa Y.	Ann Genet. 1990;33(1):32-5.	1990
0	RC21-30						1346075	Mechanisms of ring chromosome formation in 11 cases of human ring chromosome 21	McGinniss MJ, Kazazian HH Jr, Stetten G, Petersen MB, Boman H, Engel E, Greenberg F, Hertz JM, Johnson A,	Am J Hum Genet. 1992 Jan;50(1):15-28.	1992
1	RC21-31	6 m	F	mat	> 21 y	IV1: 46,XX,r(21) III3 (mat): 46,XX,r(21), II-4 (gmat):	1308361	Stable ring chromosome 21: molecular and clinical definition of the lesion	Falik-Borenstein TC, Pribyl TM, Pulst SM, Van Dyke DL, Weiss L, Chu ML,	Am J Med Genet. 1992 Jan 1;42(1):22-8. doi:	1992
1	RC21-32	1 y	F			46,XX,der(18)t(18;21)(q23;q21.1),r(21)(p13q21.1)[0.52]/45,XX,der(18),-21[0.48]	8362906	Unbalanced translocation, t(18;21), detected by fluorescence in situ hybridization (FISH) in a child with 18q- syndrome and a ring chromosome 21	McGinniss MJ, Rosenberg C, Stetten G, Schinzel AA, Binkert F, Petersen MB, Kearns WG, Kazazian HH Jr, Pearson	Am J Med Genet. 1993 Jul 1;46(6):647-51. doi: 10.1002/ajmg.1320460609.	1993
1	RC21-33 case 1	1 m	F	de novo		46,XX,r(21)	7966190	"Compensatory" uniparental disomy of chromosome 21 in two cases	Bartsch O, Petersen MB, Stuhlmann I, Mau G, Frantzen M, Schwinger E,	J Med Genet. 1994 Jul;31(7):534-40. doi: 10.1136/jmg.31.7.534.	1994
1	RC21-34						7507604	[Ring chromosome 21 as a cause of developmental disorder. A case report from the practice of child psychiatry]	Melkild A.	Tidsskr Nor Laegeforen. 1994 Jan 10;114(1):36-8.	1994
1	RC21-35	14 y	M			46,XY,r(21)/45,XY,-21	7847798	Lens dislocation and optic nerve hypoplasia in ring chromosome 21 mosaicism	Meire FM, Fryns JP.	Ann Genet. 1994;37(3):150-2.	1994
1	RC21-36	6 m	M	de novo		46,XY,r(21)	8591669	Characterization of a ring chromosome 21 by FISH-technique	Conte RA, Luke S, Verma RS.	Clin Genet. 1995 Oct;48(4):188-91. doi: 10.1111/j.1399-0004.1995.tb04086.x.	1995
1	RC21-37	pn, 16 gwks	F	mat	CP, > 29 y (mat)	twin A: 46,XY, twin B: 46,XX,r(21)(p13q22)[0.77]/45,XX,-21[0.23]; mat: 46,XX,r(21)(p13;q22)	7784385	Prenatal diagnosis of familial ring 21 chromosome	Melnyk AR, Ahmed I, Taylor JC.	Prenat Diagn. 1995 Mar;15(3):269-73. doi: 10.1002/pd.1970150310.	1995
1	RC21-38	3.5 y	M			46,XY,r(21)(p11q22)	9370908	Hypogammaglobulinaemia in a patient with ring chromosome 21	Ohga S, Nakao F, Narazaki O, Fusazaki N, Aoki T, Kamesaki K, Hara T.	Arch Dis Child. 1997 Sep;77(3):252-4. doi: 10.1136/adc.77.3.252.	1997

1	RC21-39 CGM-14	?	M			46,XY,r(21)	10507727	Molecular characterisation of partial chromosome 21 aneuploidies by fluorescent PCR	Valero R, Marfany G, Gil-Benso R, Ibáñez MA, López-Pajares I, Prieto F,	J Med Genet. 1999 Sep;36(9):694-9.	1999
1	RC21-40	29 y	F	mat, familial	> 29 y (mat)	II3/II5: 46XY,r(21)<br>II7: 46,XX,t(21q21q)	11384641	Familial gonadotropin-releasing hormone resistance and hypogonadotropic hypogonadism in a family with multiple affected individuals	Layman LC, McDonough PG, Cohen DP, Maddox M, Tho SP, Reindollar RH.	Fertil Steril. 2001 Jun;75(6):1148-55. doi: 10.1016/s0015-0282(01)01782-4.	2001
1	RC21-41	31 y	F		> 31 y	BM/PB: 46,XX,r(21)c<br>BM: 46,XX,r1[7]/47,idem,+r2[7]/47,idem,+8[9]/47~48,diem,+r2[15]	11165321	Amplification of the AML1(CBFA2) gene on ring chromosomes in a patient with acute myeloid leukemia and a constitutional ring chromosome 21	Streubel B, Valent P, Lechner K, Fonatsch C.	Cancer Genet Cytogenet. 2001 Jan 1;124(1):42-6. doi: 10.1016/s0165-4608(00)00318-6.	2001
1	RC21-42	12 y	M	mat	> 20 y (mat)	46,XY,r(21)[96]/45,XY,-21[4]<br>mat: 46,XX,der(21)	12116206	Ring chromosome 21 in a boy and a derivative chromosome 21 in the mother: implication for ring chromosome formation	Muroya K, Yamamoto K, Fukushima Y, Ogata T.	Am J Med Genet. 2002 Jul 15;110(4):332-7. doi: 10.1002/ajmg.10466.	2002
1	RC21-43	nb	F	de novo		46,XX,r(21)(p11.2q22.3)[0.86]/45,XX,-21[0.14]	14673898	Dystonia in a patient with ring chromosome 21	Hou CE, Schlaggar BL, Racette BA.	Mov Disord. 2003 Dec;18(12):1547-9. doi: 10.1002/mds.10621.	2003
1	RC21-44	nb	F	de novo		AC/PB: 46,XX,1ps,r921)(p11.2q22.3)[48/50]	12838556	Ring 21 chromosome and a satellited 1p in the same patient: novel origin for an ectopic NOR	Ki A, Rauen KA, Black LD, Kostiner DR, Sandberg PL, Pinkel D, Albertson DG,	Am J Med Genet A. 2003 Jul 30;120A(3):365-9. doi:	2003
1	RC21-45	9 m	M	de novo		46,XY,r(21)	15372526	Dilated ascending aorta in a child with ring chromosome 21 syndrome	Rope AF, Hinton RB, Spicer RL, Blough-Pfau R, Saal HM.	Am J Med Genet A. 2004 Oct 1;130A(2):191-5. doi: 10.1002/ajmg.a.30143.	2004
1	RC21-46						15033121	[Chromosome 21 ring (r21) and epilepsy]	Pardal Fernández JM, Jerez García P, Carrascosa Romero MC, Marco Giner J.	An Pediatr (Barc). 2004 Apr;60(4):379-81. doi:	2004
1	RC21-47	2 y	M	de novo		46,XY,r(21)[91]/46,XY,r(21;21)[5]/45,XY,-21[4]	16331572	[Cytogenetic analysis and phenotype location analysis on the karyotype of a ring chromosome	Zhu XY, Zhao R, Ye ZC, Peng YG, Tan YQ.	Zhonghua Yi Xue Yi Chuan Xue Za Zhi. 2005 Dec;22(6):682-3.	2005
1	RC21-48	nb	F	de novo		46,XX,r(21)	16155419	Duplication of the Down syndrome critical region does not predict facial phenotype in a baby with a ring chromosome 21	Crombez EA, Dipple KM, Schimmenti LA, Rao N.	Clin Dysmorphol. 2005 Oct;14(4):183-7. doi: 10.1097/00019605-200510000-00003.	2005
1	RC21-49	14 y	M			46,XY,r(21)	18970914	Videoendoscopic rehabilitation of iatrogenous Stensen-duct-acquired atresia in a patient with ring chromosome 21 syndrome and drooling	Capaccio P, Clemente IA, Marchisio P, Selicorni A, Esposito S, Pignataro L.	J Pediatr Surg. 2008 Nov;43(11):e17-20. doi: 10.1016/j.jpedsurg.2008.06.042.	2008
1	RC21-50	31 y	F	mat	> 31 y	46,XX,r(21)[0.94]/45,XX,-21[0.04]<br>mat: 46,XX,r(21)[0.98]/45,XX,-21[0.02]	18371955	Ring chromosome 21 and reproductive pattern: a familial case and review of the literature	Bertini V, Valetto A, Uccelli A, Tarantino E, Simi P.	Fertil Steril. 2008 Nov;90(5):2004.e1-5. doi: 10.1016/j.fertnstert.2008.01.087. Epub 2008 Apr 18.	2008
1	RC21-51	3 m	M			46,XY,der(21;21)(q10;q10)[141]/46,XY,r(21)(p11q22)[15]	19778489	Rare chromosomal complement of trisomy 21 in a boy conceived by IVF and cryopreservation	Quiroga R, Roselló M, Martinez F, Ferrer-Bolufer I, Monfort S, Oltra S, Hernandez MC, Orellana C.	Reprod Biomed Online. 2009 Sep;19(3):415-7. doi: 10.1016/s1472-6483(10)60177-0.	2009
1	RC21-52	> 20 y	M		> 20 y	46,XY,r(21)[95]/45,XY,-21[3]/46,XY[2]	19135661	Sperm chromosome analysis of an infertile patient with a 95% mosaic r(21) karyotype and normal phenotype	Hammoud I, Gomes DM, Bergere M, Wainer R, Selva J, Vialard F.	Fertil Steril. 2009 Mar;91(3):930.e13-5. doi: 10.1016/j.fertnstert.2008.12.005. Epub 2009 Jan 10.	2009
1	RC21-53	7 m	M	de novo		46,XY,r(21)	19864007	ITGB2 mutation combined with deleted ring 21 chromosome in a child with leukocyte adhesion deficiency	Fiorini M, Piovani G, Schumacher RF, Magri C, Bertini V, Mazzolari E, Notarangelo L, Notarangelo LD, Barlati	J Allergy Clin Immunol. 2009 Dec;124(6):1356-8. doi: 10.1016/j.jaci.2009.07.058.	2009
1	RC21-54	3 y	F		> 30 y	46,XX,r(21)(p11q22.3)	25385124	An adult female patient with ring chromosome 21: behavioural phenotype and results of high-resolution molecular characterisation	Verhoeven WM, Van Bon B, Egger JI, Hoischen A, Doelman JC.	Acta Neuropsychiatr. 2010 Aug;22(4):188-94. doi: 10.1111/j.1601-5215.2010.00455.x.	2010
1	RC21-55	pn, 21 gwks	M	pat	CtB	46,XY,r(21)(p11.2q22)[34]/45,XY,-21[4]/46,XY[14] pat: 46,XY,r(21)[1]/46,XY[99]	20509162	A fetus with ring chromosome 21 characterized by aCGH shows no clinical findings after birth	Papoulidis I, Manolakos E, Siomou E, Kefalas K, Thomaidis L, Liehr T, Vetro A, Athanasiadis A, Zuffardi O, Petersen	Prenat Diagn. 2010 Jun;30(6):586-8. doi: 10.1002/pd.2524.	2010
1	RC21-56	pn, 15 gwks	F	mat	CtB, > 40 y (mat)	46,XX,r(21)(p11.2q22.3<br>mat: 46,XX,r(21)(p11.2q22.3)[15]/46,XX[35]	23074672	Inheritance of a Ring Chromosome 21 in a Couple Undergoing In Vitro Fertilization (IVF): A Case Report	Mazzaschi RL, Love DR, Hayes I, George A.	Case Rep Genet. 2011;2011:158086. doi: 10.1155/2011/158086. Epub 2011 Jul 31.	2011
1	RC21-57	3 y	M			46,XY,r(21)(p13q22.3)[17]/45,XY,-21[3]	21595005	Ring 21 chromosome presenting with epilepsy and intellectual disability: clinical report and review of the literature	Specchio N, Carotenuto A, Trivisano M, Cappelletti S, Digilio C, Capolino R, Di Capua M, Fusco L, Vigevano F.	Am J Med Genet A. 2011 Apr;155A(4):911-4. doi: 10.1002/ajmg.a.33899. Epub 2011 Mar 15.	2011
1	RC21-58						23178995	Agminated lentiginosis in a patient with ring chromosome 21	23178995	Eur J Dermatol. 2012 Nov-Dec;22(6):801-3. doi: 10.1684/ejd.2012.1866.	2012
1	RC21-59	pn, 22 gwks	M	de novo	top	46,XY,r(21)[88]/45,XY,-21[9]/46,XY,idic r(21)[3]	22482972	Mosaic ring chromosome 21, monosomy 21, and isodicentric ring chromosome 21: prenatal diagnosis, molecular cytogenetic characterization, and association with 2-Mb deletion of 21q21.1-q21.2 and 5-Mb deletion of 21q22.3	Chen CP, Lin YH, Chou SY, Su YN, Chern SR, Chen YT, Town DD, Chen WL, Wang W.	Taiwan J Obstet Gynecol. 2012 Mar;51(1):71-6. doi: 10.1016/j.tjog.2012.01.014.	2012

1	RC21-60 case 1	1 y	F	de novo		46,XX,r(21)[97]/45,XX,-21[3]	22398511	Unique genomic structure and distinct mitotic behavior of ring chromosome 21 in two unrelated cases	Zhang HZ, Xu F, Seashore M, Li P.	Cytogenet Genome Res. 2012;136(3):180-7. doi: 10.1159/000336978. Epub 2012 Mar 7.	2012
1	RC21-60 case 2	1 y	M			46,XY,r(21)[87]/45,XY,-21[13]	22398511				
1	RC21-61	1 y	M	de novo		46,XY,r(21)	22209335	Ring chromosome 21 in the differential diagnosis of waddling gait	Arslan M, Yiş U, Vurucu S, Tunca Y, Unay B, Akin R.	Brain Dev. 2012 Oct;34(9):792-5. doi: 10.1016/j.braindev.2011.12.003. Epub 2011 Dec 29.	2012
1	RC21-62	3 y	M	de novo		47,XY,+21[0.28]/47,XY,+r(21)[0.04]/46,XY[0.68]	23000017	Mosaicism for trisomy 21 and ring (21) in a male born to normal parents: a case report	Samarth RM, Gandhi P, Pandey H, Maudar KK.	Gene. 2012 Dec 10;511(1):109-12. doi: 10.1016/j.gene.2012.09.035. Epub 2012 Sep 20.	2012
1	RC21-[1]	2 m	M	de novo	d. 3 m	46,XY,r(21)	24052730	Ring autosomes: some unexpected findings	Caba L, Rusu C, Plăiaşu 5th, Gug G, Grămescu M, Bujoran C, Ochiană D, Voloşciuc M, Popescu R, Braha E,	Balkan J Med Genet. 2012 Dec;15(2):35-46. doi: 10.2478/bjmg-2013-0005.	2012
0	RC21-63						23545316	Ring chromosome 21 presenting with sacrococcygeal teratoma: prenatal diagnosis, molecular cytogenetic characterization and	Chen CP, Cheng PJ, Chang SD, Lee YX, Shih JC, Chern SR, Wu PS, Su JW, Chen YT, Hsieh AH, Chen TH, Chen LF, Wang	Gene. 2013 Jun 10;522(1):111-6. doi: 10.1016/j.gene.2013.03.064. Epub 2013 Mar 29	2013
1	RC21-64	nb	F		d. 4.5 m	46,XX,r(21)(p11q22)[42]/45,XX,-21[8]	24649383	Monosomy 21 seen in live born is unlikely to represent true monosomy 21: a case report and review of the literature	Burgess T, Downie L, Pertile MD, Francis D, Glass M, Nouri S, Pszczola R.	Case Rep Genet. 2014;2014:965401. doi: 10.1155/2014/965401. Epub 2014 Feb 4.	2014
1	RC21-65	30 y	F		> 30 y	46,XX,r(21)[166]/46,XX,der(21)[60]/45,XX,-21[20]	25449084	[Analysis of a infertile female with ring 21 chromosome using combined techniques]	Wang H, Wang Y, Wu L, Xie L.	Zhonghua Yi Xue Yi Chuan Xue Za Zhi. 2014 Dec;31(6):761-4. doi:	2014
1	RC21-66	33 y	M			46,XY,r(21)(p13q22.3)[44]/46,XY,dic r(21)[4]/45,XY,-21[52], FISH: r(21)[0.75]/-21[0.25]	24471893	Ring chromosome 21 and monosomy 21 mosaicism in a patient with azoospermia	Cetin Z, Altiok-Clark O, Sevuk M, Berker Karauzum S.	Andrologia. 2015 Feb;47(1):112-5. doi: 10.1111/and.12232. Epub 2014 Jan 28.	2015
1	RC21-67	4 y	F			PB: 46,XX,r(21)(p11.2q22.3)c, BM: 46,XX,r(21)c[11]/47,idem,+r(21)[3]	26947932	RUNX1 amplification in AML with myelodysplasia-related changes and ring 21 chromosomes	Burillo-Sanz S, Vargas MT, Morales-Camacho RM, Caballero-Velázquez T, Sánchez J, García-Lozano JR, Pérez de	Hematol Oncol. 2017 Dec;35(4):894-899. doi: 10.1002/hon.2287. Epub 2016 Mar 7.	2017
1	RC21-68	5 y	F			46,XX,r(21)(q11.2q22.3)/45,XX,-21, FISH r(21)[0.35]/-21[0.65]	29656336	Combined Immunodeficiency with Ring Chromosome 21	Norman M, Wainstein B, Anazodo A, Turner A, Ma C, Payne K, Tangye SG,	J Clin Immunol. 2018 Apr;38(3):251-256. doi: 10.1007/s10875-018-0493-	2018
1	RC21-69	2 y	M			46,XY,r(21)	30922549	[Alopecia, deformed ear and mental retardation associated with terminal 21q deletion]	Lafabregue E, Chaby G, Vabres P, Carmi E.	Ann Dermatol Venereol. 2019 Sep;146(8-9):563-570. doi:	2019
1	RC21-70	18 y	F	de novo	> 18 y	46,XX,der(21)t(21;21)[92]/45,XX,der(21),-21	31195399	Unexpected Coexistence of a Derivative t(21;21) and Complementary Mosaic r(21) in a Female with Multiple Miscarriages	Onur Cura D, Bora E, Ozkalayci H, Kirbiyik O, Kutbay YB, Ercal D, Cankaya T.	Cytogenet Genome Res. 2019;158(2):83-87. doi: 10.1159/000500986. Epub 2019 Jun 14.	2019
1	RC21-71	12 y	F			46,XX,r(21)[0.60]/45,XX,-21[0.40]	30800047	Thrombocytopenia and Predisposition to Acute Myeloid Leukemia due to Mosaic Ring 21 with Loss of RUNX1: Cytogenetic and Molecular	Vormittag-Nocito E, Ni H, Schmidt ML, Lindgren V.	Mol Syndromol. 2019 Jan;9(6):306-311. doi: 10.1159/000494645. Epub 2018 Nov 9.	2019
1	RC21-72	10.5 y	M			46,XY,r(21)	32663882	A Revisited Diagnosis of Collagen VI Related Muscular Dystrophy in a Patient with a Novel COL6A2 Variant and 21q22.3 Deletion	Simsek-Kiper PO, Oguz S, Ergen FB, Utine GE, Alikasifoglu M, Haliloglu G.	Neuropediatrics. 2020 Dec;51(6):445-449. doi: 10.1055/s-0040-1714125. Epub 2020 Jul 14.	2020
1	RC21-73	pn, 18 gwks	F	de novo	top	46,XX,r(21)(p11.2q22.3)	33494993	Prenatal diagnosis and molecular cytogenetic characterization of a pure ring chromosome 21 with a 4.657-Mb 21q22.3 deletion	Chen CP, Wang LK, Chern SR, Wu PS, Chen SW, Wu FT, Chen YY, Town DD, Wang W.	Taiwan J Obstet Gynecol. 2021 Jan;60(1):157-160. doi: 10.1016/j.tjog.2020.11.024.	2021
**72**	**74**										
1	RC22-1	5.5 y	F	de novo		Twin: 46,XX,r(22)	4697858	Monozygotic twins with ring chromosome 22	Lindenbaum RH, Bobrow M, Barber L.	J Med Genet. 1973 Mar;10(1):85-9. doi: 10.1136/jmg.10.1.85.	1973
1	RC22-2	5 y	F		> 38 y	46,XX,r(22)	1214291	A profoundly mentally handicapped woman with a ring chromosome 22	Veall RM, Rundle AT, Chitham RC, Saldana-Garcia P.	J Ment Defic Res. 1975 Sep-Dec;19(3-4):225-43. doi: 10.1111/j.1365-2788.1975.tb01275.x.	1975
1	RC22-3	18 y	F		> 18 y	46,XX,r(22)	940150	A note on a patient with a ring-22 chromosome identified by banding	Stewart A, Richards BW.	J Ment Defic Res. 1976 Jun;20(2):95-8. doi: 10.1111/j.1365-2788.1976.tb00933.x.	1976
1	RC22-4 pt1	3 y	F	de novo		PB: 46,XX,r(22)[100/100/47, 1968/71/76], SF: r(22)[0.91]	912941	Phenotypic correlations in patients with ring chromosome 22	Hunter AG, Ray M, Wang HS, Thompson DR.	Clin Genet. 1977 Oct;12(4):239-49. doi: 10.1111/j.1399-0004.1977.tb00933.x.	1977
1	RC22-4 pt2	36 y	F		> 41 y	PB/SF: 46,XX,r(22)[15/15; 16/30]	912941				
1	RC22-5 pt 1	4 y	F	de novo		46,XX,r(22)	519902	Phenotypic variation in two patients with a ring chromosome 22	Funderburk SJ, Sparkes RS, Klisak I.	Clin Genet. 1979 Nov;16(5):305-10. doi: 10.1111/j.1399-0004.1979.tb01007.x.	1979
1	RC22-5 pt 2	1 m	F			46,XX,r(22)	519902				

1	RC22-6	2 m	M	mat	> 21 y (mat)	46,XY,r(22)/45,XY,-22 mat: 46,XX,r(22)/45,XY,t(15;22)(p11;q11)/46,XX	437787	Ring chromosome 22 in a mentally retarded child and mosaic 45,XX,-15,-22,+t(15;22)(p11;q11)/46,XX,r(22)/46,XX karyotype in the mother	Fryns JP, Van den Berghe H.	Hum Genet. 1979 Mar 12;47(2):213-6. doi: 10.1007/BF00273205.	1979
0	RC22-7						7150788	[Ring chromosome 22: r(22)]	Teyssier M, Moreau N.	Bull Assoc Anat (Nancy). 1982	1982
1	RC22-8	28 y	M		> 28 y	46,XY,r(22)	3966887	Methylphenidate therapy for aggression in a man with ring 22 chromosome. Report and literature review	Reeve A, Shulman SA, Zimmerman AW, Cassidy SB.	Arch Neurol. 1985 Jan;42(1):69-72. doi: 10.1001/archneur.1985.04060010075019.	1985
1	RC22-9	1.5 y	M		d. 27 y	46,XY,r(22)(p12q13.3)	3712397	Multifocal meningiomas in a patient with a constitutional ring chromosome 22	Arinami T, Kondo I, Hamaguchi H, Nakajima S.	J Med Genet. 1986 Apr;23(2):178-80. doi: 10.1136/jmg.23.2.178.	1986
1	RC22-10	9 y	M	de novo		46,XY,r(22)(p11q13)[24]/46,XY,dic r[3]/46,XY,del(22)(p12)(q13)[40]	2872982	Deleted ring chromosome 22 in a mentally retarded boy	Gustavson KH, Arancibia W, Eriksson U, Svennerholm L.	Clin Genet. 1986 Apr;29(4):337-41. doi: 10.1111/j.1399-0004.1986.tb01264.x.	1986
1	RC22-11 pt PM	11 y	M			46,XY,r(21)	3652494	Chromosome studies in IgA-deficient patients	Taalman RD, Weemaes CM, Hustinx TW, Scheres JM, Clement JM, Stoelinga	Clin Genet. 1987 Aug;32(2):81-7. doi: 10.1111/j.1399-	1987
1	RC22-12						3627062	[Ring chromosome 22. Description of a clinical case]	Pizzi E, de la Pierre L, Gargantini G, Andreoli A, Rogari P, Bonora G,	Minerva Pediatr. 1987 Jun 30;39(11-12):525-9.	1987
1	RC22-13						3619316	[Ring chromosome 22 associated with right polycystic kidney and a left junction syndrome]	Lassen C, Mettey R, Berthier M, Bonneau D, Gremmo G, Hoppeler A.	Ann Pediatr (Paris). 1987 Jun;34(6):451-2.	1987
1	RC22-14	5 y	M		> 40 y	PB/SF: 46,XY,r(22)	3100017	Neurofibromatosis in a man with a ring 22: in situ hybridization studies	Duncan AM, Partington MW, Soudek D.	Cancer Genet Cytogenet. 1987 Mar;25(1):169-74. doi: 10.1016/0165-4608(87)90173-7.	1987
1	RC22-15	3 y	F	de novo		46,XX,r(22)(p11.2q13.3)	3180509	Ring chromosome 22 46,XX,r(22)(p11.2----q13.3) presenting with leukemoid reaction	Watanabe H, Yamanaka T.	Clin Genet. 1988 Sep;34(3):206-7. doi: 10.1111/j.1399-0004.1988.tb02865.x.	1988
1	RC22-16	2 y	F	de novo		46,XX,r(22)(p13q13.3)	2455825	Determination of the breakpoints and the parental origin of a ring 22 chromosome: an analysis by high-resolution banding technique, quinacrine and silver stainings	Naritomi K, Hirayama K.	Jinrui Idengaku Zasshi. 1988 Mar;33(1):67-73. doi: 10.1007/BF01891242.	1988
1	RC22-17	nb	M			46,XY,r(22)(p13q13.3)	2260575	Ring chromosome 22 karyotype in a patient with Opitz (BBBG) syndrome	Christodoulou J, Bankier A, Loughnan P.	Am J Med Genet. 1990 Nov;37(3):422-4. doi: 10.1002/ajmg.1320370324.	1990
1	RC22-18	3 y	F			46,XX,r(20)	1721941	Ring chromosome 22: a case report	Severien C, Felix S, Bartholomé K.	Klin Padiatr. 1991 Nov-Dec;203(6):467-9. doi: 10.1055/s-2007-1025476.	1991
1	RC22-19						1772175	[Ring chromosome 22. Description of a new case (1)]	Gil Benso R, López Ginés C, Gregori Romero M, Galán Sánchez F, Pellín	An Esp Pediatr. 1991 Jul;35(1):62-4.	1991
1	RC22-20	3 y	M	pat	> 25 y	PB/SF: mos 46,XX,r(22)	1424240	Ring chromosome 22 and neurofibromatosis	Tommerup N, Warburg M, Gieselmann V, Hansen BR, Koch J, Petersen GB.	Clin Genet. 1992 Oct;42(4):171-7. doi: 10.1111/j.1399-	1992
1	RC22-21	15 y	M			46,XY,r(22)(p11q13)	8213904	Multiple meningiomas in a patient with constitutional ring chromosome 22	Petrella R, Levine S, Wilmot PL, Ashar KD, Casamassima AC, Shapiro LR.	Am J Med Genet. 1993 Aug 15;47(2):184-6. doi:	1993
1	RC22-21.5	3 y	M	de novo		46,XY,r(22)	7815438	Asymmetry and skin pigmentary anomalies in chromosome mosaicism	Woods CG, Bankier A, Curry J., et al	J Med Genet 1994, 31:694-701	1994
1	RC22-22	1 y	F			46,XX,r(22)	8558556	Metachromatic leucodystrophy (MLD) in a patient with a constitutional ring chromosome 22	Coulter-Mackie MB, Rip J, Ludman MD, Beis J, Cole DE.	J Med Genet. 1995 Oct;32(10):787-91. doi: 10.1136/jmg.32.10.787.	1995
1	RC22-23	21 y	M		> 21 y	46,XY,r(22)	8930062	Ring chromosome 22 and mood disorders	Sovner R, Stone A, Fox C.	J Intellect Disabil Res. 1996 Feb;40 (Pt 1):82-6. doi: 10.1111/j.1365-2788.1996.tb00607.x.	1996
1	RC22-24	4 y	F		> 38 y	46,XX,r(22)/45,XX,-22	9225971	Mutational analysis and expression studies of the neurofibromatosis type 2 (NF2) gene in a patient with a ring chromosome 22 and NF2	Kehrer-Sawatzki H, Udart M, Krone W, Baden R, Fahsold R, Thomas G, Schmucker B, Assum G.	Hum Genet. 1997 Jul;100(1):67-74. doi: 10.1007/s004390050467.	1997
1	RC22-25	4 y	F			46,XX,r(22)	9217983	March 1997--4 year old girl with ring chromosome 22 and brain tumor	Rubio A.	Brain Pathol. 1997 Jul;7(3):1027-8. doi: 10.1111/j.1750-3639.1997.tb00902.x.	1997
1	RC22-26						10487083	Ring chromosome 22 resulting in partial monosomy in a mentally retarded boy	Gibbons B, Tan SY, Tam PY.	Singapore Med J. 1999 Apr;40(4):273-5.	1999
1	RC22-27	nb	F	de novo		46,XX,r(22)(p12q13)[46]/46,XX,dic r[4]	10204853	Ring 22 duplication/deletion mosaicism: clinical, cytogenetic, and molecular characterisation	Frizzley JK, Stephan MJ, Lamb AN, Jonas PP, Hinson RM, Moffitt DR,	J Med Genet. 1999 Mar;36(3):237-41.	1999
1	RC22-28	6 m	M			46,XY,r(22)	10706359	Ring chromosome 22 and autism: report and	MacLean JE, Teshima IE, Szatmari P,	Am J Med Genet. 2000 Feb	2000
1	RC22-29	7 y	M	de novo		46,XY,r(22)	11950869	Molecular characterisation of a ring chromosome 22 in a patient with severe language delay: a contribution to the refinement of the subtelomeric 22q deletion syndrome	De Mas P, Chassaing N, Chaix Y, Vincent MC, Julia S, Bourrouillou G, Calvas P, Bieth E.	J Med Genet. 2002 Apr;39(4):e17. doi: 10.1136/jmg.39.4.e17.	2002

1	RC22-30 subj1	5.5 y	M	de novo		46,XY,r(22)(p11.2q13)	12752574	Five new subjects with ring chromosome 22	Ishmael HA, Cataldi D, Begleiter ML, Pasztor LM, Dasouki MJ, Butler MG.	Clin Genet. 2003 May;63(5):410-4. doi: 10.1034/j.1399-0004.2003.00064.x.	2003
1	RC22-30 subj2	35 y	M		> 35 y	46,XY,r(22)(p11.2q13)	12752574				
1	RC22-30 subj3	7 y	F			46,XX,r(22)	12752574				
1	RC22-30 subj4	8 y	M			46,XY,r(22)	12752574				
1	RC22-30 subj5	16 y	M			46,XY,r(22)(p12q13)	12752574				
1	RC22-31	pn	F	de novo	top	46,XX,r(22)(p13q13.31)	12533811	Prenatal diagnosis of mosaic ring chromosome 22 associated with cardiovascular abnormalities and intrauterine growth restriction	Chen CP, Chern SR, Chang TY, Lee CC, Chen LF, Tzen CY, Wang W, Lin CJ, Yang BP, Yang LS.	Prenat Diagn. 2003 Jan;23(1):40-3. doi: 10.1002/pd.517.	2003
1	RC22-32	3 y	M	de novo		46,XY,r(22)	15372517	Characterization of the phenotype and definition of the deletion in a new patient with ring chromosome 22	Battini R, Battaglia A, Bertini V, Cioni G, Parrini B, Rapalini E, Simi P, Tinelli F, Valetto A.	Am J Med Genet A. 2004 Oct 1;130A(2):196-9. doi: 10.1002/ajmg.a.30276.	2004
1	RC22-33	pn, 16 gwks	M	de novo	top	46,XX,r(22)	15305353	A case of ring chromosome 22 with deletion of the 22q13.3 region associated with agenesis of the corpus callosum, fornix and septum pellucidum	Delcán J, Orera M, Linares R, Saavedra D, Palomar A.	Prenat Diagn. 2004 Aug;24(8):635-7. doi: 10.1002/pd.955.	2004
0	RC22-34						16059935	Molecular and phenotypic characterization of ring chromosome 22	Jeffries AR, Curran S, Elmslie F, Sharma A, Wenger S, Hummel M, Powell J.	Am J Med Genet A. 2005 Aug 30;137(2):139-47. doi:	2005
1	RC22-35	pn, 12 gwks	F	mat	top, 22 gwks	46,XX,der(22)r(22)(p11.2q11.2)inv(22)(q11.2 q11.3) mat: 46,XX,inv(22)(q11.2q13.3)	17099929	Prenatal diagnosis of an unexpected interstitial 22q11.2 deletion causing truncus arteriosus and thymic hypoplasia in a ring 22 chromosome derived from a maternally inherited paracentric	McClarren J, Donnenfeld AE, Ravnan JB.	Prenat Diagn. 2006 Dec;26(13):1212-5. doi: 10.1002/pd.1590.	2006
0	RC22-36						18664089	A case with a ring chromosome 22	Koç A, Karaer K, Ergün MA, Yirmibeş-Karaoğuz M, Kan D, Cansu A, Perçin F.	Turk J Pediatr. 2008 Mar-Apr;50(2):193-6.	2008
1	RC22-37	pn	F	de novo	top	46,XX,r(22)(p11q13.2)[63]/45,XX,-22[3]/46,XX,idic r[2]	20149051	Prenatal diagnosis of mosaic ring 22 duplication/deletion with terminal 22q13 deletion due to abnormal first trimester screening and choroid plexus cyst detected on ultrasound	Koç A, Arisoy O, Pala E, Erdem M, Kaymak AO, Erkal O, Karaoğuz MY.	J Obstet Gynaecol Res. 2009 Oct;35(5):978-82. doi: 10.1111/j.1447-0756.2009.01040.x.	2009
1	RC22-38	2 y	F			46,XX,r(22)	19772601	Pathogenesis of vestibular schwannoma in ring chromosome 22	Denayer E, Brems H, de Cock P, Evans GD, Van Calenbergh F, Bowers N, Sciot R, Debiec-Rychter M, Vermeesch JV,	BMC Med Genet. 2009 Sep 22;10:97. doi: 10.1186/1471-2350-10-97.	2009
0	RC22-39						19166580	Meiotic and mitotic behaviour of a ring/deleted chromosome 22 in human embryos determined by preimplantation genetic diagnosis for a maternal carrier	Mantzouratou A, Mania A, Apergi M, Laver S, Serhal P, Delhanty J.	Mol Cytogenet. 2009 Jan 23;2:3. doi: 10.1186/1755-8166-2-3.	2009
1	RC22-40	31 y	M		> 31 y	46,XY,r(22)[93]/45,XY,-22[7]	20709628	Azoospermia in a man with a constitutional ring 22 chromosome	Zuccarello D, Dallapiccola B, Novelli A, Foresta C.	Eur J Med Genet. 2010 Nov-Dec;53(6):389-91. doi:	2010
1	RC22-41	3 y	F	de novo		46,XX,r(22)(p11.2q13)	21031059	Phenotypic correlations in a patient with ring chromosome 22	Demirhan O, Tunç E.	Indian J Hum Genet. 2010 May;16(2):97-9. doi: 10.4103/0971-6866.69372.	2010
1	RC22-42 pt11	9 y	M			46,XY,r(22)	20186804	22q13.3 deletion syndrome: clinical and molecular analysis using array CGH	Dhar SU, del Gaudio D, German JR, Peters SU, Ou Z, Bader PI, Berg JS, Blazo M, Brown CW, Graham BH, Grebe TA, Lalani S, Irons M, Sparagana	Am J Med Genet A. 2010 Mar;152A(3):573-81. doi: 10.1002/ajmg.a.33253.	2010
1	RC22-42 pt12	5 y	M			46,XY,r(22)	20186804				
1	RC22-43	1.5 y	F	mat nl, pat nd		46,XX,r(22)(p11.2q12.3)	19940762	Progressive edema leading to pleural effusions in a female with a ring chromosome 22 leading to a 22q13 deletion	McGaughran J, Hadwen T, Clark R.	Clin Dysmorphol. 2010 Jan;19(1):28-9. doi: 10.1097/MCD.0b013e3283301f58.	2010
1	RC22-44	28 y	M		> 28 y	46,XY,r(22)(p12q13.3)	21843971	Azoospermia and paternal autosomal ring chromosomes: case report and literature review	Rajesh H, Freckmann ML, Chapman M.	Reprod Biomed Online. 2011 Oct;23(4):466-70. doi:	2011
1	RC22-45	pn, 22 gwks	F	de novo	top	46,XX,r(22)(p11q13.2)[63]/45,XX,-22[3]/46,XX,idic r[2]	21271667	Prenatal diagnosis and molecular characterization of two constitutional rings derived from one chromosome 22	Gadji M, Krabchi K, Langis P, Aboura A, Périgny M, Côté S, Ferland M, Drouin R.	Am J Med Genet A. 2011 Feb;155A(2):430-3. doi: 10.1002/ajmg.a.33654.	2011
1	RC22-46	5 y	M	de novo		46,XY,r(22)	24052724	Ring chromosome 22: a review of the literature and first report from India	Mahajan S, Kaur A, Singh J.	Balkan J Med Genet. 2012 Jun;15(1):55-9. doi: 10.2478/v10034-012-0009-8.	2012
1	RC22-47	5 y	M	de novo		46,XY,r(22)(p11q13)	21175598	Ring chromosome 22 and neurofibromatosis type II: proof of two-hit model for the loss of the NF2 gene in the development of meningioma	Zirn B, Arning L, Bartels I, Shoukier M, Hoffjan S, Neubauer B, Hahn A.	Clin Genet. 2012 Jan;81(1):82-7. doi: 10.1111/j.1399-0004.2010.01598.x. Epub 2010 Dec 22.	2012
1	RC22-48	29 y	M		> 29 y	46,XY,r(22)(p11q25)	23405794	[Ring 22 chromosome syndrome induced azoospermia: a case report and literature review]	Sha YW, Ding L, Song YQ, Ge YS, Zeng H, Li P.	Zhonghua Nan Ke Xue. 2012 Dec;18(12):1111-4.	2012
1	RC22-49						23927807	[Ring chromosome 22 in a case]	Pan L, Sun YL, Xu CM.	Zhonghua Er Ke Za Zhi. 2013 Apr;51(4):308-9.	2013

1	RC22-50 pt1	5 y	M	de novo		46,XY,r(22)(p11.2q13.3)	23635516	Molecular and phenotypic characterization of ring chromosome 22 in two unrelated patients	Hannachi H, Mougou S, Benabdallah I, Soayh N, Kahloul N, Gaddour N, Le Lorc'h M, Sanlaville D, El Ghezal H,	Cytogenet Genome Res. 2013;140(1):1-11. doi: 10.1159/000350785. Epub 2013 Apr	2013
1	RC22-50 pt2	5 y	F	de novo		46,XX,r(22)(p11.2q13.3)	23635516				
1	RC22-51	pn, 18 gwks	M		top	AF: 46,XY,r(22)(p13q13)	25286802	Prenatal diagnosis of complete ring chromosome 22 without phenotypical abnormalities	Liou B, Su CF, Luo CH, Tsai HJ.	Taiwan J Obstet Gynecol. 2014 Sep;53(3):406-8. doi: 10.1016/j.tjog.2013.06.016.	2014
1	RC22-52	4 m	M			46,XY,r(22)(p13q13.3)	25114695	Atypical teratoid rhabdoid brain tumor in an infant with ring chromosome 22	Cho EH, Park JB, Kim JK.	Korean J Pediatr. 2014 Jul;57(7):333-6. doi: 10.3345/kjp.2014.57.7.333. Epub 2014 Jul 23.	2014
1	RC22-53 pt1	6 y	F			46,XX,r(22)(p13q13.33)	24700634	Clinical, cytogenetic, and molecular characterization of six patients with ring chromosomes 22, including one with concomitant 22q11.2 deletion	Guilherme RS, Soares KC, Simioni M, Vieira TP, Gil-da-Silva-Lopes VL, Kim CA, Brunoni D, Spinner NB, Conlin LK, Christofolini DM, Kulikowski LD,	Am J Med Genet A. 2014 Jul;164A(7):1659-65. doi: 10.1002/ajmg.a.36512. Epub 2014 Apr 3.	2014
1	RC22-53 pt2	6 y (child)	M		> 24 y	46,XY,r(22)(p13q13.2)	24700634				
1	RC22-53 pt3	1 y	F			46,XX,r(22)	24700634				
1	RC22-53 pt4	7 y	F			46,XX,r(22)	24700634				
1	RC22-53 pt5	2 y	M			46,XY,r(22)	24700634				
1	RC22-53 pt6	21 y	F		> 21 y	46,XX,r(22)[0.88]/46,XX[0.12]	24700634				
1	RC22-54 case 1	3 y	M			46,XY,r(22)(p11q13)[60]	27455005	[Genetic diagnosis and analysis for two cases of ring chromosome 22]	Peng Y, Tang G, Zhang R, Zhang Y, Xia Y, Ma R, Guo R, Wu L.	Zhonghua Yi Xue Yi Chuan Xue Za Zhi. 2016 Aug;33(4):494-7. doi:	2016
1	RC22-54 case 2	2 y	M			46,XY,r(22)(q13.2q13)[84]/45,XY,-22[6]	27455005				
1	RC22-55	10 m	F			46,XX,r(22)(q13.1q13.3)[19]/46,XX,dic r[1]	27734605	Description of a new oncogenic mechanism for atypical teratoid rhabdoid tumors in patients with ring chromosome 22	Byers HM, Adam MP, LaCroix A, Leary SE, Cole B, Dobyns WB, Mefford HC.	Am J Med Genet A. 2017 Jan;173(1):245-249. doi: 10.1002/ajmg.a.37993. Epub 2016 Oct 12.	2017
1	RC22-56	4 y	F			46,XX,r(21)	29736186	Compound phenotype in a girl with r(22), concomitant microdeletion 22q13.32-q13.33 and mosaic monosomy 22	Kashevarova AA, Belyaeva EO, Nikonov AM, Plotnikova OV, Skryabin NA, Nikitina TV, Vasilyev SA, Yakovleva YS, Babushkina NP, Tolmacheva EN,	Mol Cytogenet. 2018 Apr 27;11:26. doi: 10.1186/s13039-018-0375-3. eCollection 2018.	2018
1	RC22-57	3 y	F			46,XX,r(22)(p11.2q13)	29378768	Chromothripsis and ring chromosome 22: a paradigm of genomic complexity in the Phelan-McDermid syndrome (22q13 deletion syndrome)	Kurtas N, Arrigoni F, Errichiello E, Zucca C, Maghini C, D'Angelo MG, Beri S, Giorda R, Bertuzzo S, Delledonne M,	J Med Genet. 2018 Apr;55(4):269-277. doi: 10.1136/jmedgenet-2017-105125. Epub 2018 Jan 29.	2018
1	RC22-58					r(22)(q13.3)	30647996	MLPA is a practical and complementary alternative to CMA for diagnostic testing in patients with autism spectrum disorders and identifying new candidate CNVs associated with autism	Capkova P, Srovnal J, Capkova Z, Staffova K, Becvarova V, Trkova M, Adamova K, Santava A, Curtisova V, Hajduch M, Prochazka M.	PeerJ. 2019 Jan 9;6:e6183. doi: 10.7717/peerj.6183. eCollection 2019.	2019
1	RC22-59	18 y	F		> 18 y	46,XX,r(22)	33550291	Bilateral Vestibular Schwannomas in a Patient with Ring Chromosome 22: Case Report and Review of the Literature	Nussbaum PE, Patel PD, Nussbaum LA, Hilton CW, Nussbaum ES.	Pediatr Neurosurg. 2021 Feb 5:1-5. doi: 10.1159/000513112. Online ahead of print.	2021
1	RC22-60	2.5 y	M	de novo		46,XY,r(22)(p11.2q13.32)	34777208	Case Report: The Emerging Role of Ring Chromosome 22 in Phelan-McDermid Syndrome With Atypical Teratoid/Rhabdoid Tumor: The First Child Treated With Growth Hormone	Crocco M, Panciroli M, Milanaccio C	Front Neurol. 2021 Oct 29;12:741062	2021
**71**	**61**										
		pn: prenatal; cp/ctb: continous pregnancy/to birth; nd: not detected; d., deceased;									
1	RCX-[1]							Self-perpetuating ring chromosome in a patient with gonadal dysgenesis	Lindstern J, Rillinger KG	Lancet 1962 279(7229):593-594	
1	RCX-1						14147860	A RING-X-CHROMOSOME IN PART OF THE SOMATIC CELLS OF A PATIENT WITH SOME CHARACTERISTICS OF THE TURNER SYNDROME	HUSTINX TW, STOELINGA GB.	Genetica. 1964;35:1-14. doi: 10.1007/BF01804870.	1964
1	RCX-2						14172991	H3-THYMIDINE UPTAKE BY A RING X CHROMOSOME IN A HUMAN FEMALE	ROWLEY J, MULDAL S, LINDSTEN J, GILBERT CW.	Proc Natl Acad Sci U S A. 1964 May;51(5):779-86. doi: 10.1073/pnas.51.5.779.	1964
1	RCX-3						14235684	XG-A INVESTIGATIONS OF THE FAMILY OF A CHILD WITH A RING X CHROMOSOME	NIJENHUIS LE, GEMSER-RUNIA J.	Nature. 1964 Nov 21;204:792-3. doi: 10.1038/204792a0.	1964
1	RCX-4						4165104	A ring X chromosome in dwarfism	Bain AD, Gauld IK, Farquhar JW.	Lancet. 1965 Apr 10;1(7389):820. doi: 10.1016/s0140-6736(65)92992-2.	1965
1	RCX-5						5885928	Xg-a investigations of the family of a child with a ring X chromosome	Sanger R, Race RR.	Nature. 1965 Aug 14;207(998):768. doi: 10.1038/207768b0.	1965
1	RCX-6						5963206	An XO-X ring X chromosome mosaicism in an individual with normal secondary sexual development	Bishop AM, Blank CE, Simpson K, Dewhurst CJ.	J Med Genet. 1966 Jun;3(2):129-33. doi: 10.1136/jmg.3.2.129.	1966
1	RCX-7						5301758	[Apropos of a cas of ring chromosome X]	Deminatti M, Maillard E, Fossati P,	Ann Genet. 1968 Mar;11(1):56-8.	1968

1	RCX-8	5404540	[Turner's syndrome with ring X chromosome]	Dallapiccola B, Zanardi F.	Rass Neurol Veg. 1969;23(5):243-55.	1969
1	RCX-9	168059	A case report of Turner's syndrome with ring X chromosome	Tanaka K, Otsuka Y, Fujii S, Morii H, Wada M, Fujita H.	Endocrinol Jpn. 1975 Apr;22(2):169-74. doi: 10.1507/endocrj1954.22.169.	1975
1	RCX-10	7443121	Turner phenotype in mother and daughter	Muasher S, Baramki TA, Diggs ES.	Obstet Gynecol. 1980 Dec;56(6):752-6.	1980
1	RCX-11	6958260	An infertile female with ring (X) chromosome constitution and evidence of ovulation	Smith A, Shale DJ, Dulk GD.	Aust N Z J Obstet Gynaecol. 1982 May;22(2):90-3. doi: 10.1111/j.1479-828x.1982.tb01411.x.	1982
2	RCX-12	6883789	Turner syndrome patients with a ring X chromosome	Berkovitz G, Stamberg J, Plotnick LP, Lanes R.	Clin Genet. 1983 Jun;23(6):447-53. doi: 10.1111/j.1399-0004.1983.tb01980.x.	1983
2	RCX-13	6152802	Gonadal dysgenesis and somatic stigmata in patients with 45,X/46,Xr(X) ring chromosome	Portuondo JA, Barral A, Neyro JL, Camarero MC, Roman MD, Uribarren	Int J Gynaecol Obstet. 1984 Aug;22(4):311-3. doi: 10.1016/0020-	1984
1	RCX-14	3758629	[A rare case of gonadal dysgenesis with 45, X-46, X ring X mosaic--case report]	Albert PJ, Gille J, Schmid M.	Geburtshilfe Frauenheilkd. 1986 Jul;46(7):473-4. doi: 10.1055/s-2008-1036240.	1986
1	RCX-15	3198130	A mosaic 45,X/46,X,r(?) karyotype investigated with X and Y centromere-specific probes using a non-autoradiographic in situ hybridization	Crolla JA, Llerena JC Jr.	Hum Genet. 1988 Dec;81(1):81-4. doi: 10.1007/BF00283735.	1988
0	RCX-16	2080999	High incidence of mental retardation in Turner syndrome patients with ring chromosome X formation	Fryns JP, Kleczkowska A, Van Den Berghe H.	Genet Couns. 1990;1(2):161-5.	1990
1	RCX-17	2106786	A ring X chromosome, 46,Y,r(X)(p22.33q28), as a cause of extreme short stature in a male	Ogata T, Matsuo N, Shimizu N.	Am J Med Genet. 1990 Feb;35(2):241-4. doi: 10.1002/ajmg.1320350219.	1990
2	RCX-18	1758399	[A report of 2 cases of Turner's syndrome with a ring X chromosome]	Migliori MV, Bartolotta E, Maurizi M, Bonazzi P, Cardinale G, Manunza V.	Minerva Pediatr. 1991 Sep;43(9):605-9.	1991
2	RCX-19	1999837	An investigation of ring and dicentric chromosomes found in three Turner's syndrome patients using DNA analysis and in situ hybridisation with X and Y chromosome specific	Cooper C, Crolla JA, Laister C, Johnston DI, Cooke P.	J Med Genet. 1991 Jan;28(1):6-9. doi: 10.1136/jmg.28.1.6.	1991
1	RCX-20	1339199	45,X/46,X,+r(X) can have a distinct phenotype different from Ullrich-Turner syndrome	Grompe M, Rao N, Elder FF, Caskey CT, Greenberg F.	Am J Med Genet. 1992 Jan 1;42(1):39-43. doi: 10.1002/ajmg.1320420110.	1992
5	RCX-21	1632446	Identification of the origin of ring/marker chromosomes in patients with Ullrich-Turner syndrome using X and Y specific alpha satellite DNA probes	Tharapel SA, Wilroy RS, Keath AM, Rivas ML, Tharapel AT.	Am J Med Genet. 1992 Mar 1;42(5):720-3. doi: 10.1002/ajmg.1320420519.	1992
1	RCX-22	1557312	Prenatal investigation of a 45,X/46,X,r(?) karyotype in amniocytes using fluorescence in situ hybridization with an X-centromeric probe	Bajalica S, Bui TH, Koch J, Bröndum-Nielsen K.	Prenat Diagn. 1992 Jan;12(1):61-4. doi: 10.1002/pd.1970120109.	1992
6	RCX-23	1415351	Ullrich-Turner syndrome with a small ring X chromosome and presence of mental retardation	Van Dyke DL, Wiktor A, Palmer CG, Miller DA, Witt M, Babu VR, Worsham MJ, Roberson JR, Weiss L.	Am J Med Genet. 1992 Aug 1;43(6):996-1005. doi: 10.1002/ajmg.1320430617.	1992
3	RCX-24	8326492	Three patients with ring (X) chromosomes and a severe phenotype	Dennis NR, Collins AL, Crolla JA, Cockwell AE, Fisher AM, Jacobs PA.	J Med Genet. 1993 Jun;30(6):482-6. doi: 10.1136/jmg.30.6.482.	1993
1	RCX-25	8225318	Molecular analysis of a ring chromosome X in a family with fragile X syndrome	Mornet E, Bogyo A, Deluchat C, Simon-Bouy B, Mathieu M, Thépot F, Grisard	Hum Genet. 1993 Oct;92(4):373-8. doi: 10.1007/BF01247338.	1993
1	RCX-26	8077037	FISH techniques in a Turner mosaic with ring X chromosome	Rajangam S, Lincoln S, Hegde S, Jayashree, Manjunath NA, Thomas IM.	Indian Pediatr. 1993 Dec;30(12):1451-4.	1993
8	RCX-27	8265665	Deficient transcription of XIST from tiny ring X chromosomes in females with severe phenotypes	Migeon BR, Luo S, Stasiowski BA, Jani M, Axelman J, Van Dyke DL, Weiss L, Jacobs PA, Yang-Feng TL,	Proc Natl Acad Sci U S A. 1993 Dec 15;90(24):12025-9. doi: 10.1073/pnas.90.24.12025.	1993
3	RCX-28	8079992	The severe phenotype of females with tiny ring X chromosomes is associated with inability of these chromosomes to undergo X inactivation	Migeon BR, Luo S, Jani M, Jeppesen P.	Am J Hum Genet. 1994 Sep;55(3):497-504.	1994
9	RCX-29	7966189	A comparison of the clinical and cytogenetic findings in nine patients with a ring (X) cell line and 16 45,X patients	Collins AL, Cockwell AE, Jacobs PA, Dennis NR.	J Med Genet. 1994 Jul;31(7):528-33. doi: 10.1136/jmg.31.7.528.	1994
1	RCX-30	7762970	An atypical Turner syndrome patient with ring X chromosome mosaicism	Cantú ES, Jacobs DF, Pai GS.	Ann Clin Lab Sci. 1995 Jan-Feb;25(1):60-5.	1995

1	RCX-31	7760319	Molecular cytogenetic characterisation of a small ring X chromosome in a Turner patient and in a male patient with congenital abnormalities: role of X inactivation	Callen DF, Eyre HJ, Dolman G, Garry-Battersby MB, McCreanor JR, Valeba A, McGill JJ.	J Med Genet. 1995 Feb;32(2):113-6. doi: 10.1136/jmg.32.2.113.	1995
1	RCX-32	9091352	A Turner syndrome woman with a ring X chromosome [45,X/46,X,r(X)(p22.3q27)] whose child also had a ring X chromosome	Uehara S, Nata M, Obara Y, Niinuma T, Funato T, Yajima A.	Fertil Steril. 1997 Mar;67(3):576-9. doi: 10.1016/s0015-0282(97)80091-x.	1997
1	RCX-33	9129739	Ring chromosome X in a child with manifestations of Kabuki syndrome	McGinniss MJ, Brown DH, Burke LW, Mascarello JT, Jones MC.	Am J Med Genet. 1997 May 2;70(1):37-42.	1997
1	RCX-34	9129738	Prune-belly syndrome and other anomalies in a stillborn fetus with a ring X chromosome lacking	Guillén DR, Lowichik A, Schneider NR, Cohen DS, Garcia S, Zinn AR.	Am J Med Genet. 1997 May 2;70(1):32-6. doi:	1997
1	RCX-35	9462277	Common variable immunodeficiency with CD4+ T lymphocytopenia and overproduction of soluble IL-2 receptor associated with Turner's syndrome and dorsal kyphoscoliosis	al-Attas RA, Rahi AH, Ahmed el-FE.	J Clin Pathol. 1997 Oct;50(10):876-9. doi: 10.1136/jcp.50.10.876.	1997
1	RCX-36	9737776	Microphthalmia with linear skin defects syndrome in a mosaic female infant with monosomy for the Xp22 region: molecular analysis of the Xp22 breakpoint and the X-inactivation pattern	Ogata T, Wakui K, Muroya K, Ohashi H, Matsuo N, Brown DM, Ishii T, Fukushima Y.	Hum Genet. 1998 Jul;103(1):51-6. doi: 10.1007/s004390050782.	1998
1	RCX-37	9832041	Severe phenotype resulting from an active ring X chromosome in a female with a complex karyotype: characterisation and replication study	Stavropoulou C, Mignon C, Delobel B, Moncla A, Depetris D, Croquette MF, Mattei MG.	J Med Genet. 1998 Nov;35(11):932-8. doi: 10.1136/jmg.35.11.932.	1998
5	RCX-38	10490708	Social, communicational, and behavioral deficits associated with ring X turner syndrome	El Abd S, Patton MA, Turk J, Hoey H, Howlin P.	Am J Med Genet. 1999 Oct 15;88(5):510-6. doi: 10.1002/(sici)1096-	1999
1	RCX-39	10766981	Mother and daughter with 45,X/46,X,r(X)(p22.3q28) and mental retardation: analysis of the X-inactivation patterns	Matsuo M, Muroya K, Nanao K, Hasegawa Y, Terasaki H, Kosaki K, Ogata T.	Am J Med Genet. 2000 Apr 10;91(4):267-72.	2000
2	RCX-40	10861682	Severe phenotypes associated with inactive ring X chromosomes	Migeon BR, Ausems M, Giltay J, Hasley-Royster C, Kazi E, Lydon TJ, Engelen JJ,	Am J Med Genet. 2000 Jul 3;93(1):52-7.	2000
1	RCX-41	11111386	[Familial occurrence of Turner syndrome]	Lukács Valéria H, Tardy Erika P, Molnár	Orv Hetil. 2000 Nov 5;141(45):2443-	2000
2	RCX-42	11155770	Autoimmune thyroiditis in children with Turner syndrome	Chang P, Tsai WY, Hou JW, Hsiao PH, Lee JS.	J Formos Med Assoc. 2000 Nov;99(11):823-6.	2000
0	RCX-43	11462660	[Active ring X chromosome]	Ogata T.	Ryoikibetsu Shokogun Shirizu.	2001
1	RCX-44	11313755	A Rett syndrome patient with a ring X chromosome: further evidence for skewing of X inactivation and heterogeneity in the aetiology of	Rosenberg C, Wouters CH, Szuhai K, Dorland R, Pearson P, Poll-The BT, Colombijn RM, Breuning M, Lindhout	Eur J Hum Genet. 2001 Mar;9(3):171-7. doi: 10.1038/sj.ejhg.5200604.	2001
1	RCX-45	11755101	Second trimester prenatal diagnosis of epignathus teratoma in ring X chromosome mosaicism with inactive ring X chromosome	Witters I, Moerman P, Louwagie D, Van Assche FA, Migeon BR, Fryns JP.	Ann Genet. 2001 Oct-Dec;44(4):179-82. doi: 10.1016/s0003-3995(01)01090-5.	2001
1	RCX-46	11484209	Kabuki syndrome-like features associated with a small ring chromosome X and XIST gene	Stankiewicz P, Thiele H, Giannakudis I, Schlicker M, Baldermann C, Krüger A,	Am J Med Genet. 2001 Aug 15;102(3):286-92. doi:	2001
1	RCX-47	11896455	Lack of expression of XIST from a small ring X chromosome containing the XIST locus in a girl with short stature, facial dysmorphism and developmental delay	Tomkins DJ, McDonald HL, Farrell SA, Brown CJ.	Eur J Hum Genet. 2002 Jan;10(1):44-51. doi: 10.1038/sj.ejhg.5200757.	2002
1	RCX-48	11941480	Molecular characterization of a ring X chromosome in a male with short stature	Ellison JW, Tekin M, Sikes KS, Yankowitz J, Shapiro L, Rappold GA,	Hum Genet. 2002 Apr;110(4):322-6. doi: 10.1007/s00439-002-0685-7.	2002
4	RCX-49	12900575	The proportion of cells with functional X disomy is associated with the severity of mental retardation in mosaic ring X Turner syndrome females	Kubota T, Wakui K, Nakamura T, Ohashi H, Watanabe Y, Yoshino M, Kida T, Okamoto N, Matsumura M,	Cytogenet Genome Res. 2002;99(1-4):276-84. doi: 10.1159/000071604.	2002
1	RCX-50	15337477	Turner syndrome female with a small ring X chromosome lacking the XIST, an unexpectedly mild phenotype and an atypical association with alopecia universalis	Bouayed Abdelmoula N, Portnoï MF, Amouri A, Arladan A, Chakroun M, Saad A, Hchicha M, Turki H, Rebai T.	Ann Genet. 2004 Jul-Sep;47(3):305-13. doi: 10.1016/j.anngen.2004.03.008.	2004
1	RCX-51	16007631	Microdeletion in the SHOX 3' region associated with skeletal phenotypes of Langer mesomelic dysplasia in a 45,X/46,X,r(X) infant and Leri-Weill dyschondrosteosis in her 46,XX mother: implication for the SHOX enhancer	Fukami M, Okuyama T, Yamamori S, Nishimura G, Ogata T.	Am J Med Genet A. 2005 Aug 15;137(1):72-6. doi: 10.1002/ajmg.a.30852.	2005
1	RCX-52	17252699	Mosaic Turner syndrome and hyperinsulinaemic hypoglycaemia.	Alkhayyat H, Christesen HB, Steer J, Stewart H, Brusgaard K, Hussain K	J Pediatr Endocrinol Metab. 2006 Dec;19(12):1451-7. doi:	2006

1	RCX-53						19653414	Kabuki make-up (Niikawa-Kuroki) syndrome with mosaicism ring chromosome X and incomplete XIST gene expression	Su PH, Kuo PL, Chen SJ, Chen JY, Yu JS, Liu YL, Kao IW.	Acta Paediatr Taiwan. 2007 Jan-Feb;48(1):28-31.	2007
1	RCX-54						18177863	Mosaic ring X chromosome in a case of secondary amenorrhea	Chrisoulidou A, Bili H, Georgiou E, Mavroudi S, Lazaridou AS.	Fertil Steril. 2008 Oct;90(4):1198.e19-21. doi:	2008
1	RCX-55						18639760	Becker muscular dystrophy with r(X) carrying an out-of-frame DMD deletion	Lee KA, Han SH, Choi JR, Chung JS, Choi YC.	Pediatr Neurol. 2008 Aug;39(2):129-32. doi: 10.1016/j.pediatrneurol.2008.05.002.	2008
1	RCX-56						19189708	Possible relationship between ring X chromosome and neonatal hypoglycemia	Goto M.	J Pediatr Endocrinol Metab. 2008 Nov;21(11):1103. doi: 10.1515/jpem.2008.21.11.1103.	2008
1	RCX-57						18925662	A small and active ring X chromosome in a female with features of Kabuki syndrome	Rodríguez L, Diego-Alvarez D, Lorda-Sanchez I, Gallardo FL, Martínez-Fernández ML, Arroyo-Muñoz ME,	Am J Med Genet A. 2008 Nov 1;146A(21):2816-21. doi: 10.1002/ajmg.a.32521.	2008
1	RCX-58						18655707	Mosaicism for r(X) and der(X)del(X)(p11.23)dup(X)(p11.21p11.22) provides insight into the possible mechanism of rearrangement	Shchelochkov OA, Cooper ML, Ou Z, Peacock S, Yatsenko SA, Brown CW, Fang P, Stankiewicz P, Cheung SW.	Mol Cytogenet. 2008 Jul 25;1:16. doi: 10.1186/1755-8166-1-16.	2008
4	RCX-59						19192353	Feasibility of fertility preservation in young females with Turner syndrome	Lau NM, Huang JY, MacDonald S, Elizur S, Gidoni Y, Holzer H, Chian RC, Tulandi T, Tan SL.	Reprod Biomed Online. 2009 Feb;18(2):290-5. doi: 10.1016/s1472-6483(10)60268-4.	2009
1	RCX-60						22410210	Barth syndrome in a female patient	Cosson L, Toutain A, Simard G, Kulik W, Matyas G, Guichet A, Blasco H, Maakaroun-Vermesse Z, Vaillant MC,	Mol Genet Metab. 2012 May;106(1):115-20. doi: 10.1016/j.ymgme.2012.01.015.	2012
1	RCX-61						22250632	Turner syndrome with a ring X chromosome and atypical skin manifestation: port wine stain	Onal H, Adal E, Ersen A, Onal Z.	Int J Dermatol. 2012 Feb;51(2):207-10. doi: 10.1111/j.1365-4632.2011.05031.x.	2012
1	RCX-62						26064751	Hyperinsulinemic Hypoglycaemia in a Turner Syndrome with Ring (X)	Cappella M, Graziani V, Pragliola A, Sensi A, Hussain K, Muratori C, Marchetti F.	Case Rep Pediatr. 2015;2015:561974. doi: 10.1155/2015/561974.	2015
2	RCX-63						27387888	Molecular cytogenetic characterization of two Turner syndrome patients with mosaic ring X	Chauhan P, Jaiswal SK, Lakhotia AR, Rai AK.	J Assist Reprod Genet. 2016 Sep;33(9):1161-8. doi:	2016
1	RCX-64						33020433	Coexistence of Growth Hormone Deficiency and Pituitary Microadenoma in a Child with Unique Mosaic Turner Syndrome: A Case Report and Literature Review	Park EG, Kim EJ, Kim EJ, Kim HY, Kim SH, Yang A.	Diagnostics (Basel). 2020 Oct 4;10(10):783. doi: 10.3390/diagnostics10100783.	2020
1	RCX-65	4 y	M	de novo		45,X/46,Y,r(X)	33872775	Hypospadias in ring X syndrome	Elghezal H, Alfayez K, Ben Abdallah I, Alfares A, Almazyad A, Al Jasser A, Almobadel N, Alsuhaibani O,	Eur J Med Genet. 2021 Apr 16:104225. doi: 10.1016/j.ejmg.2021.104225.	2021
1	RCX-66	17 y	F	na		45,X/46,X,r(X)	34987848	Decoding enigma: Turner syndrome with ring chromosome	Das D, Roy D, Basu K, Sarkar A	Oxf Med Case Reports. 2021 Dec 28;2021(11-12)	2021
1	RCX-67						34124982	Occurrence of Hypopituitarism in Tunisian Turner Syndrome patients: familial versus sporadic cases	Mnif-Feki M, Safi W, Bougacha-Elleuch N	Gynecol Endocrinol. 2021 Sep;37(9):848-852	
**111**	**68**										
1	RCY-1	33 y	M				5127018	Meiotic studies on a subfertile patient with a ring Y chromosome	A C Chandley, P Edmond	Cytogenetics, 1971;10(4):295-304. doi: 10.1159/000130149.	1971
1	RCY-2						4708485	Y ring chromosome associated with gonadoblastoma in situ	G Khudr, K Benirschke	Obstet Gynecol. 1973; 41(6):897-901	1973
1	RCY-3						4544085	Abnormalities of human sex chromosomes. I. A ring Y without mosaiciam.	J German, J L Simpson, G A McLemore J	Ann Genet. 1973 Dec;16(4):225-31.	1973
1	RCY-4	6 m	M				4703024	Sex chromosome anomalies detection and fluoresc	Fabris C, Franceschini P, Bogetti G, Ponz	Acta Paediatr Scand. 1973 May;62(3	1973
1	RCY-5						4135787	45,X-45,X, ace(?Yp)plus-46,X,r(Y) in a phenotypically normal newborn male	U Ruthner, E Golob	Humangenetik, 1974, 22(2):177-80	1974
1	RCY-6						4469774	A rare structural anomaly of the Y chromosome: Y r	E M Bühler, T Tsuchimoto, U K Bühler, G	Arch Genet. 1974;47(1):52-9	1974
1	RCY-7	30 y	M				965013	Ring Y chromosome: 45,X/46,Xr(Y) chromosome mosaicism in a phenotypically normal male with azoospermia	T Maeda, M Ohno, A Ishibashi, M Samej	Hum Genet , 1976 34(1):99-102	1976
1	RCY-8	53 y	M				953209	Ring Y chromosome without mosaicism	M G Wilson, R B Stein, J W Towner	Birth Defects Orig Artic Ser 1976;12(5);105-12	1976
1	RCY-9						914284	Nonmosaic 46,X,r(Y) karyotype with female phenotype	László J, Gaál M, Bósze P.	Hum Genet. 1977 Oct 14;38(3):351-6. doi: 10.1007/BF00402164.	1977
1	RCY-10						649173	A case of ring Y chromosome	Taillemite JL, van den Akker J, Portnoi MF, Le Porrier N, Marmor D, Bouillie J,	Hum Genet. 1978 May 16;42(1):89-91. doi: 10.1007/BF00291630.	1978
1	RCY-11						479854	[Structural abnormalities of the Y chromosome. Observations in ten cases]	Mattei JF, Mattei MG, Lucas C, Giraud F.	J Genet Hum. 1979 Mar;27(1):53-66.	1979

1	RCY-12	457111	Unstable ring Y chromosome in an aspermic male	Steinbach P, Fabry H, Scholz W.	Hum Genet. 1979 Apr 5;47(3):227-31. doi: 10.1007/BF00321013.	1979
1	RCY-13	7252090	[45X/46XY/46XrY mosaic with banding and the Turner phenotype (author's transl)]	Aubert L, Verdet C, Giraud F, Mattei JF.	J Gynecol Obstet Biol Reprod (Paris). 1981;10(1):57-60.	1981
1	RCY-14	6951928	A chromosomal survey of mentally retarded children in Taipei. IV. An azoospermic male with unstable ring Y chromosome	Lai HC, Wang S, Lai JS, Wuu KD.	Taiwan Yi Xue Hui Za Zhi. 1981 Dec;80(12):1327-31.	1981
4	RCY-15	2409888	G-11 staining in Turner's syndrome with mos 45,X/46,X,r(?)	de Almeida JC, Llerena JC Jr, Molina Gomes D, Rita Martins R, Jung M, Reis	Ann Genet. 1985;28(1):37-41.	1985
1	RCY-16	3706407	Ring Y chromosome	Mendez H.	Am J Med Genet. 1986 May;24(1):201-2. doi: 10.1002/ajmg.1320240126.	1986
0	RCY-17	3560166	Ring XY bivalent: a new phenomenon at metaphase I of meiosis in man	Chandley AC, Hargreave TB, McBeath S, Mitchell AR, Speed RM.	J Med Genet. 1987 Feb;24(2):101-6. doi: 10.1136/jmg.24.2.101.	1987
1	RCY-18	3320994	[Mosaicism 45,X/46,X,r/(Y) associated with mixed gonadal dysgenesis. Description of a case and review of the literature]	Bruni L, Gregory S, Boldrini R, Brinchi V, Ferrante E.	Pediatr Med Chir. 1987 Jul-Aug;9(4):495-7.	1987
1	RCY-19	2965862	[Down's syndrome with Turner mosaicism/Y chromosome in a ring: 46,X+21/47X(r)Y,+21]	Campos Tristán C, Vila Dupla S, Fernández Marín L, González MJ.	An Esp Pediatr. 1987 Dec;27(6):479-80.	1987
1	RCY-20	3265307	Giemsa-11 technique elucidating three structurally altered nonfluorescent Y chromosomes: r (Y), idic (Yp), dir tan dup (Yp)	Kosztolányi G.	Ann Genet. 1988;31(4):235-40.	1988
1	RCY-21	3057830	[A case of male pseudohermaphroditism with structural abnormalities of Y chromosome (ring Y)]	Watanabe H, Hachisuka Y, Watase H, Fushimi N, Ohtaguro K.	Hinyokika Kiyo. 1988 Aug;34(8):1469-74.	1988
1	RCY-22	3417311	Fluorescence in situ hybridization and Y ring chromosome	Kozma R, Fear C, Adinolfi M.	Hum Genet. 1988 Sep;80(1):95-6. doi: 10.1007/BF00451465.	1988
1	RCY-23	2709396	Identification and characterisation of a small marker chromosome using non-isotopic in situ hybridisation with X and Y specific probes	Crolla JA, Smith M, Docherty Z.	J Med Genet. 1989 Mar;26(3):192-4. doi: 10.1136/jmg.26.3.192.	1989
1	RCY-24	2354546	Phenotype of two males with abnormal Y chromosomes	Mićić M, Mićić S, Babić M, Diklić V.	Clin Genet. 1990 May;37(5):321-6. doi: 10.1111/j.1399-0004.1990.tb03513.x.	1990
1	RCY-25	2013232	Ring Y chromosome: molecular characterization by DNA probes	Pohlschmidt M, Rappold G, Krause M, Ahlert D, Hosenfeld D, Weissenbach J,	Cytogenet Cell Genet. 1991;56(2):65-8. doi:	1991
1	RCY-26	1424234	Ring Y chromosome: cytogenetic and molecular characterization	Wegner RD, Scherer G, Pohlschmidt M, L'Allemand D, Gal A.	Clin Genet. 1992 Aug;42(2):71-5. doi: 10.1111/j.1399-0004.1992.tb03142.x.	1992
1	RCY-27	8215218	Identification of ring Y chromosome: cytogenetic analysis, Southern blot and fluorescent in situ hybridization	Pezzolo A, Perroni L, Gimelli G, Arslanian A, Porta S, Gandullia P, Gandullia E.	Ann Genet. 1993;36(2):121-5.	1993
1	RCY-28	9056556	PCR and FISH analysis of a ring Y chromosome	Henegariu O, Kernek S, Keating MA, Palmer CG, Heerema NA.	Am J Med Genet. 1997 Mar 17;69(2):171-6. doi: 10.1002/(sici)1096-	1997
1	RCY-29	9286449	A case with mosaic di-, tetra-, and octacentric ring Y chromosomes	Henegariu O, Pescovitz OH, Vance GH, Verbrugge J, Heerema NA.	Am J Med Genet. 1997 Sep 5;71(4):426-9.	1997
2	RCY-30	9438785	Molecular investigation of two male subjects with short stature and a 45,X/46,X,ring(Y) karyotype	Sher ES, Addelston MB, Plotnick L, Urban MD, Berkovitz GD.	Horm Res. 1998;49(1):46-50. doi: 10.1159/000023125.	1998
2	RCY-31	10424817	Two male patients with ring Y: definition of an interval in Yq contributing to Turner syndrome	Tzancheva M, Kaneva R, Kumanov P, Williams G, Tyler-Smith C.	J Med Genet. 1999 Jul;36(7):549-53.	1999
1	RCY-32	10442333	45,X/46,X,r(Y) karyotype transmitted by father to son after intracytoplasmic sperm injection for oligospermia. A case report	Bofinger MK, Needham DF, Saldana LR, Sosnowski JP, Blough RI.	J Reprod Med. 1999 Jul;44(7):645-8.	1999
1	RCY-33	10951455	Novel ring chromosome composed of X- and Y-derived material in a girl with manifestations of Ullrich-Turner syndrome	Grass FS, Brown CA, Backeljauw PF, Lucas A, Brasington C, Gazak JM, Nakano S, Ostrowski RS, Spence JE.	Am J Med Genet. 2000 Aug 28;93(5):343-8.	2000
1	RCY-34	11334610	A case of ambiguous genitalia presenting with a 45,X/46,Xr(Y)(p11.2;q11.23)/47,X,idic(Y)(p11.2),idic(Y)(p11.2) karyotype.	Dundar M, Lowther G, Acar H, Kurtoglu S, Demiryilmaz F, Kucukaydin M	Ann Genet. 2001 Jan-Mar;44(1):5-8. doi: 10.1016/s0003-3995(00)01034-0.	2001
0	RCY-35	11755098	Y-chromosome mosaicism with ring Y-chromosome/idic(Y)(p11.2) and "normal" ovarian development	Fryns JP.	Ann Genet. 2001 Oct-Dec;44(4):169. doi: 10.1016/s0003-3995(01)01076-0.	2001

1	RCY-36	12749430	Meiotic behavior of the sex chromosomes in a 45,X/46,X,r(Y)/46,X,dic r(Y) patient whose semen was assessed by fluorescence in situ hybridization	Blanco J, Farreras A, Egozcue J, Vidal F.	Fertil Steril. 2003 Apr;79(4):913-8. doi: 10.1016/s0015-0282(02)04931-2.	2003
1	RCY-37	14986833	Ring Y chromosome in an azoospermic male with short stature: additional evidence for a distinct ring Y syndrome in non-mosaic patients?	Gisselsson D, Kristoffersson U, Giwercman A.	Clin Genet. 2003 Dec;64(6):519-21. doi: 10.1046/j.1399-0004.2003.00173.x.	2003
2	RCY-38	15214019	Ring (Y) in two azoospermic men	Lin YH, Lin YM, Lin YH, Chuang L, Wu SY, Kuo PL.	Am J Med Genet A. 2004 Jul 15;128A(2):209-13. doi:	2004
1	RCY-39	15378544	Unmasking 15q12 deletion using microarray-based comparative genomic hybridization in a mentally retarded boy with r(Y)	Kurosawa K, Harada N, Sosonkina N, Niikawa N, Matsumoto N, Saitoh S.	Am J Med Genet A. 2004 Oct 15;130A(3):322-4. doi: 10.1002/ajmg.a.30260.	2004
1	RCY-40	15528264	Mitotic and meiotic behaviour of a naturally transmitted ring Y chromosome: reproductive risk evaluation	Arnedo N, Nogués C, Bosch M, Templado C.	Hum Reprod. 2005 Feb;20(2):462-8. doi: 10.1093/humrep/deh598. Epub 2004 Nov 4.	2005
1	RCY-41	24790310	Short stature and turner skeletal features in an 11-year-old boy with a ring y chromosome missing the short stature homeobox containing gene	Tanaka M, Ohmizono Y.	Clin Pediatr Endocrinol. 2005;14(2):45-7. doi: 10.1297/cpe.14.45. Epub 2005 Aug 12.	2005
2	RCY-42	16359976	Mosaic ring Y chromosome in two normal healthy men with azoospermia	Bertini V, Canale D, Bicocchi MP, Simi P, Valetto A.	Fertil Steril. 2005 Dec;84(6):1744. doi: 10.1016/j.fertnstert.2005.06.034.	2005
1	RCY-43	16974088	Dynamic increase of a 45,X cell line in a patient with multicentric ring Y chromosomes	DesGroseilliers M, Fortin F, Lafrenière AM, Brochu P, Lemyre E, Lemieux N.	Cytogenet Genome Res. 2006;115(1):90-3. doi:	2006
2	RCY-44	17375527	Centromeric association of a microchromosome Y in two male patients	Domínguez MG, Vásquez AI, Troyo R, Ortiz-Aranda M, Padilla JR, Hernández-[illegible]	Genet Couns. 2006;17(4):413-9.	2006
1	RCY-45	17469055	Molecular cytogenetic analysis of a ring-Y infertile male patient	Carvalho FM, Wolfgramm EV, Degasperi I, Verbeno BM, Vianna BA,	Genet Mol Res. 2007 Mar 9;6(1):59-66.	2007
1	RCY-46	17606670	Gender identity disorders and bipolar disorder associated with the ring Y chromosome	Mouaffak F, Gallarda T, Baup N, Olié JP, Krebs MO.	Am J Psychiatry. 2007 Jul;164(7):1122-3. doi: 10.1176/ajp.2007.164.7.1122.	2007
5	RCY-47	18555994	Phenotypic spectrum of 45,X/46,XY males with a ring Y chromosome and bilaterally descended	Layman LC, Tho SP, Clark AD, Kulharya A, McDonough PG.	Fertil Steril. 2009 Mar;91(3):791-7. doi:	2009
0	RCY-48	18295215	Clinical implications of the detection of Y-chromosome mosaicism in Turner's syndrome: report of 3 cases	Bianco B, Nunes Lipay MV, Guedes AD, Verreschi IT.	Fertil Steril. 2008 Oct;90(4):1197.e17-20. doi: 10.1016/j.fertnstert.2007.09.014. Epub 2008 Mar 4.	2008
1	RCY-49	19752596	Mix gonadal dysgenesis associated with ring Y chromosome mosaics in a phenotypic male	Lopez-Valdes JA, Nieto K, Najera N, Cervantes A, Kofman-Alfaro S, Queipo	Sex Dev. 2009;3(4):177-82. doi: 10.1159/000228717. Epub 2009	2009
1	RCY-54	20966427	Chromosomal abnormalities in 2 cases of testicular failure	Chen X, Raca G, Laffin J, Babaian KN, Williams DH.	J Androl. 2011 May-Jun;32(3):226-31. doi: 10.2164/jandrol.110.010280. Epub 2010 Oct 21.	2011
1	RCY-50	21791323	Prenatal diagnosis and molecular cytogenetic characterization of a small marker chromosome derived from Y chromosome	Chen CP, Chen M, Ma GC, Chang SP, Chen YY, Wu PC, Chen LF, Wang W.	Taiwan J Obstet Gynecol. 2011 Jun;50(2):253-7. doi: 10.1016/j.tjog.2011.04.007.	2011
1	RCY-51	24052714	Molecular analysis of ring y chromosome in a 10-year-old boy with mixed gonadal dysgenesis and growth hormone deficiency	Milenkovic T, Guc-Scekic M, Zdravkovic D, Topic V, Liehr T, Joksic G, Radivojevic D, Lakic N.	Balkan J Med Genet. 2011 Dec;14(2):71-6. doi: 10.2478/v10034-011-0049-5.	2011
1	RCY-52	24551992	Genetic evaluation of an infertile male with a ring Y chromosome and SHOX deletion	Zamani AG, Tuncez E, Yildirim MS, Acar A.	Genet Couns. 2013;24(4):449-54.	2013
1	RCY-53	24335108	45,X/46,X,r(Y)/46,X,dic r(Y) karyotype in an azoospermic male: a case report	Dong Y, Yu XW, Wang RX, Li LL, Jiang YT, Liu RZ.	Cytogenet Genome Res. 2014;142(2):140-4. doi: 10.1159/000356467. Epub 2013 Dec 6.	2014
1	RCY-54	25511948	[Case of azoospermia patient with a chromosomal abnormality considered a ring Y chromosome]	Kuroda S, Yumura Y, Hamanoue H, Yasuda K, Yamanaka H, Sanjo H, Takeshima T, Kobayashi M, Kato Y,	Hinyokika Kiyo. 2014 Nov;60(11):583-6.	2014
**64**	**54**					

Table S2: Relative Frequency of Constitutional Ring Chromosomes (RCs) and Cytogenomic Findings in PubMed 1962-2022

RC	Case Count	Relative Frequency	No. Publications	PubMed Assessed	CMA	Complete RC	Simple del	Complex del/dup	Prenatal	TOP	SB	CP	Postnatal	Adult	Die	De novo	Mat	Pat
RC1	7	0.0068627	7	7	1		1		0				7		1	2		
RC2	14	0.0137255	13	12	4		4		1	1			11	1	1	9		1
RC3	11	0.0107843	12	9	2		2		0				9	2		6		
RC4	34	0.0333333	36	31	7	1	5	1	3	1	1	1	28	4	5	24		
RC5	13	0.0127451	10	10	1		1		0				10		1	8	1	
RC6	39	0.0382353	35	38	8		5	3	7	3	1	3	31	2	3	22		
RC7	21	0.0205882	24	20	3		3		1	1			19	4	3	9		
RC8	9	0.0088235	9	7	1			1	0				7	2		3	1	
RC9	32	0.0313725	32	25	8	1	4	3	2	2			23	4	3	10		
RC10	18	0.0176471	17	17	6		6		1	1			16	1	2	11		
RC11	17	0.0166667	14	17	6		5	1	1	1			16	3	2	8	2	
RC12	7	0.0068627	7	7	1			1	0				7	4		2		
RC13	82	0.0803922	71	63	20	1	13	6	10	8	2		53	7	3	29	1	
RC14	73	0.0715686	66	51	7	2	4	1	3	3			48	4	2	13	1	
RC15	75	0.0735294	62	64	14		11	3	7	4	3		57	13	1	32	5	
RC16	5	0.004902	5	5	0				0				5	1		3		
RC17	20	0.0196078	23	17	3	1	2		1	1			16	4	1	8		
RC18	110	0.1078431	92	95	25		20	5	8	7		1	87	15	3	28	5	
RC19	6	0.0058824	6	6	1	1			1		1		5	2		2	2	
RC20	118	0.1156863	80	100	6	3	2	1	2	1		1	98	34	1	14	6	
RC21	72	0.0705882	74	60	18	1	11	6	8	2		6	52	16	2	21	11	1
RC22	71	0.0696078	61	66	21	2	17	2	6	6			60	17	1	21	2	1
Subtotal	854		756	727	163	13	116	34	62	42	8	12	665	140	35	285	37	3
RCX	111	0.1088235	68															
RCY	64	0.0627451	54															
Subtotal	175		122															
Total	1029		878															

*CMA, Chromosome microarray analysis; TOP, termination of pregnancy; SB, stillbirth; CP, continue pregnancy; mat, maternal; pat, paternal
